# Mechanochemical Synthesis and Biological Evaluation of Novel Isoniazid Derivatives with Potent Antitubercular Activity

**DOI:** 10.3390/molecules22091457

**Published:** 2017-09-01

**Authors:** Paulo F. M. Oliveira, Brigitte Guidetti, Alain Chamayou, Christiane André-Barrès, Jan Madacki, Jana Korduláková, Giorgia Mori, Beatrice Silvia Orena, Laurent Roberto Chiarelli, Maria Rosalia Pasca, Christian Lherbet, Chantal Carayon, Stéphane Massou, Michel Baron, Michel Baltas

**Affiliations:** 1 Department of Process Engineering, Université de Toulouse, Mines-Albi, CNRS UMR 5302, Centre RAPSODEE, Campus Jarlard, 81013 Albi, France; paul_marqs@hotmail.com (P.F.M.O.); alain.chamayou@mines-albi.fr (A.C.); baron@mines-albi.fr (M.B.); 2 Department of Chemistry, Université de Toulouse, UPS, CNRS UMR 5068, LSPCMIB, 118 Route de Narbonne, 31062 Toulouse, France; guidetti@chimie.ups-tlse.fr (B.G.); candre@chimie.ups-tlse.fr (C.A.-B.); christian.lherbet@itav.fr (C.L.); andre@chimie.ups-tlse.fr (C.C.); massou@chimie.ups-tlse.fr (S.M.); 3 CNRS, Laboratoire de Synthèse et Physico-Chimie de Molécules d’Intérêt Biologique, LSPCMIB, UMR-5068, 118 Route de Narbonne, 31062 Toulouse, France; 4 Department of Biochemistry, Comenius University in Bratislava, Faculty of Natural Sciences, Mlynská Dolina, Ilkovičova 6, 84215 Bratislava, Slovakia; jan.madacki@gmail.com; 5 Department of Biology and Biotechnology “Lazzaro Spallanzani”, University of Pavia; via Ferrata 1, 27100 Pavia, Italy; giorgia.mori@unipv.it (G.M.); beatricesilvia.orena01@universitadipavia.it (B.S.O.); laurent.chiarelli@unipv.it (L.R.C.)

**Keywords:** *Mycobacterium tuberculosis*, mechanochemistry, hydrazone

## Abstract

A series of isoniazid derivatives bearing a phenolic or heteroaromatic coupled frame were obtained by mechanochemical means. Their pH stability and their structural (conformer/isomer) analysis were checked. The activity of prepared derivatives against *Mycobacterium tuberculosis* cell growth was evaluated. Some compounds such as phenolic hydrazine **1a** and almost all heteroaromatic ones, especially **2**, **5** and **7**, are more active than isoniazid, and their activity against some *M. tuberculosis* MDR clinical isolates was determined. Compounds **1a** and **7** present a selectivity index >1400 evaluated on MRC5 human fibroblast cells. The mechanism of action of selected hydrazones was demonstrated to block mycolic acid synthesis due to InhA inhibition inside the mycobacterial cell.

## 1. Introduction

**Figure 1:**
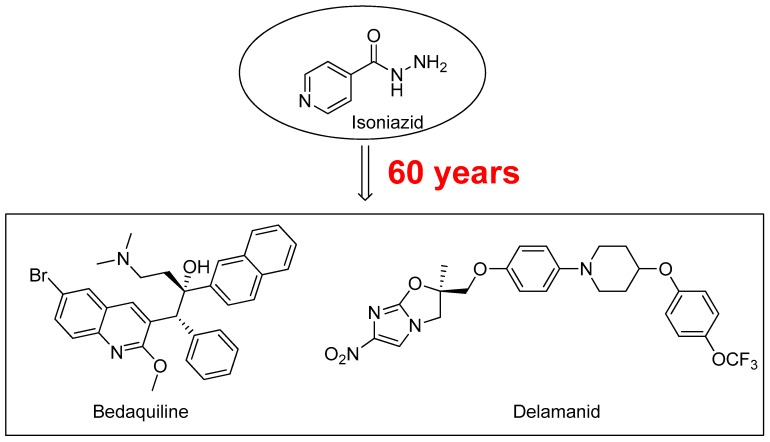
Antitubercular drug Isoniazid (1952), and Bedaquiline and Delamanid, two new compounds approved for the treatment of MDR-TB.

Tuberculosis (TB), caused by *Mycobacterium tuberculosis* (*M.tb*), represents an enduring, deadly infectious disease worldwide. According to the World Health Organization (WHO), one third of the global population is infected with *M.tb*. In comparison with other diseases caused by a single infectious agent, TB is the second leading cause of mortality. It is estimated that in 2015 TB killed 1.8 million people, mainly in underdeveloped countries [1]. New effective drugs for the treatment of TB are necessary firstly to reduce the duration of TB treatment and, secondly, for the treatment of *M.tb* multidrug-resistant (MDR) [2,3], extensively-drug resistant (XDR) [4], and totally-drug resistant (TDR) strains [5]. In the recent years, two molecules bedaquiline and delamanid (Figure 1) have been approved for the treatment of MDR-TB when an effective treatment regimen is not otherwise available [6,7].

One of the main known drug targets to fight *M.tb* growth is the enoyl-ACP reductase or InhA. This well-known enzyme is already the indirect target of Isoniazid (INH), a front-line prodrug used clinically to treat TB. INH remains a key component in all multiple drug treatment regiments recommended by the WHO even if *M.tb* resistant isolates have been rapidly generated during monotherapy or inappropriate treatment. Hence, improvement of INH molecule by introducing chemical modifications in its core structure in order to enhance biological response (prodrug, increase of bioavailability, and membrane permeability) continues to be an interesting scientific challenge. Recently, for example, compounds containing isonicotinoyl moiety with potential dual inhibition targeting FabG4 and HtdX, were successfully characterized [8].

Hydrazide-hydrazone derivatives have long attracted attention because of their wide range of applications in medicinal chemistry [9,10,11,12]. Hydrazide and hydrazone derivatives showed strong antioxidant and radical scavenging properties, while others ones displayed potent anticancer, antimicrobial, anticonvulsant or anti-inflammatory activities in vitro [9]. Recently, interesting anti-mycobacterial activities were reported about the following derivatives: guanylhydrazones [13], *trans*-cinnamic acid hydrazides derivatives [14], fluorine containing hydrazones [15], sulfonyl-hydrazones [16], and *L*-proline derived hydrazones [17].

Moreover, isoniazid-related hydrazones showed similar or better efficiency than the INH [18]. Isonicotinoylhydrazone derivatives were also synthesized and evaluated as anti-mycobacterial agents [19,20]. Interestingly, vanillic acylhydrazones were reported as potential β-keto acyl carrier protein synthase III (FabH) inhibitors [21]. Schiff bases of isoniazid, considered as chemical modification that can block *N*-acetylation of INH, showed good activity in vitro and in vivo and in some cases low toxicity [22,23]. In 2008, an extended study reported a quantitative structure activity relationships (QSAR) of a large hydrazide family for the developing of antitubercular compounds [24]. Finally, some researchers focused on establishing a predictive QSAR model for different INH derivatives including isonicotinoylhydrazones [25].

Our group is involved both in synthesizing new antitubercular compounds, including cinnamic acid derivatives [26,27,28,29], triazoles [30,31,32,33], pyrrolidines [34,35,36], semicarbazones and hydrazine/hydrazones [37], and in searching for new and innovative synthetic reactions. We have recently reported the solvent-free mechanosynthesis of a series of hydrazones [38,39]. Mechanochemistry has been used for a long time for the chemical and physicochemical transformations of inorganic materials to generate all states of aggregation produced by the effect of mechanical energy [40]. More recently, the mechanical energy has been used to synthesize organic molecules in milling devices [41,42,43,44,45,46,47,48,49,50,51,52].

Considering the pharmaceutical area [53], the mechanical action was used in particular to develop nitrogen-containing heterocycles, well represented in many therapeutic classes. For example, phthalazoles [54], phenazines [55], pyrazoles, pyridazinones [56], and pyrroles [57] were obtained by mechanosynthesis. Non-heterocyclic nitrogen-containing molecules were also synthesized under mechanical solid-state and solvent-free conditions, including imines, azomethines [58,59], azines [56], enamines and hydrazones [60,61,62].

In this work, this methodology was used in order to synthesize hydrazones under solvent-free conditions, in particular the isonicotinoyl ones. Thus, herein we report the synthesis of a series of phenol and hetero aryl isonicotinoylhydrazones through mechanochemistry and the evaluation of their *anti*-tuberculosis activities.

## 2. Results and Discussion

### 2.1. Chemistry

#### Mechanosynthesis of Isonicotinoyl Hydrazones

**Figure 2:**
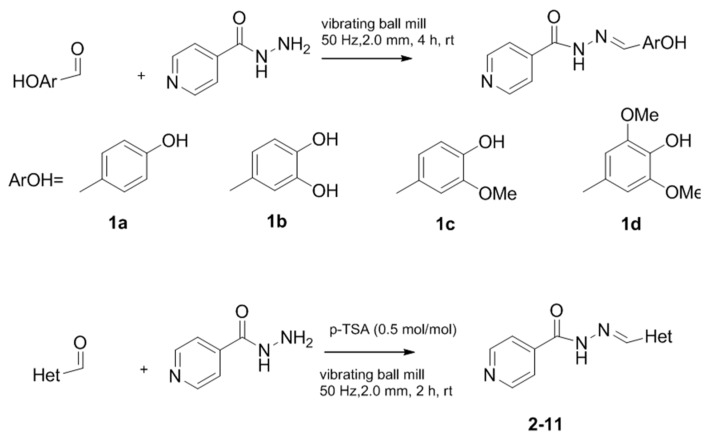
Isonicotinoyl hydrazones synthesized by co-grinding of isoniazide and an aldehyde.

The classical methods to synthesize hydrazones are generally carried out at low concentration, and require times from 3 to 24 h or even 48 h under reflux of toluene or ethanol in order to obtain good yields. We previously employed the vibratory mill Pulverisette 0 (P0) (Fritsch, Germany) to synthesize phenolic hydrazones mechanochemically [38]. A comparative study has been therein reported with various hydrazides, among them the isoniazid and phenolic aldehydes leading to compounds already described **1a**–**d** (Figure 2).

We decided to synthesize a series of isonicotinoylhydrazones derivatives bearing various *N*-heterocyclic indole, indazole or imidazole moieties using the mechanochemical approach. The corresponding aldehydes were selected because of the importance of these *N*-heterocyclic fragments in a large number of natural or synthetic biologically active molecules. Indeed, compounds bearing these frames may exhibit various activities, i.e., antibacterial, anticancer, antioxidant, anti-inflammatory, anti-diabetic, antiviral, anti-proliferative, antituberculosis, antispermatogenic or antipsychotic activities [63,64,65,66,67].

The reaction described for phenolic compounds was firstly used with the 4-methyl-5-imidazolecarboxaldehyde as model of heterocyclic aldehyde. However, in spite of the milling was carried out by up to eight hours, TLC and NMR analysis showed incomplete conversion. In that respect, we evaluated the efficiency of the reaction in acidic media.

The reaction was thus carried out in the presence of AlCl_3_ or of *p*-toluenesulfonic acid (*p*-TSA); the latter one showed the best result when using 50% mol of *p*-TSA. TLC showed the consumption of the reagents and the consequent appearance of the hydrazone, which was confirmed by ^1^H- and ^13^C-NMR analysis.

The hydrazones listed in Table 1 were thus obtained with a high transformation ratio in grinding times of 2 h.

It is still important to mention that, differently from the phenolic hydrazones, a melting was produced when the *p*-TSA was added, and, therefore, the reaction was not fully in solid-state. The formation of a fluid phase is vastly found for solid mixtures (eutectic melting) [68] and surely contributed to reach high conversions in short times of grinding for these hydrazones.

### 2.2. Structural Analysis of Isonicotinoyl Hydrazones by DFT and NMR: Determination of the Free Activation Energy (ΔG^≠^) between Conformers of Selected Compounds ***1a*** and ***5***

The structures of all the hydrazones were identified and fully characterized by ^1^H- and ^13^C-NMR, MS and HRMS, FTIR and UV-vis (see Supplementary Materials).

**Figure 3:**
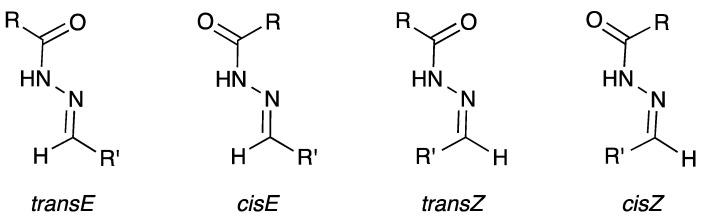
*E/Z*-configurational isomers and *cis/trans* amide conformers for *N*-acylhydrazones.

Concerning the NMR data, all isonicotinoyl hydrazones showed the presence of conformers in dimethylsulfoxide (DMSO) solution. NMR spectral and theoretical studies previously demonstrated that acylhydrazones generally exist predominantly or solely as a mixture of isomers [69,70,71]. In theory, *N*-acylhydrazones may exist with four possible arrangements in respect to (*E/Z*)-configurational isomers relative to the C=N bond and (*E’/Z’*)-rotamers caused by inversion of the amide bonds C(O)NH, here named *cis/trans* amide conformers (Figure 3) [69,72].

Although the four forms were considered, *E/Z* isomerization is generally not observed and the *Z* geometric isomers are absent or present only in poor part. An exception is for R’ = 2-pyridyl, in which strong intramolecular hydrogen bonds are present in the (*Z*)-form [70] mainly in less polar solvents.

While all isonicotinoyl hydrazones showed the presence of conformers in DMSO solution, for study purposes, two derivatives were chosen to be further investigated: phenolic hydrazone (**1a**) and indazole derivative (**5**). Both of them showed two sets of signals indicating the possibility of equilibrium between rotamers in solution. Theoretical assessment of the existence of the isomers was carried out.

The four structures of *Z/E* geometrical isomers and *cis/trans* amide conformers of **1a** were modeled by Density Functional theory (DFT), using Gaussian 09, firstly at HF/STO-3G level. The *Z* conformers were found higher in energy than the *E* ones, around 6 kcal/mol (see Table S1 in the Supplementary Materials). Thus, only the *cisE* and *transE*-isomers were then modeled at the B3LYP/6-31+G(d,p) level and the frequencies calculations were performed on the optimized geometries at 298 K, showing all positive frequencies and allowing evaluation of the Gibbs free energy.

Following Boltzmann distribution, (*P_i_/P_j_ = exp((G_j_ − G_i_)/k_B_T*), *cisE*-isomer was present at 92% and *transE* at 8% in the gas phase, whereas in DMSO, using the polarizable solvent continuum model (SMD), the ratio was inverted: *cis/trans*: 6/94 (Table 2).

A transition state (TS-**1a**) was found between *transE* and *cisE*, characterized by its imaginary frequency at −115.79 cm^−1^. The difference between Gibbs free energy of the transition state and the energy of the *cis*-*E*-isomer (*ΔG^≠^*) was equal to 17.63 kcal·mol^−1^.

The NMR chemical shift calculations were then performed at B3LYP/6-311+ (2d,p) level, using the DMSO polarizable continuum model (PCM). Isotropic shielding constants (σ) for ^1^H and ^13^C nuclei were transformed in chemical shift (δ) using linear regression procedure proposed by Lodewyk [73]. Calculated values are presented in the Supplementary Materials (Table S2). By comparing calculated values and experimental ones, we unambiguously concluded that the *transE*-isomer is the major one. This result is also in agreement with the calculated Gibbs free energy values in DMSO, at 298 K, as *transE*-isomer is −1.68 kcal/mol lower than the *cis* (ratio *cis*/*trans* around 6/94).

This activation energy can also be measured by ^1^H-NMR analysis, by determining the coalescence temperature *T_c_*.

At the coalescence temperature *T_c_*

*k_exch_* = πΔν/√2 = 2.22·Δν (Hz)
*k*_exch_ = *(k_B_*·*T_c_/h)exp(−ΔG^≠^/RT_c_)* (Eyringss equation)
ΔG^≠^ = *−RT_c_Ln(k_exch_*·*h/k_B_*·*T_c_)*

with *k_B_* = 1.38 × 10^−23^ kJ/K, *h* = 6.626 × 10^−34^ J·s, and *R* = 8.314 J·mol^−1^·K^−1^.

The ^1^H-NMR study at increasing temperatures (from 298 K to 388 K) allowed the determination of coalescence temperatures of the same signals of each isomer (Figure S1). Correlation of the coalescence temperatures *T_c_* with the difference in chemical shift of the signals led to *ΔG^≠^* following the precedent equation described (Supplementary Materials, Table S3). The mean value obtained on several signals led to *ΔG^≠^ =* 17.58 kcal·mol^−1^. This experimental value is in agreement with the calculated one of 17.63 kcal·mol^−1^ at the B3LYP/6-31+G(d,p) level.

The same study was performed on compound **5**, which possesses eight isomers. As previously described, the optimization of geometries on the height isomers at the HF/STO-3G showed that the *Z* isomers are higher in energy (6–7.5 kcal/mol) (Supplementary Materials, Table S4). Tsshus, the calculations were carried out at the B3LYP/6-31+G(d,p) level on the four isomers: *cis* and *trans E*-isomers and the two rotamers, in the gas phase and in the DMSO modeled by the SMD polarizable continuum model. Boltzmann analysis was used to determine the relative distribution of each conformer both in the gas phase and in the DMSO continuum model.

In the gas phase, *cis* compounds are the major ones, although, in the DMSO continuum, *trans* isomers are the major ones: *transE*: 80%, *cisE*: 20% (Table 3).

^1^H- and ^13^C-NMR chemical shifts were calculated as previously described at the B3LYP/6-311+G(2d,p) using DMSO continuum model (PCM) and taking into account the Boltzmann distribution of the two conformers for each isomer, which means *transE*-1/*transE*-2: 35%/65%, and *cisE*-1/*cisE*-2: 8%/92%. The comparison of calculated chemical shifts and experimental ones of both ^1^H and ^13^C (Supplementary Materials, Figure S2 and Table S5), showed that *trans* isomer is the major compound in DMSO.

The activation energy was evaluated by ^1^H-NMR analysis at increasing temperatures (from 298 K to 388 K) and was evaluated at 17.8 kcal/mol (Supplementary Materials, Table S6) in the same order as for compound **1a**.

These observations are important as restricted rotations might have an impact on pharmacological properties.

### 2.3. Physicochemical Studies of Some Isonicotinoyl Hydrazones

#### 2.3.1. Hydrolytic Stability

Stability studies were carried out in order to confirm that biological activities evidenced for isonicotinoyl hydrazones arise from the tested compounds, and not from the hydrolysis of the imine bond. UV-vis spectrophotometry at the λ_max_ of absorbance of the related molecule was used to monitor the stability of the most prominent synthesized compounds. The study was carried out for all compounds; here, we present the results for some of them while all other data are in the Supplementary Materials. Table 4 summarizes the conditions used and the stability results observed for **1a**–**1d**, **5** and **7** compounds.

As expected, UV-vis spectroscopy demonstrated that all the tested compounds are stable for a prolonged time (up to 22 h–7 days) and no significant decomposition was observed. Only **5** showed a very small reduction (3%) of the absorbance after 20 h. It is known that hydrazones possess greater intrinsic hydrolytic stability than that of imines. In addition to the contribution of the NH-N=C in electron delocalization, the resonance forms in acylhydrazones increase the negative-charge on the C=N and thus, reduce its electrophilicity and the affinity to the nucleophile attack from water [74]. Furthermore, the repulsion of the lone pairs of the NH-N can be relieved in the conjugates [75].

Moreover, the stability of compound **1d** was further assessed upon incubation in DMSO, the solvent used for biological assays, by mass spectrometry analysis; in addition, no relevant peaks corresponding to possibly released INH have been detected (Figure S3).

#### 2.3.2. p*K*_a_ Determination

**Figure 4:**
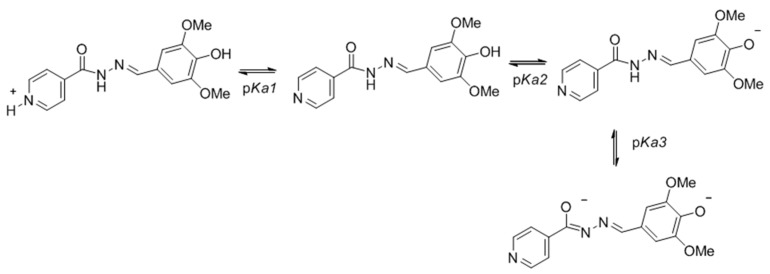
Protonation states of the hydrazone **1d** as function of pH.

The acid dissociation constant (*K*_a_, or more commonly expressed by p*K*_a_) is a very important physicochemical parameter in a wide range of research areas, including the development of active molecules due to solubility issues. The p*K*_a_ of the molecules studied for stability could also be determined by using UV-vis spectrometry based on the variation of the absorbance as function of the pH due to the presence of chromophores close to the ionization site of the molecules. The molar absorptivity varies according to the conjugation forms that dynamically change when and where the molecule is charged by the effect of pH and protonation/dissociation. As an example, the different protonation states for the hydrazone **1d** are shown in Figure 4.

**Figure 5:**
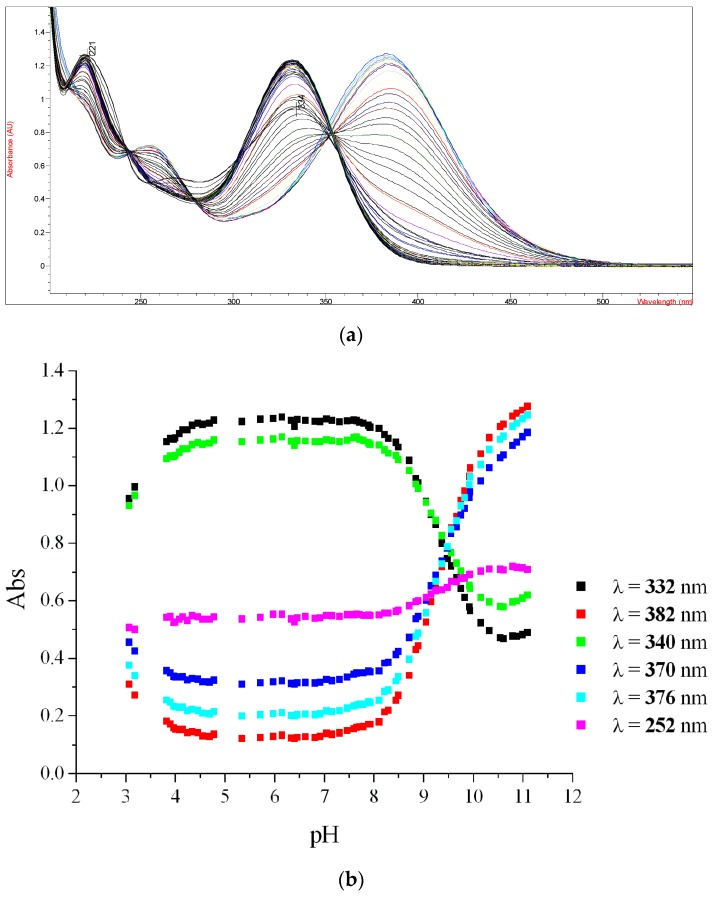
The variations on the UV-vis spectra for compound **1d** and the resulting plot for six wavelength values. (**a**) UV-vis spectra obtained for compound **1d** as function of pH variation; (**b**) Plots of absorbance for six wavelength values (λ/nm) as function of pH for compound **1d**.

For experimental p*K*_a_ determination, the molecules were solubilized in hydroalcoholic solutions at ethanol 28% and the ionic strength was maintained by the addition of KCl (0.1 M). The pH was adjusted with concentrated solutions of KOH and HCl. The UV spectra were recorded at each pH value and, at least three wavelengths were monitored. The absorbance variation was plotted as function of the pH. As an example, the variations on the UV-vis spectra for compound **1d** and the resulting plot for six wavelength values are shown in Figure 5a,b, respectively (all other spectra are present in Figures S4–S8). The protonation/dissociation of the molecule is accompanied by a variation in the absorbance (Figure 5a) and is graphically represented for six wavelength values as function of pH in Figure 5b.

The inflection points correspond to the change in that protonated/dissociated state of the molecule, and, therefore, the pH at that point amounts to the p*K*_a_. Thus, by using this method, the p*K*_a_ values of **1d** were determined [76]. The same protocol was applied for the other molecules and the resulted p*K*_a_ are presented in Table 5, as an average value from three different λ.

An inflection point was always observed close to pH 3, which is related with the protonation of 4-pyridinic nitrogen of isoniazid moiety (p*K*_a2_ of isoniazid = 3.5), and corresponds to p*K*_a1_ of the hydrazones. The p*K*_a2_ of the molecules ranges from 7.7 to 11. It means that they are in dissociated form from milder (**1b**) to stronger (**7**) basic conditions.

### 2.4. Biology of Mechanochemically Synthesized Hydrazones

#### 2.4.1. InhA Inhibition Assay

InhA, the NADH-dependent fatty acid biosynthesis (FAS-II) enoylreductase from *M. tuberculosis*, has emerged as a promising drug target due to its vital role in synthesis of mycolic acids. InhA is the main target of Isoniazid [77,78,79,80]. Recombinant *M.tb* InhA was expressed in *E. coli* and subsequently purified.

The selected synthetic compounds, corresponding to the phenolic isonicotinoyl hydrazones **1a**–**d** and the corresponding heterocyclic ones **2**–**11** series, were evaluated in vitro for the inhibition of *M.tb* InhA activity at 50 μM by applying a previously described method [81] (Table 6).

Considering the four phenolic derivatives **1a**–**d**, the tri-substituted one (**1d**) presents the better InhA inhibition activity with 64% value at 50 μM. Derivatives **1a**–**c** are less potent with 45–54% InhA inhibition range at 50 μM. Under these conditions, 99% inhibition is obtained for Triclosan (TCL).

Concerning the heterocyclic isonicotinoyl hydrazones, compounds **2**, **3** and **11** were difficult to evaluate due to solubility issues at high concentrations. Compound **5** is a very poor inhibitor (19% InhA inhibition), while compounds **4**, **6** and **7** may be considered as poor inhibitors with 32%, 43% and 39% values, respectively. Compound **10** is the derivative exhibiting the highest inhibition of InhA enzyme with 79% at 50 μM. In comparing isonicotinoyl derivatives derived from indoles (compounds **6**–**10**), we can notice that those possessing an indol-3-yl frame have the same activities with inhibition values between 32% and 43%. Compounds **6** and **8** differing by one methyl group (position 2 of the indol-3-yl frame) have the same inhibition values. On the contrary, compound **10** possessing an indole-2-yl frame is two-fold more active (79% of inhibition). Concerning the imidazole and indazole derivatives, we might hypothesize that the striking differences in activities could arise by a better positioning of the compounds **2** and **4** guided by their nitrogen atoms on their heterocyclic parts.

#### 2.4.2. Activity of Phenolic Isonicotinoyl Hydrazones (**1a**–**d**) against *M.tb* Cell Growth

The determination of the minimal inhibitory concentration (MIC) was performed using *M.tb* H37Rv strain and INH as control (Table 7).

It is noteworthy that a series of phenolic hydrazones bearing the four phenolic frames (**a**–**d**), previously synthetized by mechanochemical means were already tested against *M.tb*. cell growth [38]. Among the different hydrazines (isoniazid, hydralazine, 2-hydrazino-benzothiazole, 3-aminorhodanine, benzyl carbazate and benzhydrazide), used to form the corresponding hydrazones, only the isonicotinoyl derivatives were active against *M.tb* growth, whilst the other compounds were not effective (MIC > 30 μM; data not shown, but reported in Reference [39]).

Isonicotinoyl derivatives **1a**–**d** showed good anti-mycobacterial activity with 0.0125 or 0.125 μg/mL MIC values. Among these four, those bearing two or more substituents *ortho* to the phenolic function presented potent activities with a MIC value of 0.125 μg/mL, which is five times higher than that of INH. Compound **1a**, synthesized from *p*-hydroxybenzaldehyde and INH, is the most potent derivative with a MIC value of 0.0125 μg/mL (0.05 μM), which is 2–4 times lower than that of INH (0.025 μg/mL, 0.18 μM).

#### 2.4.3. *M.tb* H37Rv Growth Inhibition Assays of Nitrogen Heterocyclic Hydrazones (**2**–**11**)

The MIC values of the series of hydrazones**,** bearing the INH moiety coupled with different *N*-heterocyclic aldehydes, were also determined (Table 8).

All four imidazole and indazole derivatives (**2**–**5**) were 1.5–3 times more effective than isoniazid, while their InhA inhibition activities were much lower to inexistent with activities against *M.tb* growth, in the range of 0.11–0.23 μM. For indole derivatives, nitro substitution on the aromatic ring (compound **11**) or methyl substitution on the indole ring (compound **6**), compromised the anti-TB activity. While the bromo derivative **9** had the same activity as INH (0.36 μM), compounds **7**, **8** and **10** presented better MIC values than INH. Interestingly, the azaindole derivative **7** showed the highest anti-TB activity in this series and was found as active as the phenolic compound **1a** (MIC = 0.015 μg/mL/0.056 μM).

Finally, we can notice that all active compounds here presented, can show different lipophilic values as given by their LogP values, probably supporting the inference that there is no clear relationship between lipophilicity and in vitro activity as pointed also by others [25].

The resistance to the current tested drugs (first- and second-line) remains a very serious problem, mostly resulting from *inhA* and *kat*G mutations [82] and culminates in the occurrence of *M.tb* multidrug-resistant (MDR) strains. Owing the good results obtained for the herein studied INH-derived hydrazones, we tested them against a *M.tb* multidrug-resistant clinical isolate (IC2; resistant to streptomycin, INH, rifampicin, ethambutol, pyrazinamide, ethionamide, and capreomicin) (Table 9). The indazole and indole derivatives were not active against IC2 isolate (MICs > 10 μg/mL), except for the nitro derivative **11**. The imidazole containing derivatives **2** and **3** were poor active (MIC = 5–10 μg/mL). By comparing results for compounds **10** and **11**, it appears that the nitro substituent improves the activity against IC2 clinical isolate. Interestingly, the phenolic derivatives **1a** and **1b** presented the best activities against the MDR isolate, with MIC of 2.5 μg/mL (10.36 μM) and 1 μg/mL (3.89 μM), respectively.

#### 2.4.4. Cytotoxicity and Selectivity Index Determination

Cytotoxicities of all compounds bearing the INH moiety were also evaluated on MRC5 human fibroblast cells. Almost all compounds tested presented LD_50_ values above 80 μM (Table 10), with the exception of **1b** (LD_50_ = 36.3 μM). The LD_50_ evaluation is essential to determine the selectivity index (SI), which indicates the best candidates in terms of high biological activity against the target and low cytotoxicity. The SI presented in Table 10 are the ratio between LD_50_ and the in vitro MIC value against *M.tb* H37Rv previously obtained.

Apart from compound **1b**, due to its high toxicity, the phenolic hydrazones presented good selectivities higher than 170. A great result was obtained for **1a** which has the lowest MIC value (0.0125 μg/mL), LD_50_ > 80 μM and the highest SI (>1600).

The *N*-heterocyclic INH derivatives **2**–**11** presented good SI values, with the exception of **6** and **11**, which possessed the highest MIC values. Compounds **4**, **8**, **9** and **10** conducted to comparable SI. Compound **10** must be considered (MIC = 0.06 μg/mL, LD_50_ = 71.4 μM and SI = 310), mainly due to its InhA inhibition of 78%. Great SI values closer to 600 are found for **2** and **3** but, nonetheless, some reservation must be taken due to the poor solubility of these molecules. Finally, the SI of compound **5** is higher than 727 and **7** is highlighted with MIC = 0.015 μg/mL and an SI higher than 1469.

#### 2.4.5. In Search for the Molecular Target of Prepared Hydrazones in Mycobacteria

**Figure 6:**
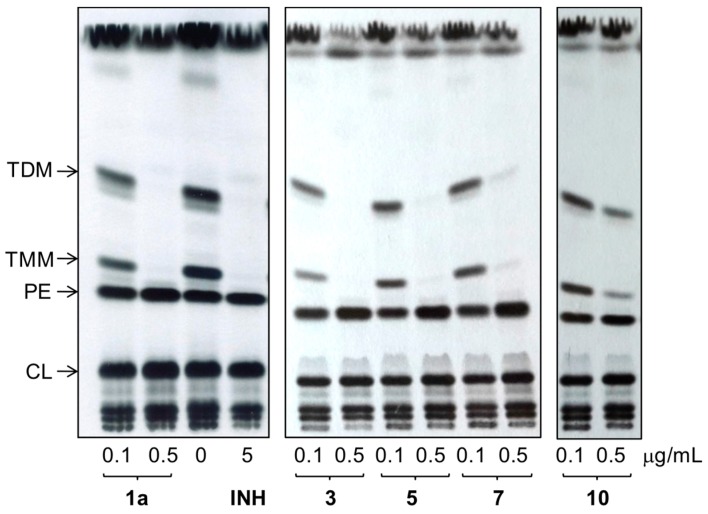
TLC analysis of lipids extracted from ^14^C acetate labeled *M.tb* H37Ra cells treated with compounds **1a**, **3**, **5**, **7** and **10**, INH and DMSO as a control. Lipids were separated in chloroform:methanol:water (20:4:0.5) and detected by autoradiography (TDM: trehalose dimycolates; TMM: trehalose monomycolates; PE: phosphatidylethanolamine; CL: cardiolipin).

In order to confirm the enoylreductase InhA as a target of synthesized hydrazones, we analyzed the effect of selected compounds with the best MIC values and cytotoxicity scores on synthesis of mycolic acids in avirulent strain *M.tb* H37Ra. Tested hydrazones, specifically **1a**, **3**, **5**, **7** and **10**, as well as INH as control InhA inhibitor, were added to *M.tb* H37Ra culture when it reached early mid-log phase of growth and, after subsequent 24 h cultivation, ^14^C acetate was added as a metabolic tracer. TLC analysis of lipid fractions extracted from harvested ^14^C labeled cells revealed that, similar to INH, all of the tested hydrazones abolish the synthesis of trehalose monomycolates and trehalose dimycolates (Figure 6).

**Figure 7:**
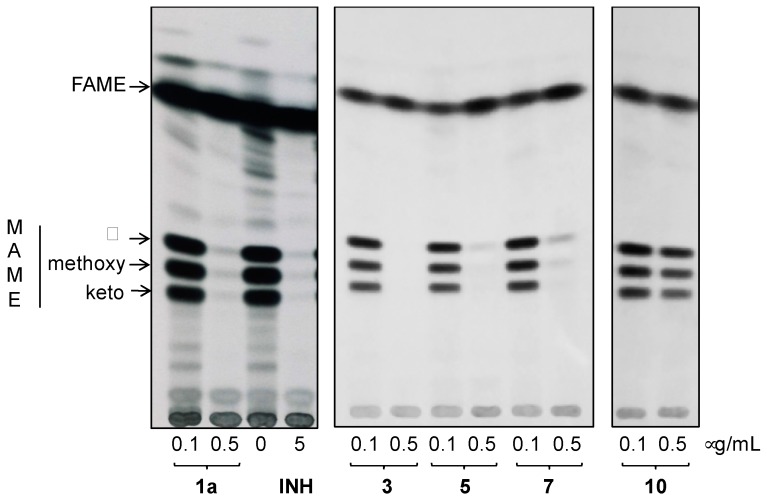
TLC analysis of methyl esters of fatty (FAME) and mycolic (MAME) acids isolated from ^14^C acetate labeled *M.tb* H37Ra cells treated with compounds **1a**, **3**, **5**, **7** and **10**, INH and DMSO as a control. Different forms of methyl esters were separated in *n*-hexane:ethyl acetate (95:5; 3×) and detected by autoradiography. (α, methoxy, and keto refer to forms of MAMEs).

Analysis of fatty/mycolic acids isolated from whole ^14^C labeled cells proved that these compounds specifically inhibit synthesis of mycolic acids (Figure 7).

**Figure 8:**
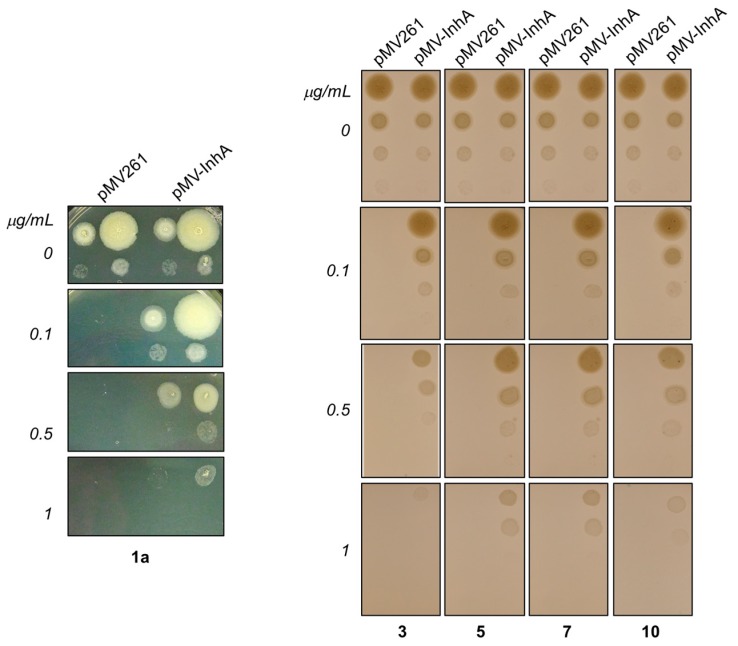
Determination of sensitivity of *M.tb* H37Ra pMV261 and *M.tb* H37Ra pMV261-InhA against **1a** (**Left** panel) and **3**, **5**, **7** and **10** (**Right** panel) by drop dilution method.

Next, we overproduced InhA protein in *M.tb* H37Ra and tested sensitivity of overproducer, as well as control strain carrying empty vector against synthesized hydrazones by drop dilution method. Clearly, this testing showed, that MICs of all of tested compounds against *M.tb* H37Ra pMV261-InhA are 5–10× higher comparing to control strain confirming InhA as molecular target of these inhibitors inside mycobacterial cells (Figure 8).

## 3. Materials and Methods

### 3.1. Material

All chemicals were obtained from Maybridge, TCI, Aldrich or Alfa Aesar, 97–99% and used without further purification. Nuclear magnetic resonance spectra (^1^H- and ^13^C-NMR) were recorded on Bruker AC 300, Avance-400 MHz and Avance-500 spectrometers with DMSO-*d*_6_ as solvent. Chemical shifts δ were expressed in parts per million (ppm) relative to TMS. Solvent residue signals were used for calibration of spectral data. Mass spectrometry (MS) data were obtained from the “Service Commun de Spectrométrie de masse” of the Plateforme Technique, Institut de Chimie de Toulouse (Toulouse, France). MS were performed using a Waters Quadrupole Time-of-flight mass spectrometer XEVO G2-S QTof. The samples were dissolved in methanol and Electrospray ionization method was used. High-resolution mass spectra (HRMS) were recorded on a ThermoFinnigan MAT 95 XL spectrometer using electrospray ionization (ESI) methods. Melting points were measured using a Kofler heating bench system Heizbank Type WME (Wagner &Munz GmbH, Munich, Germany), with measuring accuracy of ±1 °C in the range of 50–260 °C. If the melting point was higher than 260 °C or if it could not be exactly determined because of an apparent degradation, the DSC analysis was employed. The analysis was performed in a ATG-DSC 111 (Sertaram). The temperature programming was from 20 °C to 200 or 260 °C according to the sample with a constant rate of 5 °C/min under nitrogen atmosphere.

Fourier Transformed Infrared Spectroscopy (FTIR) analysis for identification was performed using KBr pellets on a Thermo Nicolet 5700 spectrometer (Thermo-Nicolet, Madison, WI, USA). The main peaks/bands were identified, especially the –C=N– that is attributed to the hydrazone. FTIR studies with the solid hydrazines as function of temperature were recorded in IN10MX Thermo Scientific FTIR microscope equipped with THMS600 (Linkam Scientific Instruments, Tadworth, Surrey, UK) heating and freezing stage.

UV-vis spectroscopy was performed using a HP (Hewlett Packard, Palo Alto, CA, USA) 8452A diode array spectrophotometer from 200 to 400 nm, with ethanol as a solvent at 20 °C and using quartz cells. The molar absorptivity was determined for the wavelength with the highest absorbance through Lambert–Beer’s law with the molar absorptivity ε in (dm^3^·mol^−1^·cm^−1^) expressed for the λ_max_ of the molecule.

### 3.2. Chemistry

#### 3.2.1. General Procedure for Phenolic Isonicotinoyl Hydrazones Synthesis

The derivatives **1a**–**d** were synthesized as previously described by us [38]. Compounds **1a**–**1d** have also a CAS number: **1a** (840-81-3); **1b** (13838-18-1); **1c** (149-17-7); **1d** (315230-80-9).

#### 3.2.2. General Procedure for Isoniazid Nitrogen-Containing Heterocycles Derivatives **2**–**11**

A mixture the solid reactants, INH (1 equivalent), the aldehyde (1 equivalent) and the catalyst (*p*-TSA, 0.5 equivalent) were placed in milling device and the reaction proceeded between 1 h–2 h, depending on the aldehyde. The Cryomill (Restch) was used for the screening of catalysts (milling started at the room temperature) at 25 Hz during 1 h. After the choice of the catalyst (*p*-TSA), all the reactions were carried out in the vibratory ball-mill Pulverisette 0 (Fritsch, Germany) equipped with a single stainless steel ball of 50 mm of diameter and 500 g, in a semi-spherical vessel of 9.5 cm of diameter. The plate vibrates with a frequency of 50 Hz and amplitude of 2.0 mm. The amounts of reactant powder were stoichiometric conditions for reactants totalizing 1 g + the amount of catalyst. The transformation was monitored by TLC. After the reaction time, the powder mixture was washed with a NaHCO_3_ solution to eliminate the catalyst and the powder was dried under vacuum. ^1^H-, ^13^C-NMR spectra and Mass Spectra for all new compounds are included in Supplementary Materials; NMR data show both conformers). NMR data reported below correspond to the major conformer.

##### (*E*)-*N*′-((1*H*-imidazol-4-yl)methylene)isonicotinohydrazide (**2**)


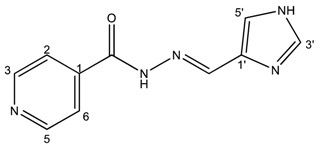

**m.p.:** 296.7 °C (dec.). ***R*_f_:** 0.1 EtOAc/MeOH (4:1 *v*/*v*).
**^1^H-NMR (300 MHz, DMSO-*d*_6_) δ ppm:** 8.15 (d, *J* = 1.4 Hz, 1H, H_5′_), 8.36 (dd, *J* = 6.6, 1.5 Hz, 2H, H_2,6_), 8.52 (s, 1H, H-C=N), 9.10 (dd, *J* = 6.6, 1.5 Hz, 2H, H_3,5_), 9.21 (d, *J* = 1.3 Hz, 1H, H_3′_), 15.67 (s, 2H, N-H).
**^13^C-NMR (75 MHz, DMSO) δ ppm:** 122.02 (1C, C_5′_), 125.10 (2C, C_2,6_), 128.25 (1C, C_1′_), 138.37 (1C, C=N), 136,80 (1C, C_3′_), 144.77 (2C, C_3,5_), 146.91 (1C, C_1_), 160.14 (1C, C=O). 126.78 (2C, C2,6), 134,36 (1C, C_5′_), 143.08 (2C, C_3,5_).
**FTIR (KBr) ν cm^−1^:** 3193.59 (N-H), 3038.08 (C-H_ar_), 1648.96 (C=O), 1626.02 (C=N-N), 1596.86 (C=Car), 1551.04 (C_ar_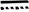 N), 1506.46 (C=N).
**UV (EtOH, 182 μM, 25 °C):** λ = 309 nm, ε = 5495.05 dm^3^·mol^−1^·cm^−1^ (very poorly soluble).
**MS (ES, TOF, MeOH) *m*/*z*:** 238.0708 [M + Na^+^]; 216.0887 [M + H^+^].
**HRMS (ES, TOF) *m*/*z*:** M + H^+^ calc. for C_10_H_10_N_5_O: 216.0885. Found: 216.0887.

##### (*E*)-*N*′-((4-methyl-1*H*-imidazol-5-yl)methylene)isonicotinohydrazide (**3**)


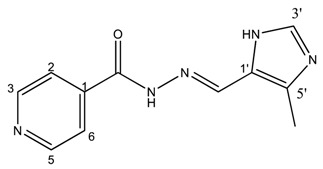

**m.p.:** 299 °C (dec.). ***R*_f_:** 0.1 EtOAc/MeOH (4:1 *v*/*v*).
**^1^H-NMR (300 MHz, DMSO-*d*_6_) δ ppm:** 2.43 (s, 3H, CH_3_), 8.42 (dd, *J* = 5.5, 1.8 Hz, 2H, H_2,6_), 8.53 (s, 1H, H-C=N), 9.11 (s, 1H, H_3′_), 9.14 (dd, *J* = 5.5, 1.8 Hz, 2H, H_3,5_), 15.98 (br, 2H, N-H).
**^13^C-NMR (75 MHz, DMSO) δ ppm:** 9.38 (1C, CH_3_), 123.60 (1C, C_1′_) 125.38 (2C, C_2,6_), 131.78 (1C, C_5′_), 138.44 (1C, C=N), 142.84 (1C, C_3′_), 144.03 (2C, C_3,5_), 147.78 (1C, C_1_), 159.52 (1C, C=O).
**FTIR (KBr) ν cm^−1^:** 3194.75 (N-H), 3097.19 (C_ar_-H), 1660.58 (C=O), 1621.45 (C=N-N), 1602.24 (C_ar_=C_ar_), 1551.49 (C_ar_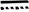 N).
**UV (EtOH, 161.58 μM, 25 °C):** λ = 316 nm, ε = 5551.73 dm^3^·mol^−1^·cm^−1^ (very poorly soluble).
**MS (ES, TOF, MeOH) *m*/*z*:** 252.0866 [M + Na^+^]; 230.1049 [M + H^+^].
**HRMS (ES, TOF) *m*/*z*:** [M + H^+^] calc. for C_11_H_12_N_5_O: 230.1042. Found: 230.1049.

##### (*E*)-*N*′-((3a,7a-dihydro-1*H*-indazol-5-yl)methylene)isonicotinohydrazide (**4**)


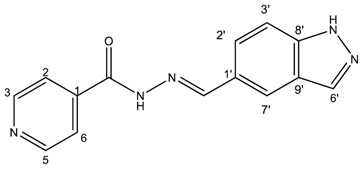

**m.p.:** 302.5 °C. ***R*_f_:** 0.45 PE/EtOAc/MeOH (5:5:3 *v*/*v*/*v*).
**^1^H-NMR (300 MHz, DMSO-*d*_6_) δ ppm:** 7.63 (d, *J* = 8.7 Hz, 1H, H_3′_), 7.84 (dd, *J* = 4.4, 1.6 Hz, 2H, H_2,6_), 7.90 (dd, *J* = 8.8, 1.5 Hz, 1H, H_2′_), 8.03–8.10 (m, 1H, H_7′_), 8.17 (t, *J* = 1.2 Hz, 1H, H_6′_), 8.57 (s, 1H, H-C=N), 8.79 (s, 2H, H_3,5_), 12.01 (s, 1H, N-H), 13.31 (s, 1H, N-H_ind_).
**^13^C-NMR (75 MHz, DMSO) δ ppm:** 111.39 (1C, C_3′_), 121.98 (2C, C_2,6_), 122.73 (1C, C_7′_), 123.35 (1C, C_9′_), 124.19 (1C, C_2′_), 127.23 (1C, C_1′_), 134.99 (1C, C_6′_), 141.07 (1C, C_1_), 141.14 (1C, C_8′_), 150.31 (1C, C=N), 150.76 (2C, C_3,5_), 161.87 (1C, C=O).
**FTIR (KBr) ν cm^−1^:** 3188.96 (N-H), 3027.37 (C_ar_-H), 1652 (C=O), 1622.47 (C=N-N), 1607.84 (C_ar_=C_ar_), 1549.40 (C_ar_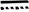 N).
**UV (EtOH, 38.37 μM, 25 °C):** λ = 234 nm, ε = 22,666.92 dm^3^·mol^−1^·cm^−1^.
**MS (ES, TOF, MeOH) *m*/*z*:** 266.1046 [M + H^+^].
**HRMS (ES, TOF) *m*/*z*:** [M + H^+^] calc. for C_14_H_12_N_5_O: 266.1042. Found: 266.1046.

##### (*E*)-*N*′-((2*H*-indazol-6-yl)methylene)isonicotinohydrazide (**5**)


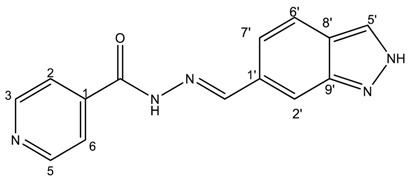

**m.p.:** 295.2 °C. ***R*_f_:** 0.45 PE/EtOAc/MeOH (5:5:3 *v*/*v*/*v*).
**^1^H-NMR (300 MHz, DMSO-*d*_6_) δ ppm:** 7.61 (dd, *J* = 8.5, 1.3 Hz, 1H, H_7′_), 7.84 (m, 4H, H_2,6,_ H_2′_, H_6′_), 8.13 (d, *J* = 1.3 Hz, 1H, H_5′_), 8.60 (s, 1H, H-C=N), 8.80 (br, 2H, H_3,5_), 12.12 (s, 1H, N-H), 13.28 (s, 1H, N-H_ind_).
**^13^C-NMR (75 MHz, DMSO) δ ppm:** 110.75 (1C, C_2′_), 119.03 (1C, C_7′_), 121.51 (2C, C_2,6_), 122.01 (1C, C_6′_), 124.39 (1C, C_8′_), 132.43 (1C, C_1′_), 134.21 (1C, C_5′_), 140.33 (1C, C_1_), 140.95 (1C, C_9′_), 150.03 (1C, C=N), 150.79 (2C, C_3,5_), 162.08 (1C, C=O).
**FTIR (KBr) ν cm^−1^:** 3193.59 (N-H), 3038.08 (C-H_ar_), 1648.96 (C=O), 1626.02 (C=N-N), 1596.86 (C=Car), 1551.04 (C_ar_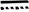 N), 1506.46 (C=N).
**UV (EtOH, 37.39 μM, 25 °C):** λ = 313 nm, ε = 25,055.1 dm^3^·mol^−1^·cm^−1^.
**MS (ES, TOF, MeOH) *m*/*z*:** 266.1047 [M + H^+^].
**HRMS (ES, TOF) *m*/*z*:** [M + H^+^] calc. for C_14_H_12_N_5_O: 266.1042. Found: 266.1047.

##### (*E*)-*N*′-((2-methyl-1*H*-indol-3-yl)methylene)isonicotinohydrazide (**6**)


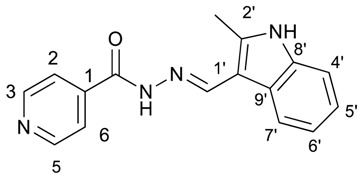

**m.p.:** 281.1 °C. ***R*_f_:** 0.55 PE/EtOAc/MeOH (5:5:3 *v*/*v*/*v*).
**^1^H-NMR (300 MHz, DMSO-*d*_6_) δ ppm:** 2.54 (s, 3H, CH_3_), 7.12 (ddt, *J* = 24.4, 9.3, 1.9, 1.9 Hz, 2H, H_5′_, H_6′_), 7.35 (tt, *J* = 2.4, 1.7, 0.9, 0.8 Hz, 1H, H_4′_), 7.85 (dd, *J* = 4.5, 1.8 Hz, 2H, H_2,6_), 8.16–8.32 (m, 1H, H_7′_), 8.71 (s, 1H, H-C=N), 8.78 (dd, *J* = 4.4, 1.6 Hz, 2H, H_3,5_), 11.55 (d, *J* = 7.4 Hz, 1H, N-H), 11.67 (s, 1H, N-H_ind_).
**^13^C-NMR (75 MHz, DMSO) δ ppm:** 11.97 (1C, CH_3_), 107.84 (1C, C_1′_), 111.34 (1C, C_4′_), 120.82 (1C, C_7′_), 121.63(1C, C_5′_), 121.87 (2C, C_2,6_), 122.34 (1C, C_6′_), 125.85 (1C, C_9′_), 136.19 (1C, C_8′_), 141.05 (1C, C_2′_), 141.56 (1C, C_1_), 146.10 (1C, C=N), 150.69 (2C, C_3,5_), 161.00 (1C, C=O).
**FTIR (KBr) ν cm^−1^:** 3385.07 (N-H), 3209.03 (N-H), 3049.08 (C_ar_-H), 1655.09 (C=O), 1626.02 (C=N-N), 1599.50 (C=C_ar_), 1550.60 (C_ar_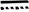 N), 1506.46 (C=N).
**UV (EtOH, 57.29 μM, 25 °C):** λ = 224 nm, ε = 21,909.58 dm^3^·mol^−1^·cm^−1^.
**MS (ES, TOF, MeOH) *m*/*z*:** 279.1246 [M + H^+^].
**HRMS (ES, TOF) *m*/*z*:** [M + H^+^] calc. for C_16_H_15_N_4_O: 279.1246. Found: 279.1246.
Compound **6** can also be found (commercial source; CAS No.: 113143-57-0).

##### (*E*)-*N*′-((1*H*-pyrrolo[2,3-b]pyridin-3-yl)methylene)isonicotinohydrazide (**7**)


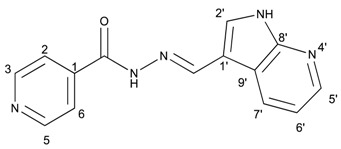

**m.p.:** 323.2 °C (dec.). ***R*_f_:** 0.34 PE/EtAc/MeOH (5:5:3 *v*/*v*).
**^1^H-NMR (300 MHz, DMSO-*d*_6_) δ ppm:** 77.24 (tt, *J* = 7.8, 4.7, 4.7 Hz, 1H, H_6′_), 7.84 (dd, *J* = 4.2, 1.7 Hz, 2H, H_2,6_), 8.03 (d, *J* = 2.3 Hz, 1H, H_2′_), 8.33 (dd, *J* = 4.7, 1.7 Hz, 1H, H_7′_), 8.58 (d, *J* = 1.6 Hz, 1H, H_5′_), 8.62 (s, 1H, H-C=N), 8.78 (dd, *J* = 4.4, 1.7 Hz, 2H, H_3,5_), 11.86 (s, 1H, N-H), 12.17 (s, 1H, N-H_ind_).
**^13^C-NMR (75 MHz, DMSO)δ ppm:** 110.85 (1C, C_1_), 117,13 (1C, C_9′_), 117.44 (1C, C_6′_), 121.95 (2C, C_2,6_), 130.56 (1C, C_5′_), 131.51 (1C, C_2′_), 141.39 (1C, C_1_), 144.56 (1C, C_7′_), 146.09 (1C, C=N), 149.84 (1C, C_8′_), 150.71 (2C, C_3,5_), 161.49 (1C, C=O).
**FTIR (KBr) ν cm^−1^:** 3454.03 (N-H), 3199.51 (N-H), 3031.09 (C_ar_-H), 1662.68 (C=O), 1611.72 (C=N-N), 1600.48 (C=C_ar_), 1551.26 (C_ar_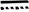 N), 1284.78 (C-N).
**UV (EtOH, 58.08 μM, 25 °C):** λ = 200 nm, ε = 18,839.92 dm^3^·mol^−1^·cm^−1^, λ = 218 nm, ε = 17,446.62 dm^3^·mol^−1^·cm^−1^, λ = 322 nm, ε = 17,193.69 dm^3^·mol^−1^·cm^−1^.
**MS (ES, TOF, MeOH) *m*/*z*:** 266.1045 [M + H^+^].
**HRMS (ES, TOF) *m*/*z*:** [M + H^+^] calc. for C_14_H_12_N_5_O: 266.1042. Found: 266.1045.

##### (*E*)-*N*′-((1*H*-indol-3-yl)methylene)isonicotinohydrazide (**8**)


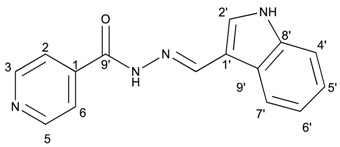

**m.p.:** 242 °C. ***R*_f_:** 0.55 PE/EtAc/MeOH (5:5:3 *v*/*v*/*v*).
**^1^H-NMR (300 MHz, DMSO-*d*_6_) δ** **ppm:** δ 7.11–7.28 (m, 2H, H_5′,6′_), 7.47 (dt, *J* = 7.9, 0.9 Hz, 1H, H_4′_), 7.85 (dd, *J* = 4.4, 1.6 Hz, 2H, H_2,6_), 7.88 (d, *J* = 2.8 Hz, 1H, H_2′_), 8.31 (dd, *J* = 6.8, 1.5 Hz, 1H, H_9′_), 8.65 (s, 1H, H-C=N), 8.78 (dd, *J* = 4.4, 1.6 Hz, 2H, H_3, 5_), 11.65 (s, 1H, N-H), 11.76 (s, 1H, N-H_ind_).
**^13^C-NMR (75 MHz, DMSO) δ** **ppm:** 111.96 (1C, C_1′_), 112.34 (1C, C_4′_), 121.00 (1C, C_5′_), 121.95 (2C, C_2,6_), 122.44 (1C, C_7′_), 123.17 (1C, C_6′_), 123.35 (1C, C_7′_), 124.77 (1C, C_9′_), 131.36 (1C, C_2′_), 137.53 (1C, C_8′_), 141.54 (1C, C_1_), 146.63 (1C, C=N), 150.69 (2C, C_3,5_), 161.33 (1C, C=O).
**FTIR (KBr) ν cm^−1^:** 3543.66 (N-H), 3395.82 (N-H), 2886.55 (C-H_ar_), 1656.52 (C=O), 1626.02 (C=N-N), 1598.83 (C=C_ar_), 1550.54 (C_ar_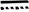 N), 1496.83 (C=N).
**UV (EtOH, 40.01 μM, 25 °C):** λ = 221 nm, ε = 22,519 dm^3^·mol^−1^·cm^−1^.
**MS (ES, TOF, MeOH) *m*/*z*:** 265.1092 [M + H^+^].
**HRMS (ES, TOF) *m*/*z*:** [M + H^+^] calc. for C_15_H_13_N_4_O: 265.1089. Found: 265.1092.
Compound **8** can also be found (commercial source; CAS No.: 10245-44-0).

##### (*E*)-*N*′-((5-bromo-1*H*-indol-3-yl)methylene)isonicotinohydrazide (**9**)


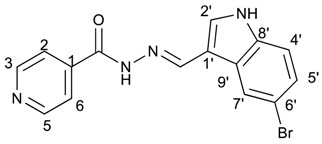

**m.p.:** 309.3 °C (dec.). ***R*_f_**: 0.61 PE/EtAc/MeOH (5:5:3 *v*/*v*).
**^1^H-NMR (300 MHz, DMSO-*d*_6_) δ ppm:** 7.35 (dd, *J* = 8.7, 2.1 Hz, 1H_5′_), 7.44 (d, *J* = 8.5 Hz, 1H, H_4′_), 7.84 (dd, *J* = 4.5, 1.9 Hz, 2H, H_2,6_), 7.95 (d, *J* = 2.7 Hz, 1H, H_9′_), 8.48 (s, 1H, H_2′_), 8.62 (s, 1H, H-C=N), 8.73–8.89 (m, 2H, H_3,5_), 11.83 (d, *J* = 6.0 Hz, 2H, N-H).
**^13^C-NMR (75 MHz, DMSO) δ ppm:** 111.59 (1C, C_1′_), 113,70 (1C, C_6′_), 114.42 (1C, C_4′_), 121.93 (2C, C_2,6_), 124.60 (1C, C_2′_), 125.71 (1C, C_5′_), 126.42 (1C, C_9′_), 132.71 (1C, C_7′_), 136.27 (1C, C_8′_), 141.38 (1C, C_1_), 146.13 (1C, C=N), 150.72 (2C, C_3,5_), 161.40 (1C, C=O).
**FTIR (KBr) ν cm^−1^:** 3127.58 (N-H_ind_), 2891.39 (C-H_ar_), 1662.69 (C=O), 1618.34 (C=N-N), 1538 (C=Car), 1552.13 (C_ar_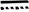 N), 1041.12 (C_ar_-Br).
**UV (EtOH, 52.65 μM, 25 °C):** λ = 201 nm, ε = 29,890 dm^3^·mol^−1^·cm^−1^, λ = 226 nm, ε = 25,981 dm^3^·mol^−1^·cm^−1^, λ = 330 nm, ε = 18,196 dm^3^·mol^−1^·cm^−1^.
**MS (ES, TOF, MeOH) *m*/*z*:** 343.0194 [M + H^+^].
**HRMS (ES, TOF) *m*/*z*:** [M + H^+^] calc. for C_15_H_12_BrN_4_O: 343.0193. Found: 343.0194.
Compound **9** can also be found (CAS No.: 113143-44-5).

##### (*E*)-*N*′-((1*H*-indol-2-yl)methylene)isonicotinohydrazide (**10**)


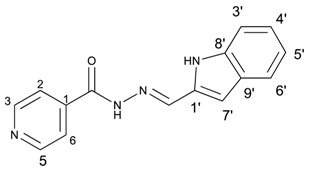

**m.p.:** 231 °C (dec.)**. *R*_f_:** 0.82 PE/EtAc/MeOH (5:5:3 *v*/*v*/*v*).
**^1^H-NMR (300 MHz, DMSO-*d*_6_) δ ppm:** 76.90 (dd, *J* = 2.1, 0.9 Hz, 1H), 7.02 (ddd, *J* = 8.0, 7.0, 1.1 Hz, 1H), 7.18 (ddd, *J* = 8.3, 7.0, 1.2 Hz, 1H), 7.47 (dq, *J* = 8.2, 0.9 Hz, 1H), 7.58 (dd, *J* = 7.9, 1.1 Hz, 1H), 7.86 (dd, *J* = 4.4, 1.6 Hz, 2H), 8.51 (s, 1H), 8.81 (dd, *J* = 4.4, 1.7 Hz, 2H), 11.65 (s, 1H), 12.05 (s, 1H).
**^13^C-NMR (75 MHz, DMSO) δ ppm:** 107.98 (1C, C_2′_), 112.57 (1C, C_3′_), 120.06 (1C, C_4′_), 121.31 (1C, C_6′_), 122.01 (2C, C_2,6_), 128.05 (1C, C_9′_), 133.28 (1C, C_1′_), 138.47 (1C, C_8′_), 142.16 (1C, C=N), 150.79 (2C, C_3, 5_), 161.84 (1C, C=O).
**FTIR (KBr) ν cm^−1^:** 3250.19 (N-H), 3032.16 (C-H_ar_), 1689.09 (C=O), 1621.50 (C=N-N), 1599.80 (C=Car), 1548.50 (C_ar_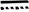 N).
**UV (EtOH, 52.65 μM, 25 °C):** λ = 206 nm, ε = 27,480 dm^3^·mol^−1^·cm^−1^, λ = 350 nm, ε = 33,825 dm^3^·mol^−1^·cm^−1^.
**MS (ES, TOF, MeOH) *m*/*z*:** 265.1090 [M + H^+^].
**HRMS (ES, TOF) *m*/*z*:** [M + H^+^] calc. for C_15_H_13_N_4_O: 265.1089. Found: 265.1090.
X-Ray structure of this compound has been recently reported [83].

##### (*E*)-*N*′-((4-nitro-1*H*-indol-3-yl)methylene)isonicotinohydrazide (**11**)


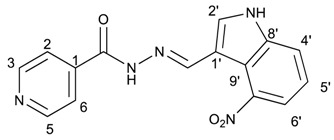

**m.p.:** 317.1 °C (dec.). ***R*_f_:** 0.70 PE/EtOAc/MeOH (5:5:3 *v*/*v*/*v*).
**^1^H-NMR (300 MHz, DMSO-*d*_6_) δ ppm:** 7.36 (t, *J* = 8.0 Hz, 1H, H_5′_), 7.84 (dd, *J* = 4.4, 1.7 Hz, 2H, H_2,6_), 7.94 (dd, *J* = 11.8, 7.9 Hz, 2H, H_4′,6′_), 8.28 (d, *J* = 2.9 Hz, 1H, H_2′_), 8.78 (dd, *J* = 4.4, 1.7 Hz, 2H, H_3,5_), 8.91 (s, 1H, H-C=N), 12.00 (s, 1H, N-H), 12.55 (s, 1H, N-H).
**^13^C-NMR (75 MHz, DMSO) δ ppm:** 110.29 (1C, C_9′_), 117.59 (1C, C_1′_), 118.94 (1C, C_4′_), 119.92 (1C, C_6′_), 121.50 (1C, C_5′_), 122.01 (2C, C_2, 6_), 131.31 (1C, C_2′_), 139.55 (1C, C_8′_), 141.22 (1C, C_1_), 141.90 (1C, C-NO_2_), 146.43 (1C, C=N), 150.66 (2C, C_3,5_), 161.56 (1C, C=O).
**FTIR (KBr) ν cm^−1^:** 3156.34 (N-H), 3137.68 (N-H), 3053.25 (C-H_ar_), 1664.06 (C=O), 1628.34 (C=N-N), 1590.99 (C=Car), 1554.10 (C_ar_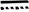 N), 1513.15 (C=N-NO_2_).
**UV (EtOH, 52.64 μM, 25 °C):** λ = 214 nm, ε = 26,971.88 dm^3^·mol^−1^·cm^−1^, λ = 331 nm, ε = 17,046.35 dm^3^·mol^−1^·cm^−1^.
**MS (ES, TOF, MeOH) *m*/*z*:** 332.0760 [M + Na^+^]; 310.0940 [M + H^+^].
**HRMS (ES, TOF) *m*/*z*:** [M + H^+^] calc. for C_15_H_12_N_5_O_3_: 310.0937. Found: 310.0940.

### 3.3. Physicochemical Studies: Hydrolytic Stability and pK_a_ Determination

Physicochemical studies: hydrolytic stability and p*K*_a_ determination:

UV spectra were recorded with HP8453 (Agilent) temperature controlled spectrophotometer.

The pHs of the solutions were measured at room temperature (temperature probe) with a combined pH electrode with Seven Multi (Mettler Toledo) pHmeter.

#### 3.3.1. Hydrolytic Stability

In the case of compounds **1a**, **1b**, **1c** and **1d**, a small quantity (respectively, 1.45 mg, 1.63 mg, 1.87 mg, and 1.79 mg) of each product was weighted and dissolved in 1 mL of EtOH. The aliquots were then placed in 100 mL standard flask with a concentration of 28% EtOH/H_2_O. Final concentrations of compounds were respectively **1a**: 6.0 × 10^−5^ mol/L, **1b**: 6.3 × 10^−5^ mol/L, **1c**: 6.0 × 10^−5^ mol/L and **1d**: 5.9 × 10^−5^ mol/L.

In the case of compounds **5** and **7**, a small quantity (respectively, 1.65 mg and 1.20 mg) of each product was weighted and dissolved in 4 mL of EtOH. A fraction of this solution (respectively 1.30 mL and 1.36 mL) were placed in 50 mL standard flask and mixed with PIPES buffer (50 mM) with a final concentration of 5% EtOH/PIPES.

The solution was stirred at room temperature; pH values and λ max of absorbance of the related compounds were measured for longer than 15 h.

#### 3.3.2. p*K*_a_ Determination

A small quantity (1–2 mg) of each product was weighted and dissolved in a 1 mL of EtOH. The aliquots were then placed in 100 mL standard flask with a final concentration of 28% EtOH/H_2_O. The ionic strength was fixed at 0.1 M with potassium chloride. The pH was adjusted to the required value by adding concentrated 0.1 M KOH or 0.1 M HCl. The solution was stirred at room temperature. The absorbance at three different wavelengths was measured. Absorbance vs. pH curves were plotted and the p*K*_a_ values were evaluated graphically by geometric method [84].

#### 3.3.3. DMSO stability of Compound **1d**

The stability of compound **1a** in DMSO was assessed at 25 °C. To this purpose, the compound was dissolved in DMSO, at a final concentration of 10 mM, and incubated for 16 h at 25 °C. The compound was then diluted in methanol, and analysed. The mass spectra were recorded in negative ESI resolution mode with a Thermo LTQ-XL mass spectrometer, and compared with those of compound **1a** and of INH freshly dissolved in DMSO (10 mM) and diluted in methanol.

### 3.4. Biological Assays

#### 3.4.1. Inhibition Kinetics in the Presence of InhA

Inhibition kinetic was performed as described [81,85].

#### 3.4.2. MIC Determination in *M.tb*

H37Rv strain was used as the reference strain. *M.tb* H37Rv and IC2 clinical isolate [31] were grown at 37 °C in Middlebrook 7H9 broth (Difco), supplemented with 0.05% Tween 80, or on solid Middlebrook 7H11 medium (Difco) supplemented with oleic acid-albumin-dextrose-catalase (OADC). MICs for the compounds were determined by means of the micro-broth dilution method. Dilutions of *M.tb* wild-type or mutant strain (about 10^5^–10^6^ cfu/mL) were streaked onto 7H11 solid medium containing a range of drug concentrations (0.125 µg/mL to 40 µg/mL). Plates were incubated at 37 °C for about 21 days and the growth was visually evaluated. The lowest drug dilution at which visible growth failed to occur was taken as the MIC value. Results were expressed as the average of at least three independent determinations.

#### 3.4.3. Determination of LC_50_

Human primary fibroblast (MRC5 ATCC CCL171) were seeded at 5 k/well in a corning Cell bind 96-well plate and treated with growing concentration of compounds. Seventy-two hours post-treatment, cells were directly stained with Hoechst 33342 and imaged under a Cellomics Array scan HCS microscope using the cell cycle algorithm. Total number of cells were assessed in each condition and normalized over untreated wells. LC_50_ were determined as concentration inducing 50% reduction in cell number.

#### 3.4.4. Analysis of the Effect of Hydrazones on Lipids and Mycolic Acids of *M.tb* H37Ra

The modes of actions of selected hydrazones were analyzed by metabolic labeling of *M.tb* H37Ra strain with ^14^C acetate as already described [34]. Briefly, *M.tb* H37Ra culture was grown in Middlebrook 7H9 broth (Difco) supplemented with albumin-dextrose-catalase (Difco) and 0.05% Tween 80 (MP Biomedicals) at 37 °C till OD_600_ reached 0.24. The culture was then divided into 20 mL aliquots and tested compounds dissolved in DMSO were added in 0.41 μM (0.1 μg/mL) and 2.05 μM (0.5 μg/mL) final concentrations for **1a**, 0.44 μM (0.1 μg/mL) and 2.2 μM (0.5 μg/mL) for **3**, 0.38 μM (0.1 μg/mL) and 1.9 μM (0.5 μg/mL) for **5**, 0.38 μM (0.1 μg/mL) and 1.9 μM (0.5 μg/mL) for **7**, 0.38 μM (0.1 μg/mL) and 1.9 μM (0.5 μg/mL) for **10** and 36.5 μM (5 μg/mL) for INH. The final concentration of DMSO was kept at 1% in each experiment. ^14^C acetate (specific activity 106 mCi/mmol, ARC) in the final concentration of 0.5 μCi/mL was added to each of the cultures after 24 h of cultivation with shaking (120 rpm) and the cells were cultivated for next 24 h.

Lipids were extracted from whole cells harvested from 10 mL culture aliquots as described earlier [34,86], dissolved in chloroform:methanol (2:1)—350 μL per 1 unit of OD_600_ of harvested cells. Five microliters were loaded on thin-layer chromatography (TLC) silica gel plates F_254_ (Merck) and the lipids were separated in chloroform:methanol:water (20:4:0.5) and detected by autoradiography. Fatty acid methyl esters (FAME) and mycolic acids methyl esters (MAME) were prepared from whole cells harvested from 10 mL culture aliquots as previously described [34,87]. Dried extracts were dissolved in chloroform:methanol (2:1) and loaded on TLC plates as described for lipid extracts. Different forms of methyl esters were separated by running three times in n-hexane: ethyl acetate (95:5) and detected by autoradiography.

#### 3.4.5. Determination of Sensitivity of *M.tb* H37Ra Strain Overproducing InhA to Hydrazones

InhA protein was overproduced in *M.tb* H37Ra using pMV261-InhA construct as already described [88]. Sensitivity of InhA overproducing strain, as well as control strain carrying empty vector to compounds **1a**, **3**, **5**, **7** and **10** was analysed by drop dilution methods. Both cultures grown in 7H9 broth supplemented with albumin-dextrose-catalase and 0.05% Tween 80 were adjusted to OD_600_ 0.5 and 4 µL aliquots of 10^0^, 10^−1^, 10^−2^ and 10^−3^ dilutions were dropped on 7H11 agar supplemented with oleic acid-albumin-dextrose-catalase and incubated 25 days at 37 °C.

## 4. Conclusions

A series of hydrazones were synthesized through mechanochemistry and evaluated for their ability to inhibit *M.tb* H37Rv strain growth, the most active being **1a** and **7**. Imidazole derivative **5** and indazole one **10** are also active against H37Rv with their nitro substituent. Compounds **1a** and **1b** were the most effective against both *M.tb* H37Rv strain and drug-resistant IC2 isolate.

The mechanism of anti-mycobacterial activities of selected hydrazones with the best scores regarding the MICs and toxicities was confirmed to be through the cessation of mycolic acid synthesis due to InhA inhibition inside the mycobacterial cell.

## Figures and Tables

**Table 1:** Hydrazones **2**–**11** produced mechanochemically by reacting INH and imidazolic, indazolic or indolic aldehydes. The reaction was catalyzed by *p*-TSA.

Aldehydes	Heterocyclic Hydrazones (6) Derived From Isoniazid	
Imidazole derivatives	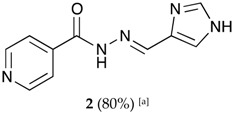	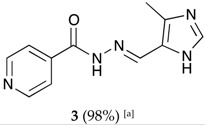
Indazole derivatives	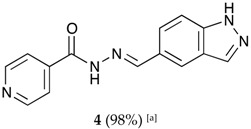	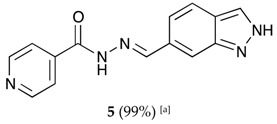
Indole derivatives	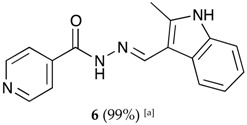	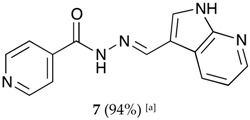
	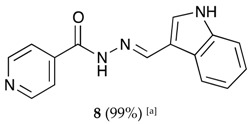	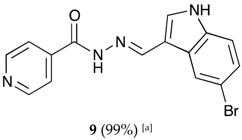
	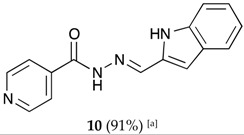	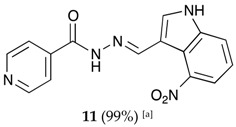

^[a]^ Yields after washing with NaHCO_3_ aqueous solution to eliminate *p-*TSA. According to TLC, ^1^H-NMR and MS, the conversions were quantitative.

**Table 2:**
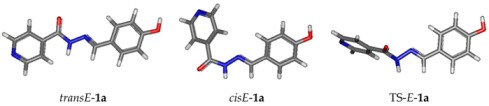
Geometries and energies of minima and transition state for *cis* and *trans E*-isomers of **1a** obtained at B3LYP/6-31+G(d,p) level in the gas phase and using the DMSO polarizable continuum model (SMD).

**In the Gas Phase**	***E* (ua)**	***G* (ua)**	**Δ*G* (kcal/mol)**	**%**
*transE*_**1a**	−816.730569	−816.553707	1.47	8
*cisE*_**1a**	−816.733307	−816.55605	0	92
**In DMSO**	***E* (ua)**	***G* (ua)**	**Δ*G* (kcal/mol)**	**%**
*transE*_**1a**	−816.763427	−816.585816	−1.68	94
*cisE*_**1a**	−816.761415	−816.583145	0	6
TS_**1a**	−816.765271	−816.557724	17.63	-

**Table 3:** Geometries, Gibbs free energies and Boltzmann distribution of the four major conformers of **5** at the B3LYP/6-31+G(d,p) in the gas phase and in the DMSO continuum solvent model (SMD).

Isomer	Geometry (*In Gas Phase*)	G (*In Gas Phase*)	%	G (*In DMSO*)	%
*transE*_5_1	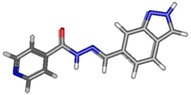	−888.90736	0.34	−888.94471	28.22
*transE*_5_2	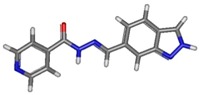	−888.909926	5.09	−888.94528	51.50
*cisE*_5_1	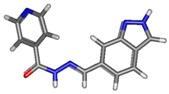	−888.910465	9.0	−888.941968	1.54
*cisE*_5_2	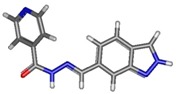	−888.912591	85.6	−888.944326	18.72

**Table 4:** Stability study of hydrazones.

Compound	Medium	Conc. (mol/L)	pH	Time	Stability
**1a**	28% EtOH/H_2_O	6.0 × 10^−5^	6.1	22 h	stable
**1b**		6.3 × 10^−5^	6.5	22 h	stable
**1c**		6.9 × 10^−5^	6.3	15 h	stable
**1d**		5.9 × 10^−5^	6.2	21 h	stable
**5**	5% EtOH/PIPES buffer (50 mM)	4.1 × 10^−5^	6.8	20 h	3% Abs. reduction
**7**		3.1 × 10^−5^	6.8	20 h	stable
				7 days	5% Abs. reduction

**Table 5:** p*K*_a_ values determined for the isonicotinoylhydrazones.

Compound	1a	1b	1c	1d	5	7
p*K*_a1_	nd ^a^	nd ^a^	3.4	3.0	3.4	3.6
p*K*_a2_	9.2 ± 0.1	7.4 ± 0.1	9.1 ± 0.1	9.5 ± 0.1	10.4 ± 0.1	11
p*K*_a3_	-	8.9 ± 0.1	-	>11	-	-

^a^ nd for not determined.

**Table 6:** Enzyme inhibition values for the INH derivatives. Results are expressed as a percentage of InhA inhibition.

Compound	% Inhibition at 50 μM (Inhibitor)	Compound	% Inhibition at 50 μM (Inhibitor)
**1a**	45	**5**	19
**1b**	54	**6**	43
**1c**	48	**7**	39
**1d**	64	**8**	42
**2**	54	**9**	32
**3**	3	**10**	79
**4**	33	**11**	not soluble
**TCL**	>99		

**Table 7:** Phenolic isonicotinoyl hydrazones tested as inhibitory agents against *M.tb* growth (H37Rv strain).

Compound	MW (g/mol)	MIC (μg/mL)/(μM)	LogP	Cpd	MW (g/mol)	MIC (μg/mL)/(μM)	LogP
**1a**	241.25	0.0125/0.05	1.64	**1c**	271.27	0.125/0.46	1.51
**1b**	257.24	0.125/0.49	1.25	**1d**	301.30	0.125/0.41	1.38
**INH**	137.14	0.025/0.18	−0.64				

**Table 8:** MICs of Isoniazid-Nitrogen heterocyclic hydrazones against *M.tb* H37Rv.

Compound	MW (g/mol)	MIC (μg/mL)/(μM)	LogP	Cpd	MW (g/mol)	MIC (μg/mL)/(μM)	LogP
**2**	215.21	0.03/0.14	−1.00	**7**	265.27	0.015/0.056	0.24
**3**	229.24	0.03/0.13	−1.37	**8**	264.28	0.06/0.23	0.86
**4**	265.27	0.06/0.23	1.38	**9**	343.18	0.125/0.36	1.69
**5**	265.27	0.03/0.11	−0.52	**10**	264.28	0.06/0.23	0.52
**6**	278.31	0.25/0.90	0.49	**11**	309.28	0.25/0.81	1.39
**INH**	137.14	0.05/0.36	−0.64				

**Table 9:** MIC of isoniazid derivatives against *M.tb* MDR isolate IC2.

Compound	MIC (μg/mL)/(μM)	
	H37Rv	IC2
**1a**	0.0125/0.05	2.5/10.36
**1b**	0.125/0.49	1/3.89
**1c**	0.125/0.46	>2.5/>9.22
**1d**	0.125/0.41	>2.5/>8.30
**2**	0.03/0.14	5/23.2
**3**	0.03/0.13	5–10/21.8–43.6
**4**	0.06/0.23	>10
**5**	0.03/0.11	>10
**6**	0.25/0.90	>10
**7**	0.015/0.056	>10
**8**	0.06/0.23	>10
**9**	0.125/0.36	>10
**10**	0.06/0.23	>10
**11**	0.25/0.81	5–10/18.9–37.8
**INH**	0.025/0.18	>2/>14.58

**Table 10:** Cytotoxicity (LD_50_) and selectivity index (SI) for the most active hydrazones against H_37_Rv *M.tb*.

Compound	LD_50_ (μM)	SI	Compound	LD_50_ (μM)	SI
**1a**	>80	>1600	**5**	>80	>727
**1b**	36.3	74	**6**	129	143
**1c**	>80	>173	**7**	>80	>1429
**1d**	>80	>195	**8**	>80	>364
**2**	>80	>571	**9**	>80	>222
**3**	>80	>615	**10**	71.4	310
**4**	>80	>364	**11**	156	193
**INH**	-	-			

## Supplementary Materials

Supplementary Materials are available online.
