# Supplementary material for: Mechanochemical Synthesis and Biological Evaluation of Novel Isoniazid Derivatives with Potent Antitubercular Activity
Source: Molecules. 2017 Sep 1;22(9):1457. doi: 10.3390/molecules22091457 (PMC6151834; doi:10.3390/molecules22091457)
Supplement: Supplementary file 1 [file molecules-22-01457-s001.pdf]

Supplementary information

Mechanochemical synthesis and biological evaluation of novel isoniazid derivatives with potent antitubercular activity

Paulo F. M. Oliveira,[†,‡,§,#] *Brigitte Guidetti,*[‡,§] *Alain Chamayou,*[†] *Christiane André-Barrès,*[‡,§] *Jan Madacki,*[‖] *Jana Korduláková,*[‖,*] *Giogia Mori,*[⊥] *Beatrice Silvia Orena,*[⊥] *Laurent Roberto Chiarelli,*[⊥] *Maria Rosalia Pasca,*[⊥,*] *Christian Lherbet,*[‡] *Chantal Carayon,*[‡,§] *Stéphane Massou,*[⁋] *Michel Baron,*[†] *Michel Baltas,*[‡,§,*]

[†] Université de Toulouse, Mines-Albi, CNRS UMR 5302, Centre RAPSODEE, Campus Jarlard, 81013 Albi Cedex 09, France.

[‡] Université de Toulouse, UPS, CNRS UMR 5068, LSPCMIB, 118 Route de Narbonne, 31062, Toulouse Cedex 09, France.

[§] CNRS; Laboratoire de Synthèse et Physico-Chimie de Molécules d'Intérêt Biologique, LSPCMIB, UMR-5068; 118 Route de Narbonne, F-31062 Toulouse cedex 9, France.

[‖] Department of Biochemistry, Comenius University in Bratislava, Faculty of Natural Sciences, Mlynská dolina, Ilkovičova 6, 842 15, Bratislava, Slovakia

[⊥] Department of Biology and Biotechnology "LazzaroSpallanzani", University of Pavia; via Ferrata 1, 27100 Pavia, Italy.

Table S1: Geometries and energies (in atomic units) of minima for *cis* and *transE*-isomers of **1a** obtained at the HF STO-3G level in the gas phase.

Table S2: Calculated and experimental ^{1}H and ^{13}C NMR chemical shifts in the DMSO, for *cis* and *trans E* isomers of **1a** using the numbering of the modelling.

Figure S1: ^{1}H NMR spectra at increasing temperature (298 to 388K and then return to 298K) for compound **1a**

Table S3: Determination of activation energy ($\Delta G^{\neq}$) from coalescence temperatures T_c and rate exchange k_{exch} at different chemical shift for the **1a** isomers.

Table S4: Geometries and energies of minima for *cis* and *transE*-isomers of **5** obtained at the HF STO-3G level in the gas phase.

Table S5: Calculated and experimental ^{1}H and ^{13}C NMR chemical shifts in the DMSO, for *cis* and *trans E* isomers of **5** using the numbering of the modelling.

Figure S2: ^{1}H NMR spectra at increasing temperature (298 to 388K and then return to 298K) for **5**

Table S6: Determination of activation energy ($\Delta G^{\neq}$) from coalescence temperatures T_c and rate exchange k_{exch} at different chemical shift for the isomers of **5**.

Figure S3: Stability of compound **1d** in DMSO; A) From top to bottom: full-MS of compound **1d** upon overnight incubation in DMSO; compound **1d** freshly dissolved; INH dissolved in DMSO. B) Fragmentation pattern of the compounds (MS-MS (ESI)@cid30.0).

Figure S4: a. UV-*vis* spectra obtained for compound **1c** as function of pH variation. **b**. Plots of absorbance for **3** wavelength values (λ/nm) as function of pH.

Figure S5: a. UV-*vis* spectra obtained for compound **1a** as function of pH variation. **b**. Plots of absorbance for **3** wavelength values (λ/nm) as function of pH.

Figure S6: a. UV-*vis* spectra obtained for compound **1b** as function of pH variation. **b**. Plots of absorbance for **3** wavelength values (λ/nm) as function of pH.

Figure S7: a. UV-*vis* spectra obtained for compound **5** as function of pH variation. **b**. Plots of absorbance for **4** wavelength values (λ/nm) as function of pH.

Figure S8: a. UV-*vis* spectra obtained for compound **7** as function of pH variation. **b**. Plots of absorbance for **4** wavelength values (λ/nm) as function of pH.

Mass HRMS and NMR data of compounds **2-11** synthetized

	geometry	E(HF)	ΔE(ua)	ΔE(kcal)	%
***transE*_1a**	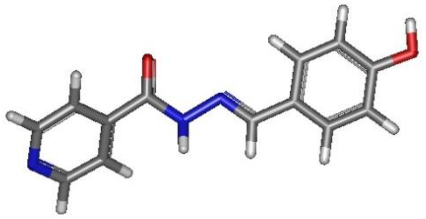	-801,482133	0,00219939	1,38016121	8,87
***cisE*_1a**	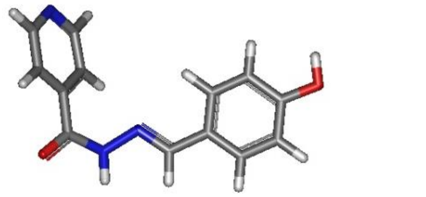	-801,484332	0	0	91,12
***transZ*_1a**	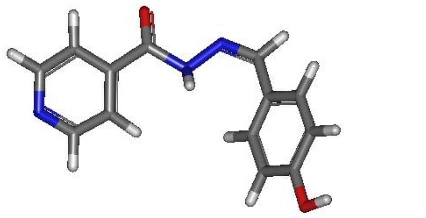	-801,475096	0,0092361	5,79583747	0,0005
***cisZ*_1a**	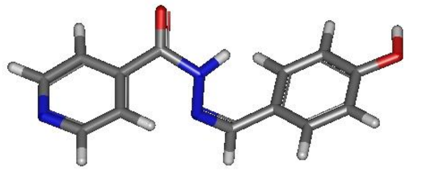	-801,475228	0,00910358	5,71267852	0,0006

Table S1: Geometries and energies (in atomic units) of minima for *cis* and *transE*-isomers of **1a** obtained at the HF STO-3G level in the gas phase.

Table S2: Calculated and experimental ^{1}H and ^{13}C NMR chemical shifts in the DMSO, for *cis* and *trans E* isomers of **1a** using the numbering of the modelling.

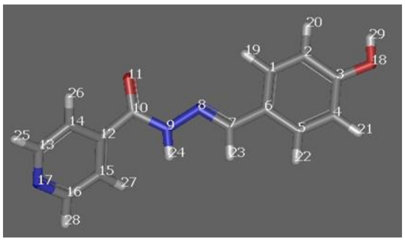

***transE*-1a**

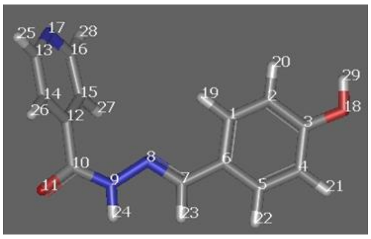

***cisE*-1a**

nuclei	calculated δ(ppm)		experimental δ(ppm)	
**C**	***trans***	***cis***	***majo***	***mino***
1, 5	129.35	128.94	128.88	128.41
2, 4	113.60	113.48	115.57	115.47
3	158.28	157.87	159.5	159.12
6	125.16	125.14	124.8	124.79
7	146.08	143.45	149.16	144.82
10	160.15	166.16	161.08	167.18
12	140.67	141.31	140.49	141.68
14, 15	119.86	122.50	121.27	122.92
13, 16	150.06	148.92	150.07	149.16

H	***trans***	***cis***	***majo***	***mino***
19.22	7.73	7.66	7.81	7.6
20.21	6.87	6.78	6.88	6.8
23	7.82	7.44	8.37	8.01
25.28	8.72	8.74	8.78	8.72
26.27	7.57	7.55	7.6	7.35

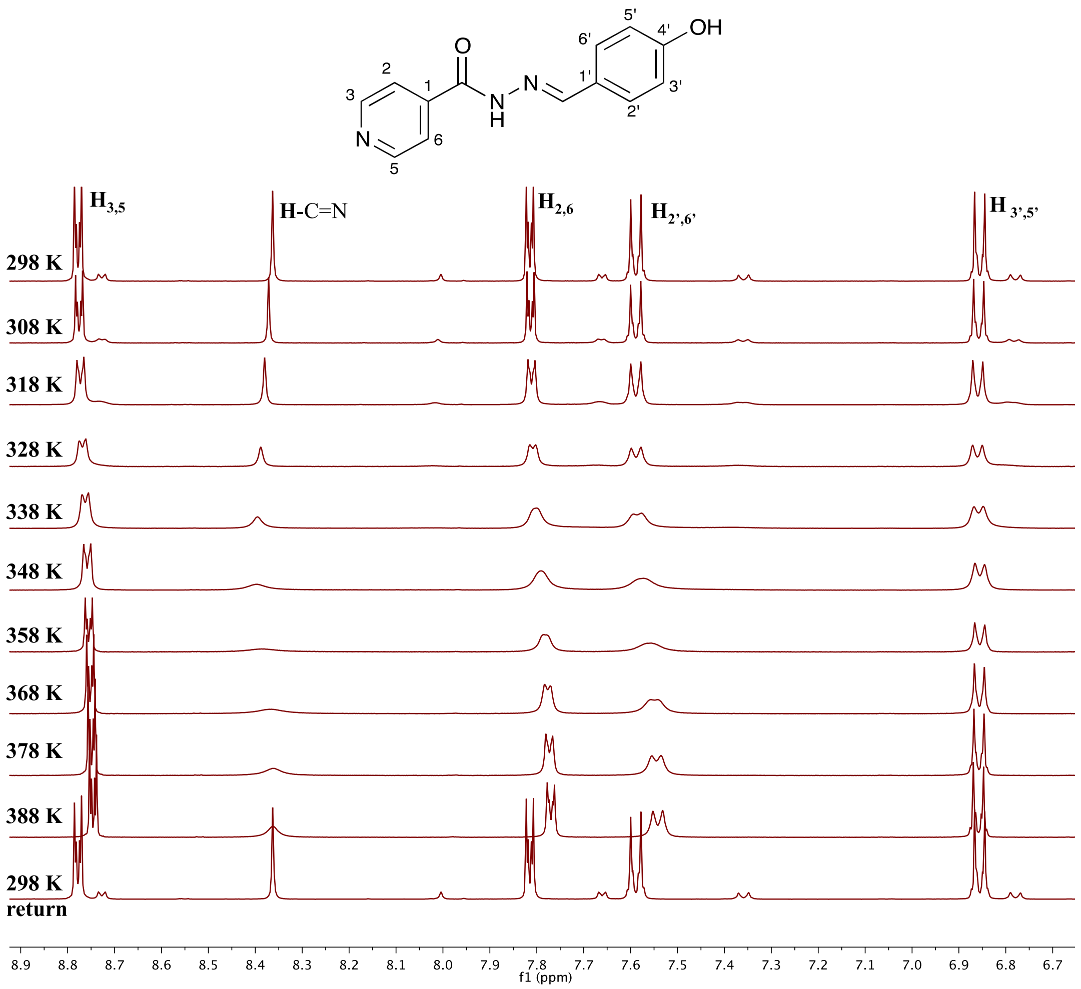

Figure S1: ^{1}H NMR spectra at increasing temperature (298 to 388K and then return to 298K) of compound **1a**

Table S3: Determination of activation energy ($\Delta G^{\neq}$) from coalescence temperatures $\boldsymbol{T_c}$ and rate exchange $\boldsymbol{k_{exch}}$ at different chemical shift for the **1a** isomers.

**Nuclei**	$\boldsymbol{T_c}$ **(K)**	$\boldsymbol{k_{exch}}$ **(Hz)**	$\boldsymbol{\Delta G^{\neq}}$ **(kcal/mol)**
$H_{3,5}$	338	33.97	17.52
H-C=N	368	239.1	17.71
$H_{2,6}$	352	102.6	17.50
$H_{2',6'}$	362	153.2	17.73
$H_{3',5'}$	342	51.28	17.45

Mean value : $\Delta G^{\neq}$= 17.58 kcal.mol^{-1}

	geometry	E(HF)	ΔE (kcal/mol)	%
***transE*_5_1**	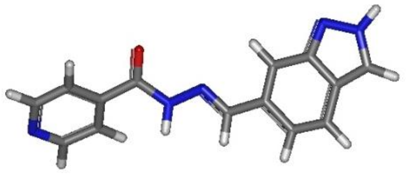	-872,506656	2,57	0,94
***transE*_5_2**	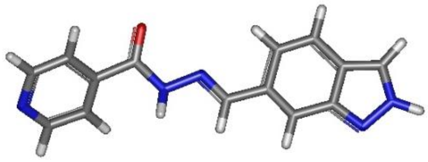	-872,508008	1,72	3,94
***transZ*_5_1**	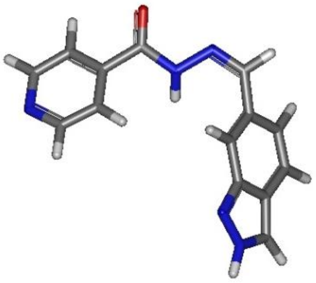	-872,501501	5,81	0,004
***transZ*_5_2**	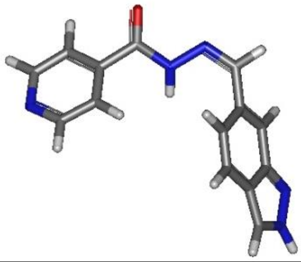	-872,500381	6,51	0,001
***cisE*_5_1**	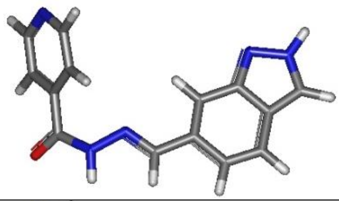	-872,509651	0,69	22,49
***cisE*_5_2**	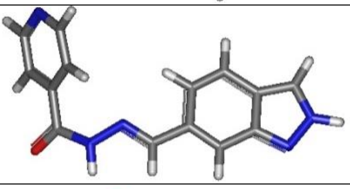	-872,510757	0	72,54
***cisZ*_5_1**	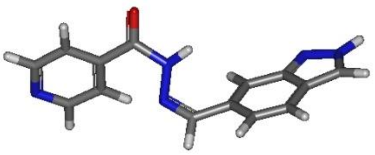	-872,504306	4,05	0,078
***cisZ*_5_2**	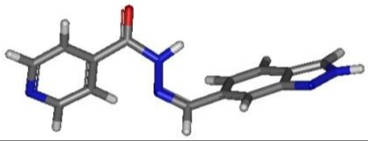	-872,49888	7,45	0,0002

Table S4: Geometries and energies of minima for *cis* and *transE*-isomers of **5** obtained at the HF STO-3G level in the gas phase.

Table S5: Calculated and experimental ^{1}H and ^{13}C NMR chemical shifts in the DMSO, for *cis* and *trans E* isomers of **5** using the numbering of the modelling.

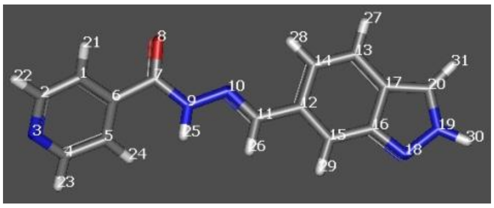

**C**	***transE***	***cisE***	*majo*	*mino*
1	119.85	122.39	121.55	122.01
2	150.15	148.99	150.77	
4	150.15	148.99	150.77	
5	119.85	122.39	121.55	122.01
6	140.43	141.46	140.35	140.35
7	160.20	166.75	162.1	168.4
11	147.40	144.73	150.03	149.99
12	131.44	131.72	132.43	132.27
13	121.33	121.29	122.01	122.01
14	119.16	117.15	119.02	118.57
15	118.96	120.81	119.02	118.57
16	147.50	147.45	140.97	140.97
17	121.80	121.36	124.36	124.2
20	121.76	121.73	122.01	122.01

**H**	***transE***	***cisE***	***majo***	***mino***
21	7.63	7.59	7.86	7.68
22	8.76	8.55	8.80	8.8
23	8.76	8.55	8.80	8.8
24	7.63	7.59	7.86	7.68
26	8.08	7.82	8.60	8.23
27	7.91	7.76	7.82	7.75
28	7.71	7.49	7.61	7.3
29	8.15	7.81	7.84	7.66
31	8.08	8.02	8.13	8.08

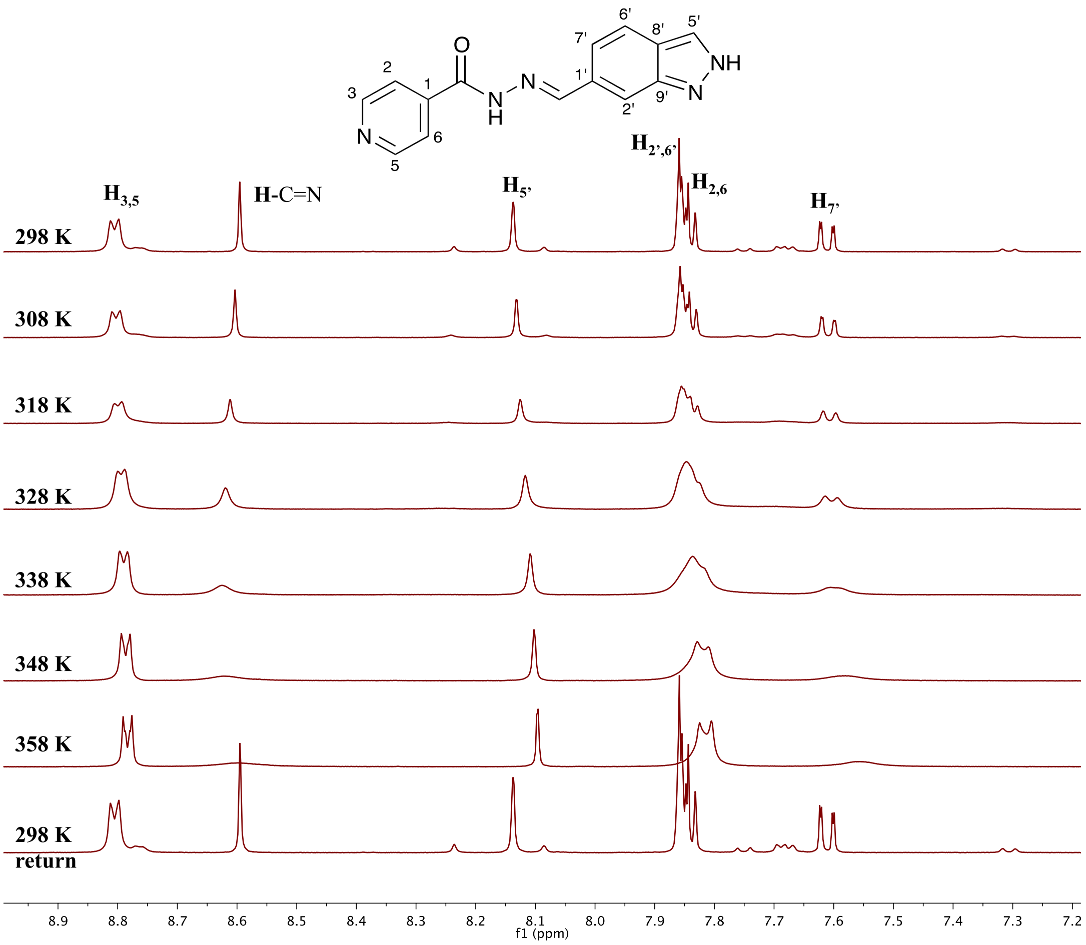

Figure 2-SI: ^{1}H NMR spectra at increasing temperature (298 to 388K and then return to 298K) for **5**

Table S6: Determination of activation energy ($\Delta G^{\neq}$) from coalescence temperatures $\boldsymbol{T_c}$ and rate exchange $\boldsymbol{k_{exch}}$ at different chemical shift for the isomers of **5**.

Nuclei	T_c (K)	k_{exch} (Hz)	$\Delta G^{\neq}$ (kcal/mol)
$H_{3,5}$	328	26.64	17.14
H-C=N	358	239.76	17.20
$H_{5'}$	338	35.30	17.49
$H_{2',6'}$/$H_{2,6}$	348	108.56	17.25
$H_{7'}$	358	199.80	17.33

Mean value: $\Delta G^{\neq}$ =17.28 kcal/mol

Figure S3: Stability of compound **1d** in DMSO; A) From top to bottom: full-MS of compound **1d** upon overnight incubation in DMSO; compound **1d** freshly dissolved; INH dissolved in DMSO. B) Fragmentation pattern of the compounds (MS-MS (ESI)@cid30.0).

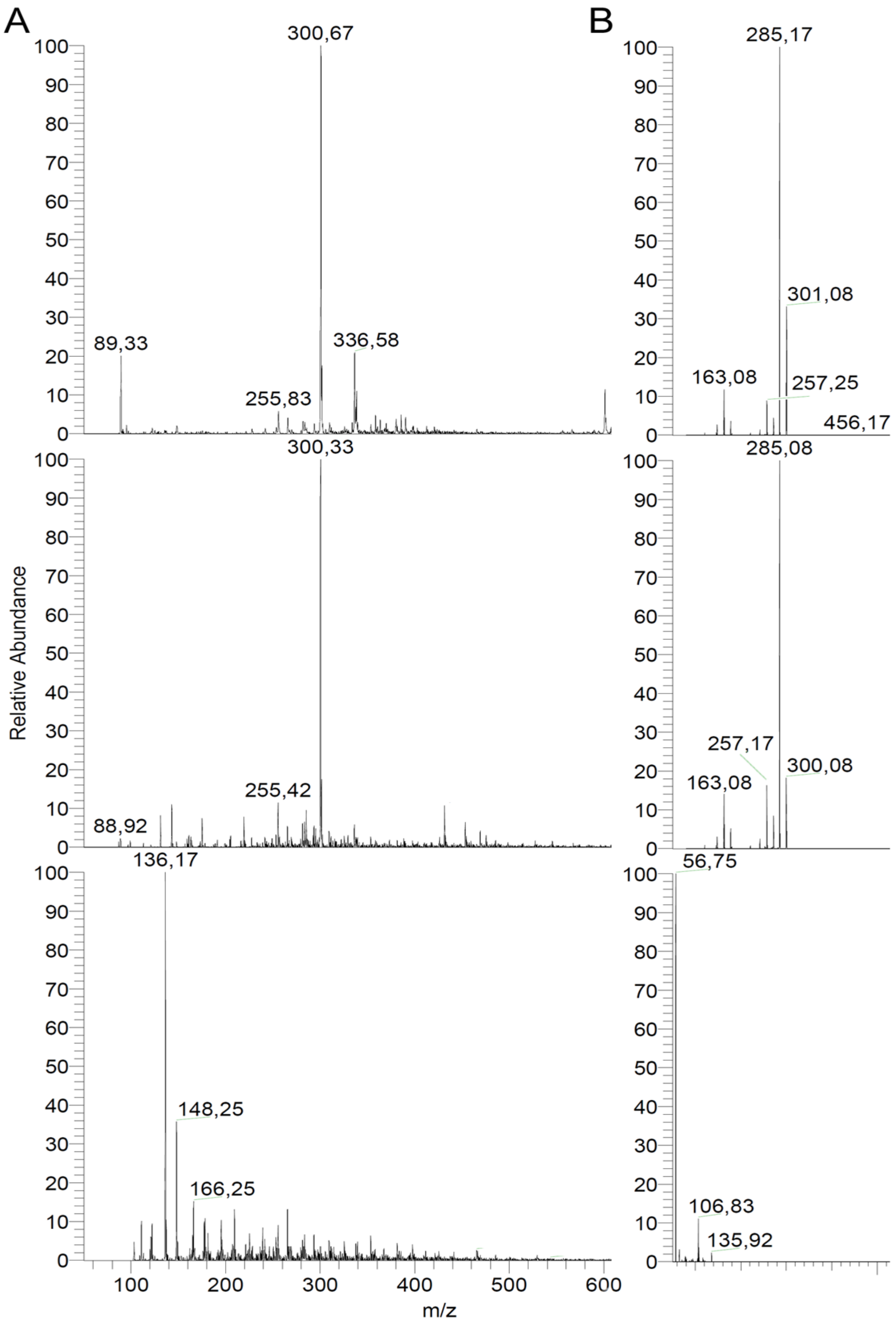

Figure S4: a. UV-*vis* spectra obtained for compound **1c** as function of pH variation.

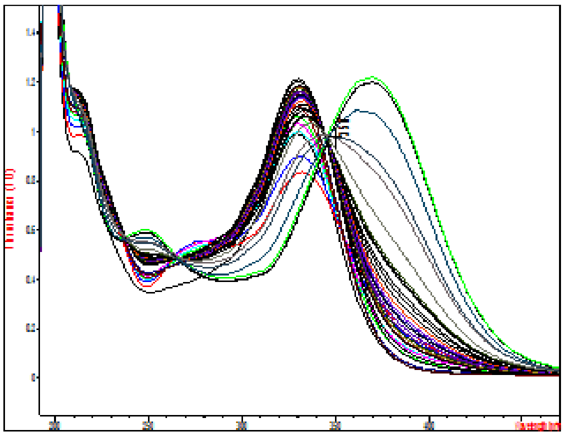

Figure S4: b. Plots of absorbance for 3 wavelength values (λ/nm) as function of pH.

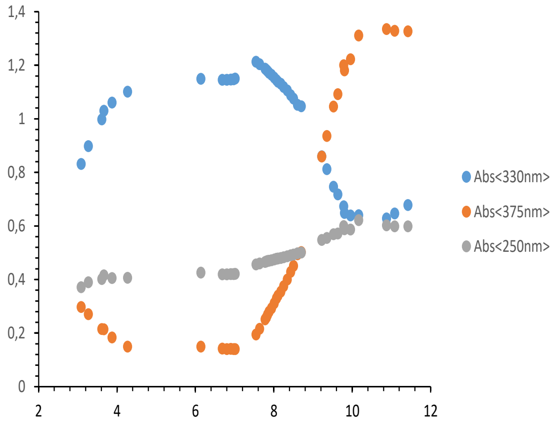

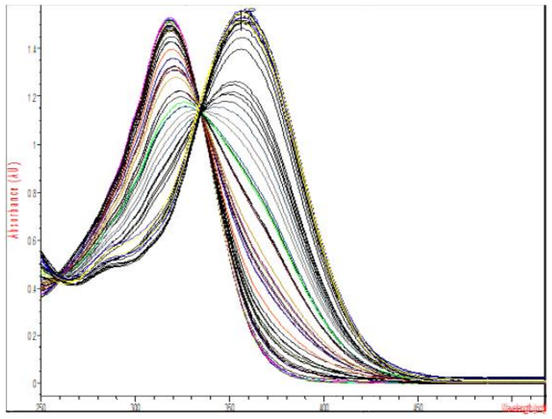

Figure S5: a. UV-*vis* spectra obtained for compound **1a** as function of pH variation.

Figure S5: b. Plots of absorbance for 3 wavelength values (λ/nm) as function of pH.

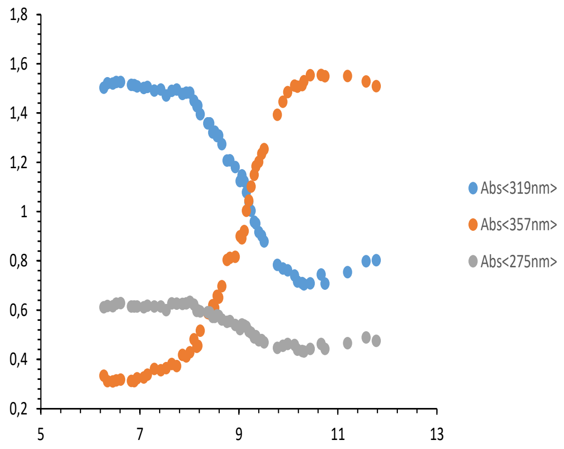

Figure S6: a. UV-*vis* spectra obtained for compound **1b** as function of pH variation.

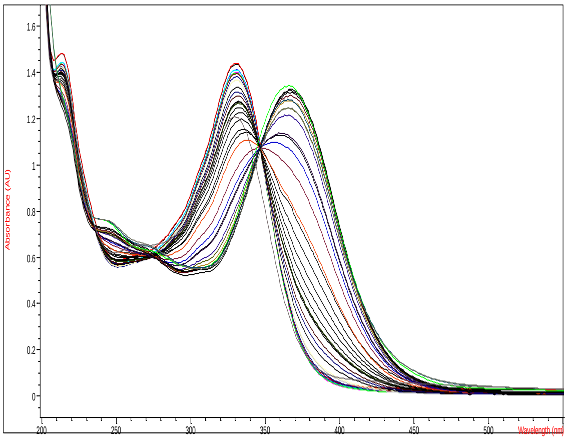

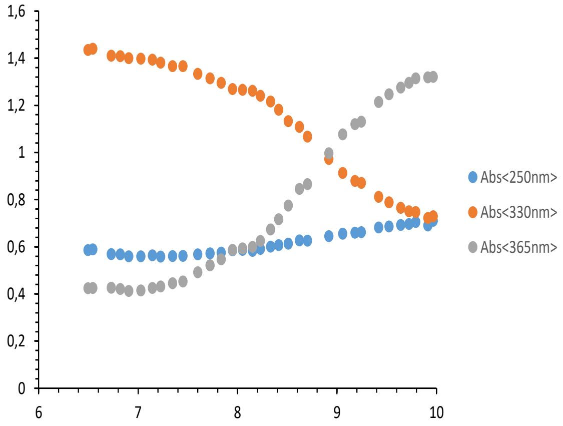

Figure S6: b. Plots of absorbance for 3 wavelength values (λ/nm) as function of pH

Figure S7: a. UV-*vis* spectra obtained for compound **5** as function of pH variation.

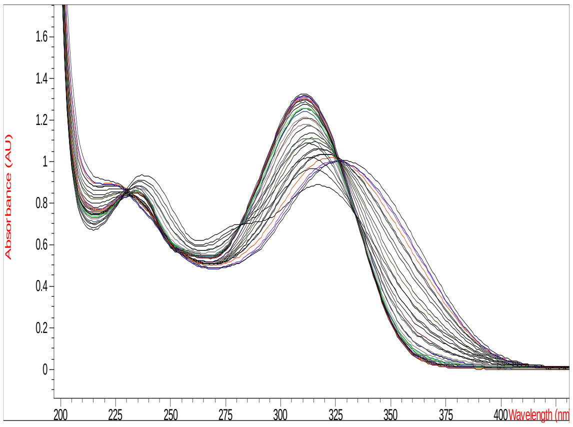

Figure S7: b. Plots of absorbance for 4 wavelength values (λ/nm) as function of pH

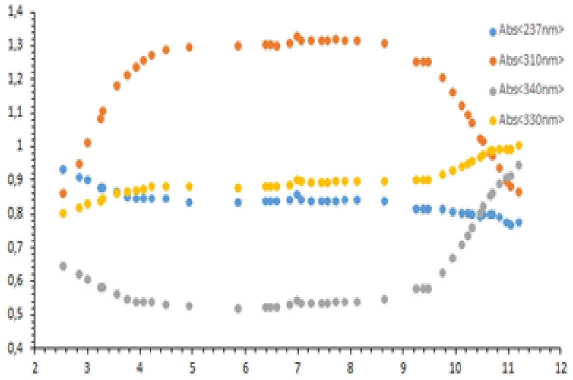

Figure S8: a. UV-*vis* spectra obtained for compound **7** as function of pH variation.

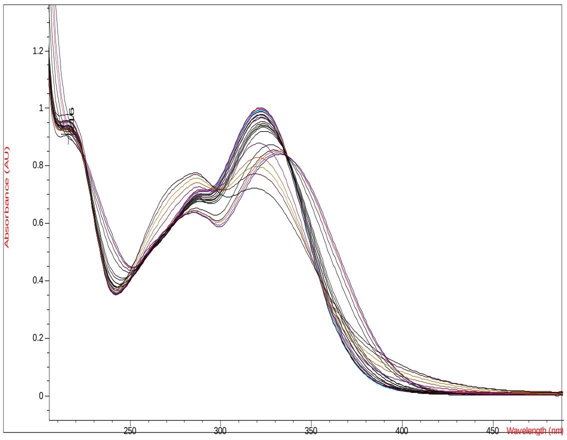

Figure S8: b. Plots of absorbance for 4 wavelength values (λ/nm) as function of pH

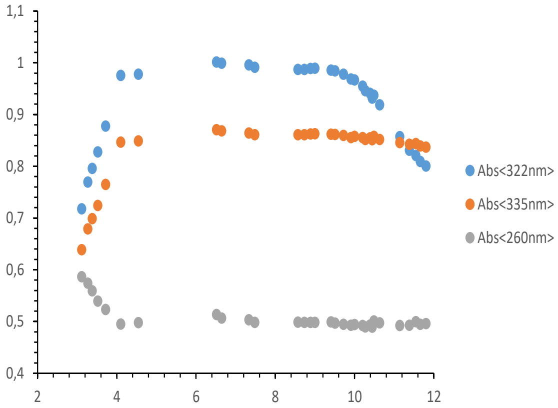

Compound **2**

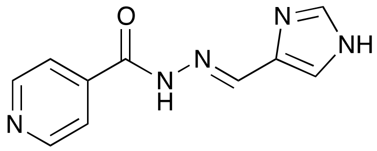

Elemental Composition Report

Single Mass Analysis
Tolerance = 3.0 PPM / DBE: min = -1.5, max = 80.0
Element prediction: Off
Number of isotope peaks used for i-FIT = 3

Monoisotopic Mass, Even Electron Ions
225 formula(e) evaluated with 1 results within limits (up to 20 closest results for each mass)
Elements Used:
C: 0-50 H: 0-100 N: 0-10 O: 0-10
Cone voltage = 30 V XEVO-G2QTOF#YCA210 17-Jan-2017 10:01:44
ISO-1 50 (0.499) AM2 (Ar,14000.0,0.00,0.00); Cm (47:59-31:38x2.000) 1: TOF MS ES+
2.80e+006

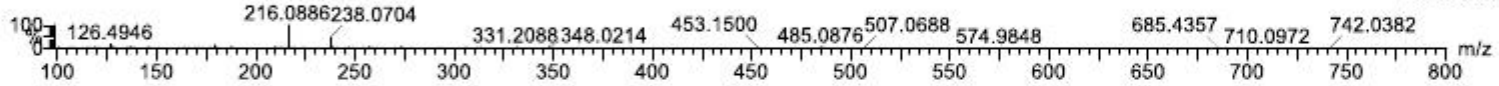

Minimum:				-1.5				
Maximum:		1.0	3.0	80.0				
Mass	Calc. Mass	mDa	PPM	DBE	i-FIT	Norm	Conf(%)	Formula
216.0886	216.0885	0.1	0.5	8.5	691.3	n/a	n/a	C10 H10 N5 O

Cone voltage = 30 V XEVO-G2QTOF#YCA210 17-Jan-2017 10:01:44
ISO-1 50 (0.499) AM2 (Ar,14000.0,0.00,0.00); Cm (47:59-31:38x2.000) 1: TOF MS ES+
2.80e6

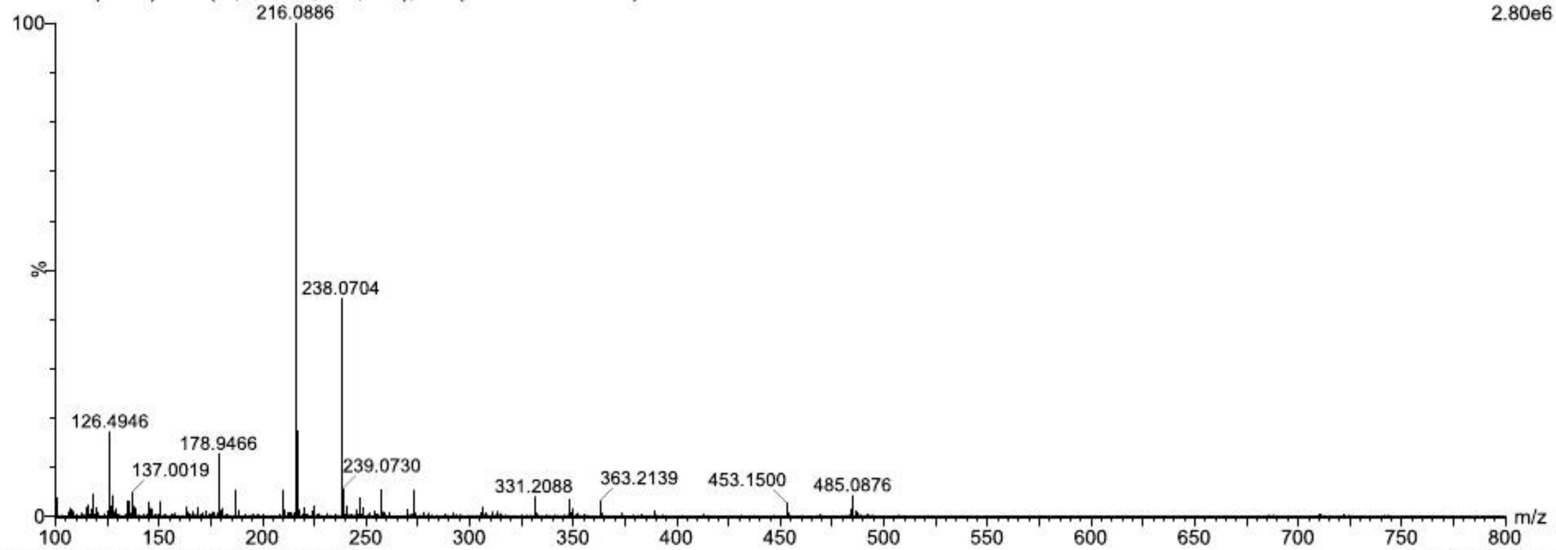

ISO-1 (0.033) Is (1.00,1.00) C10H9N5O 1: TOF MS ES+
8.79e12

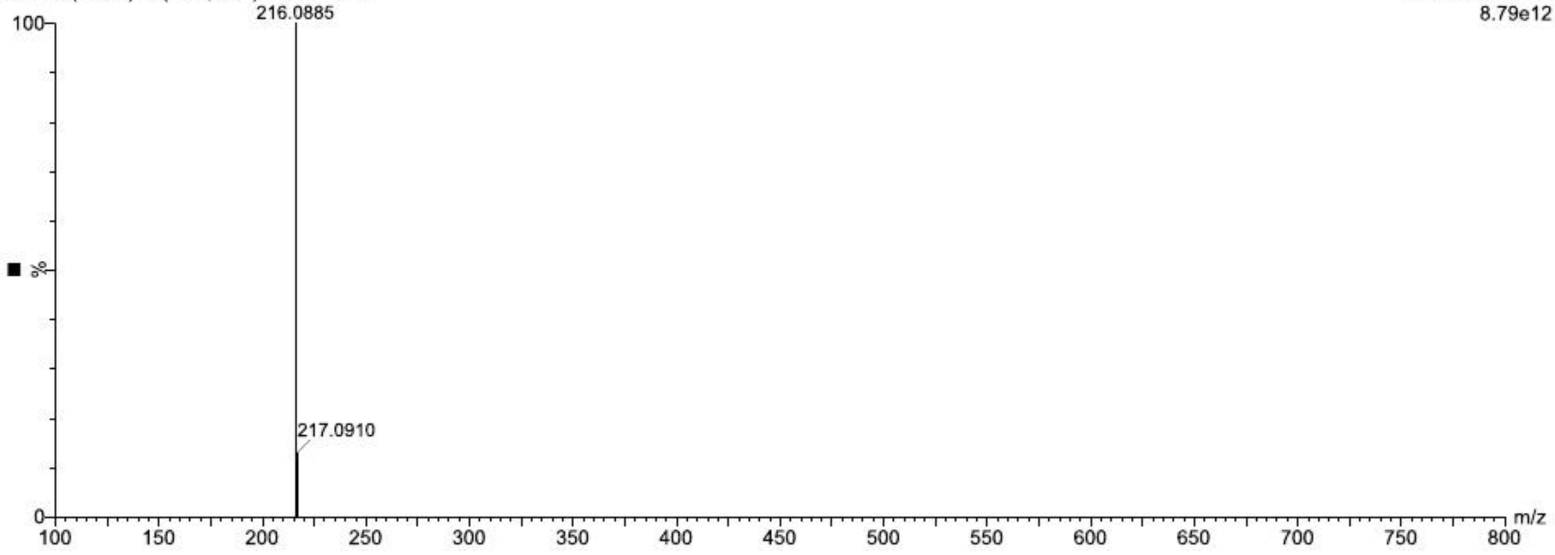

Elemental Composition Report

Compound **3**

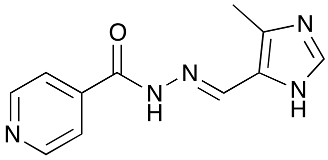

Single Mass Analysis

Tolerance = 3.0 PPM / DBE: min = -1.5, max = 50.0
Element prediction: Off
Number of isotope peaks used for i-FIT = 3

Monoisotopic Mass, Even Electron Ions
321 formula(e) evaluated with 1 results within limits (up to 50 best isotopic matches for each mass)
Elements Used:
C: 0-50 H: 0-100 N: 0-10 O: 0-10

30
ISO 2C 75 (0.493) AM2 (Ar,14000.0,0.00,0.00); Cm (66:75-34:46x2.000) — 1: TOF MS ES+ 1.66e+006

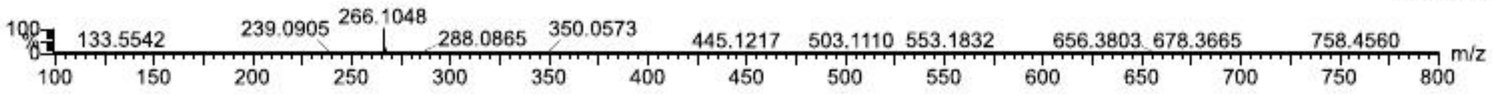

Minimum:				-1.5				
Maximum:		1.0	3.0	50.0				
Mass	Calc. Mass	mDa	PPM	DBE	i-FIT	Norm	Conf(%)	Formula
266.1048	266.1042	0.6	2.3	11.5	431.7	n/a	n/a	C14 H12 N5 O

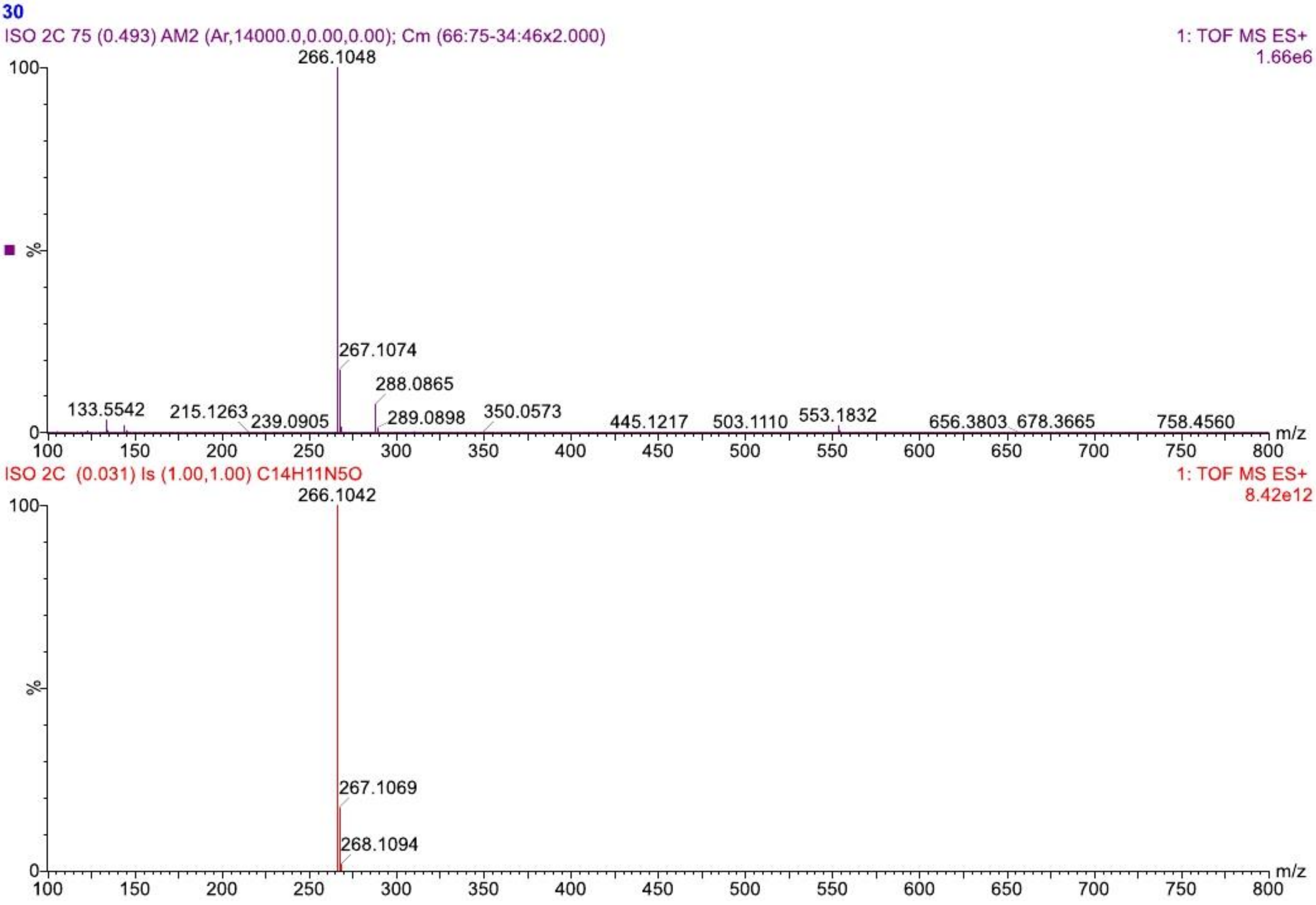

Elemental Composition Report

Compound **4**

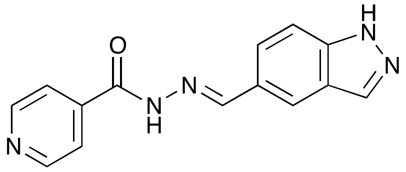

Single Mass Analysis

Tolerance = 3.0 PPM / DBE: min = -1.5, max = 50.0
Element prediction: Off
Number of isotope peaks used for i-FIT = 3

Monoisotopic Mass, Even Electron Ions
321 formula(e) evaluated with 1 results within limits (up to 50 best isotopic matches for each mass)
Elements Used:
C: 0-50 H: 0-100 N: 0-10 O: 0-10

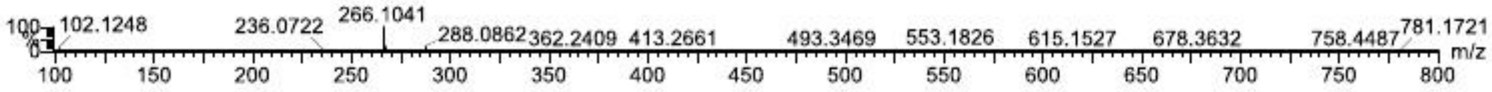

Minimum:				-1.5				
Maximum:		1.0	3.0	50.0				
Mass	Calc. Mass	mDa	PPM	DBE	i-FIT	Norm	Conf(%)	Formula
266.1041	266.1042	-0.1	-0.4	11.5	586.7	n/a	n/a	C14 H12 N5 O

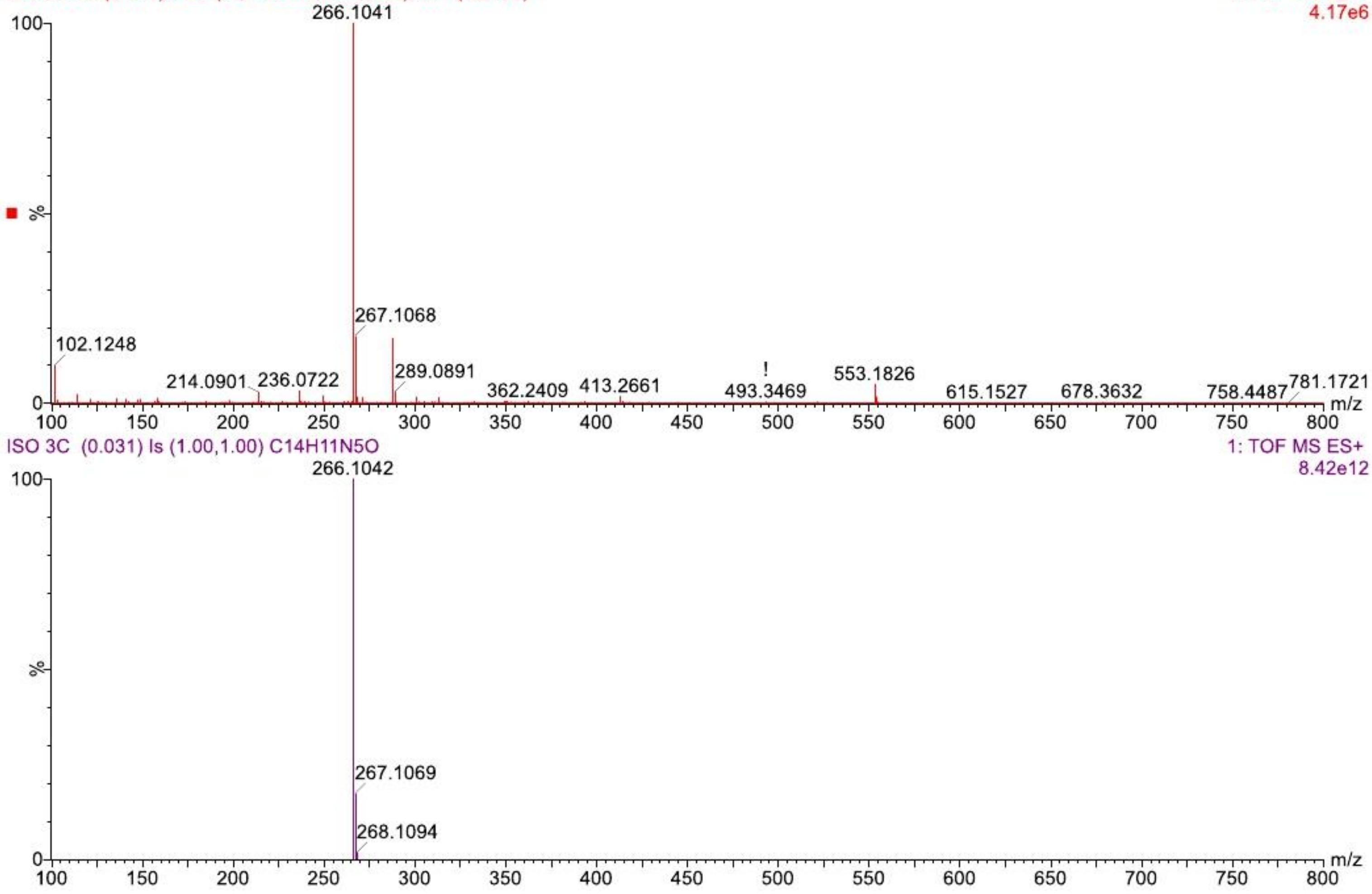

Elemental Composition Report

Compound **5**

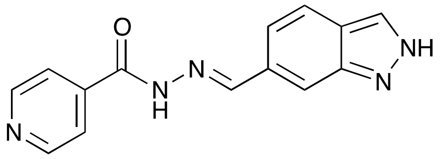

Single Mass Analysis

Tolerance = 3.0 PPM / DBE: min = -1.5, max = 50.0
Element prediction: Off
Number of isotope peaks used for i-FIT = 3

Monoisotopic Mass, Even Electron Ions
321 formula(e) evaluated with 1 results within limits (up to 50 best isotopic matches for each mass)
Elements Used:
C: 0-50 H: 0-100 N: 0-10 O: 0-10
30
ISO 4C 81 (0.526) AM2 (Ar,14000.0,0.00,0.00); Cm (74:89)

1: TOF MS ES+
1.88e+007

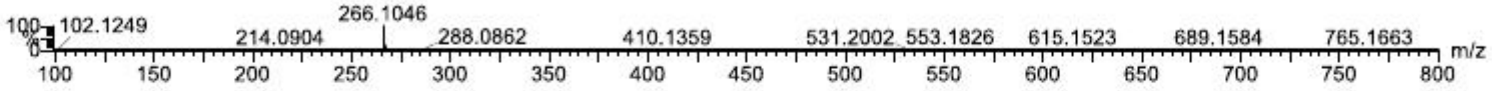

Minimum:				-1.5				
Maximum:		1.0	3.0	50.0				
Mass	Calc. Mass	mDa	PPM	DBE	i-FIT	Norm	Conf(%)	Formula
266.1046	266.1042	0.4	1.5	11.5	721.2	n/a	n/a	C14 H12 N5 O

30
ISO 4C 81 (0.526) AM2 (Ar,14000.0,0.00,0.00); Cm (74:89)

1: TOF MS ES+
1.88e7

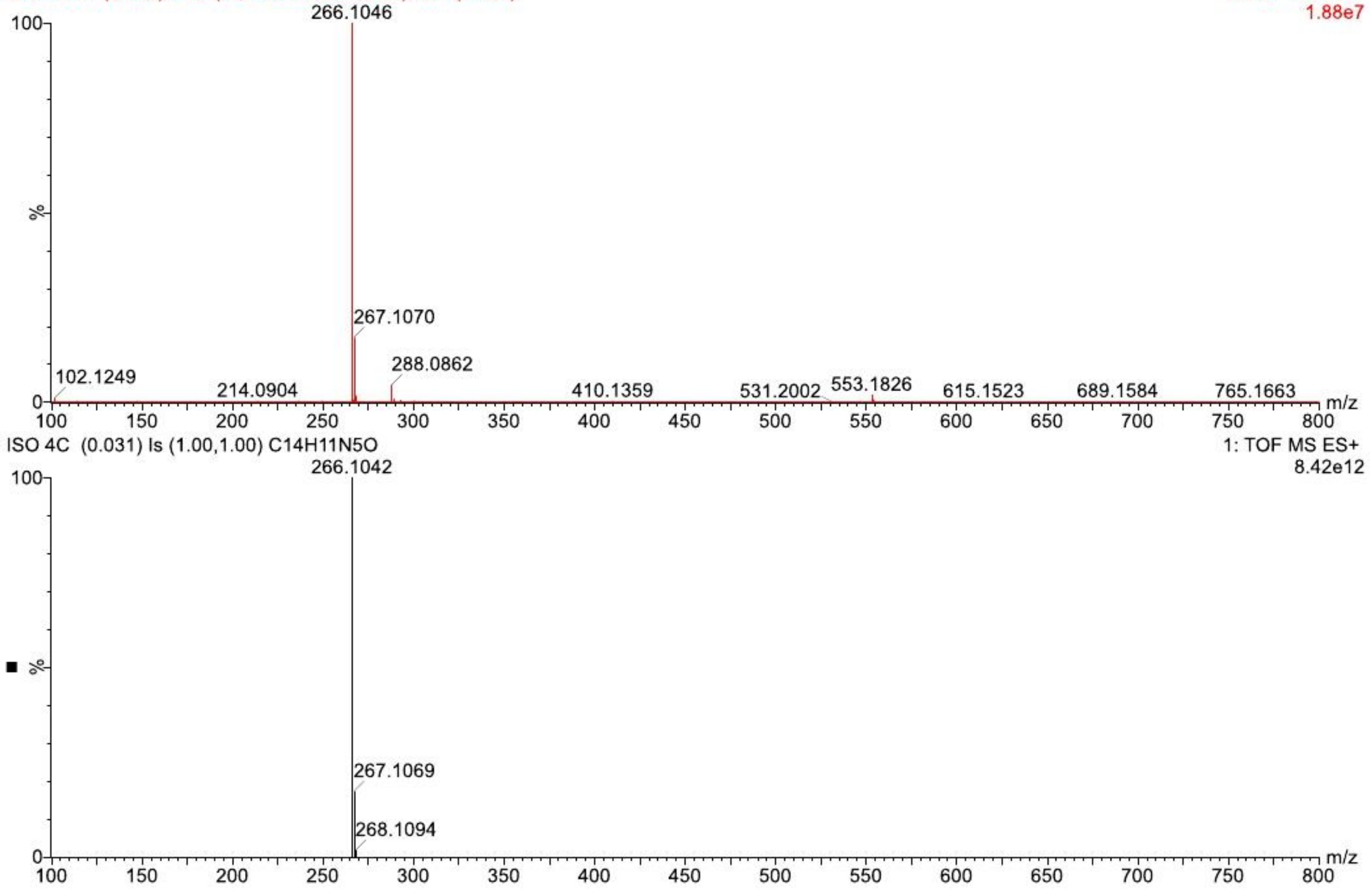

Elemental Composition Report

Compound **6**

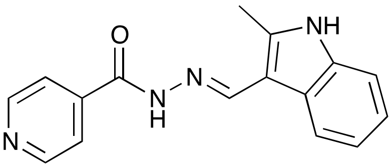

Single Mass Analysis

Tolerance = 3.0 PPM / DBE: min = -1.5, max = 50.0
Element prediction: Off
Number of isotope peaks used for i-FIT = 3

Monoisotopic Mass, Even Electron Ions
352 formula(e) evaluated with 1 results within limits (up to 50 best isotopic matches for each mass)
Elements Used:
C: 0-50 H: 0-100 N: 0-10 O: 0-10

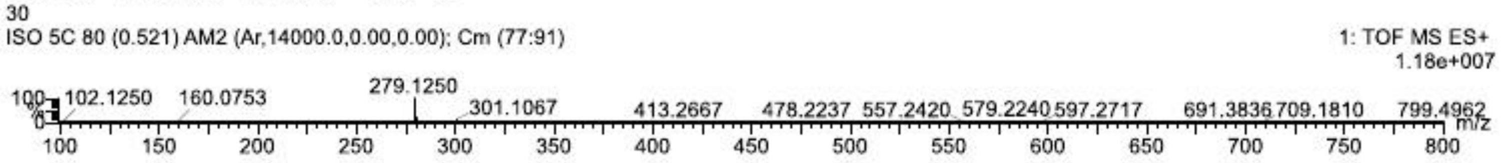

Minimum:				-1.5				
Maximum:		1.0	3.0	50.0				
Mass	Calc. Mass	mDa	PPM	DBE	i-FIT	Norm	Conf(%)	Formula
279.1250	279.1246	0.4	1.4	11.5	701.7	n/a	n/a	C16 H15 N4 O

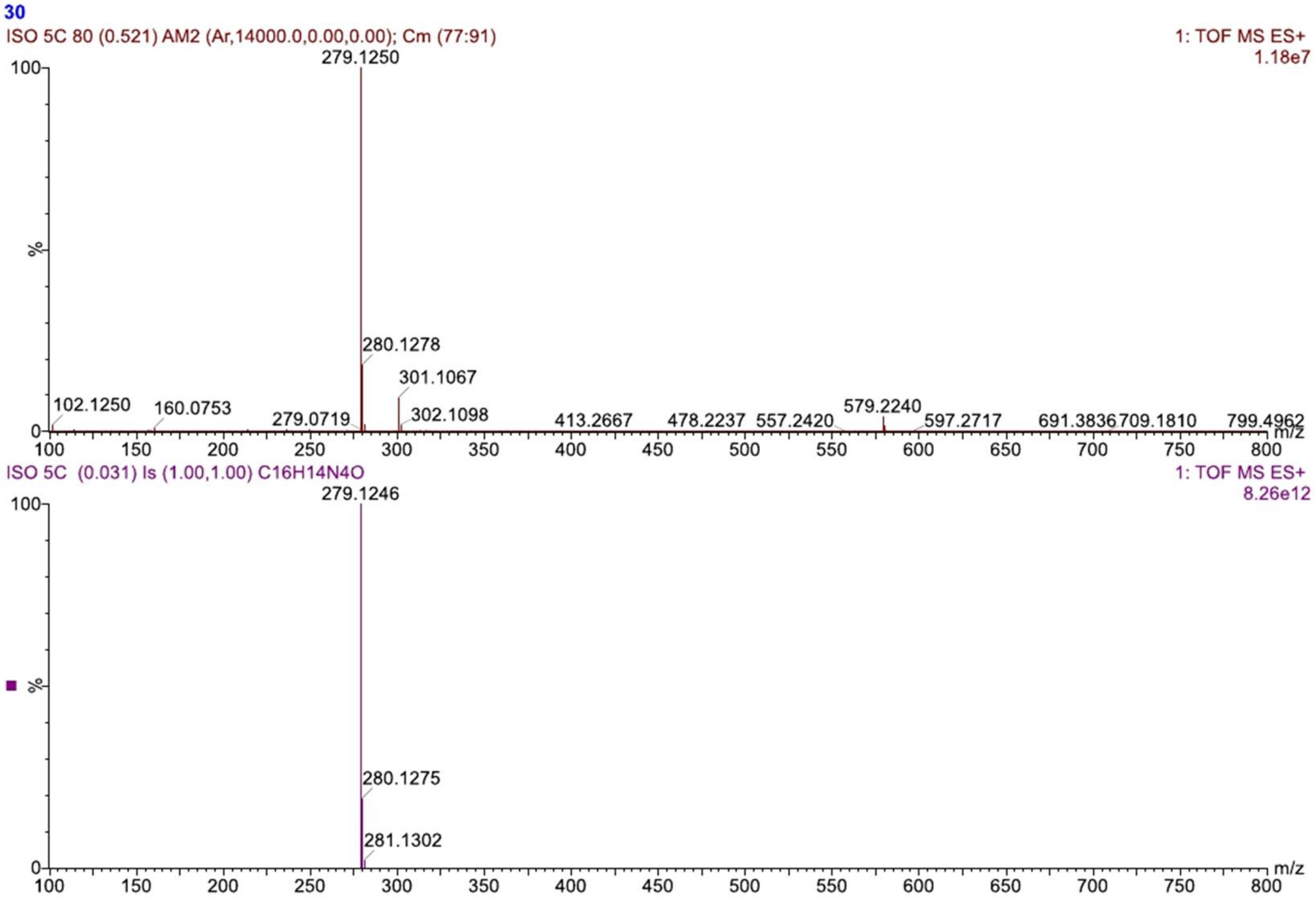

Compound **7**

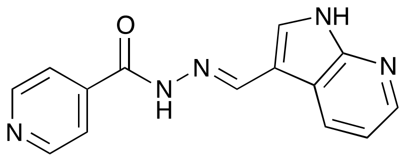

Single Mass Analysis

Tolerance = 3.0 PPM / DBE: min = -1.5, max = 50.0
Element prediction: Off
Number of isotope peaks used for i-FIT = 3

Monoisotopic Mass, Even Electron Ions
248 formula(e) evaluated with 1 results within limits (up to 50 best isotopic matches for each mass)
Elements Used:
C: 0-50 H: 0-100 N: 0-10 O: 0-10

30
ISO 6C 77 (0.504) AM2 (Ar,14000.0,0.00,0.00); Cm (71:90-9:51x2.000) 1: TOF MS ES+ 1.34e+006

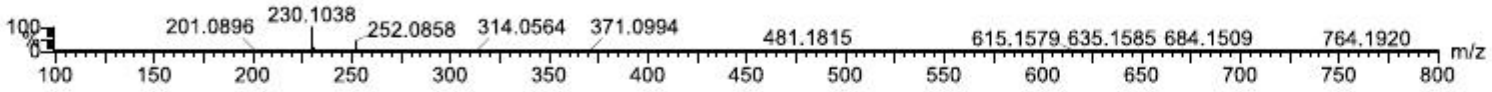

Minimum:				-1.5				
Maximum:		1.0	3.0	50.0				
Mass	Calc. Mass	mDa	PPM	DBE	i-FIT	Norm	Conf(%)	Formula
230.1038	230.1042	-0.4	-1.7	8.5	370.9	n/a	n/a	C11 H12 N5 O

30
ISO 6C 77 (0.504) AM2 (Ar,14000.0,0.00,0.00); Cm (71:90-9:51x2.000) 1: TOF MS ES+ 1.34e6

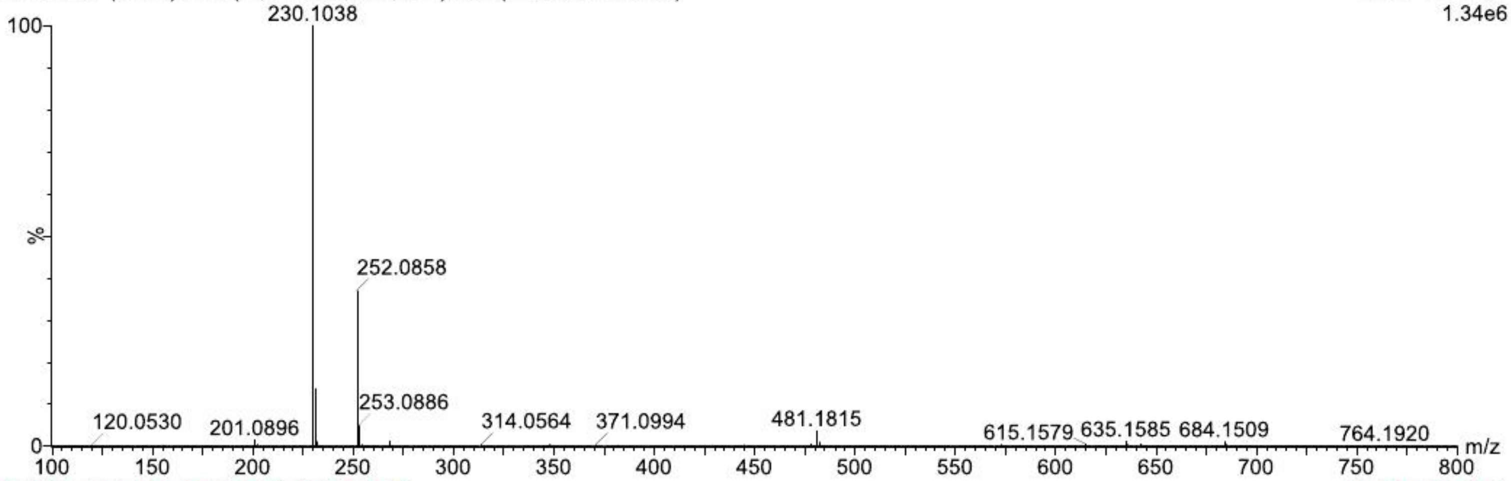

ISO 6C (0.031) Is (1.00,1.00) C11H11N5O 1: TOF MS ES+ 8.69e12

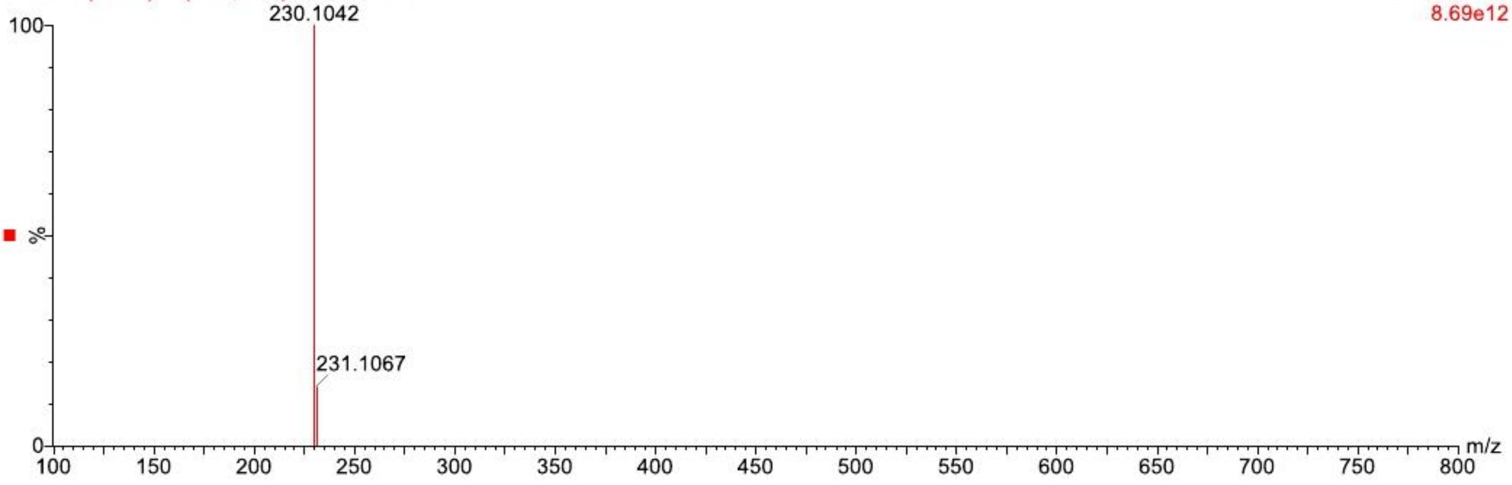

Elemental Composition Report

Compound **8**

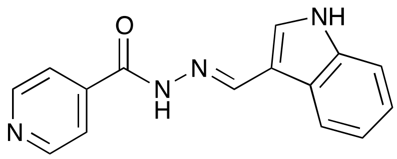

Single Mass Analysis

Tolerance = 3.0 PPM / DBE: min = -1.5, max = 50.0
Element prediction: Off
Number of isotope peaks used for i-FIT = 3

Monoisotopic Mass, Even Electron Ions
327 formula(e) evaluated with 1 results within limits (up to 50 best isotopic matches for each mass)
Elements Used:
C: 0-50 H: 0-100 N: 0-10 O: 0-10

30
ISO 7C 80 (0.521) AM2 (Ar,14000.0,0.00,0.00); Cm (71:96) — 1: TOF MS ES+ 1.71e+007

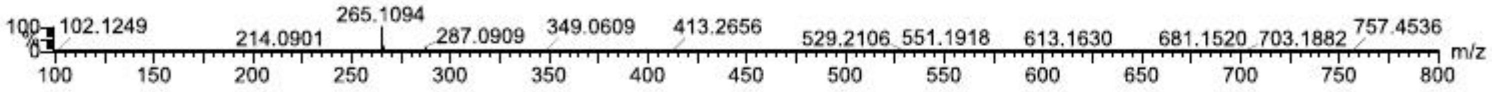

Minimum:				-1.5				
Maximum:		1.0	3.0	50.0				
Mass	Calc. Mass	mDa	PPM	DBE	i-FIT	Norm	Conf(%)	Formula
265.1094	265.1089	0.5	1.9	11.5	703.6	n/a	n/a	C15 H13 N4 O

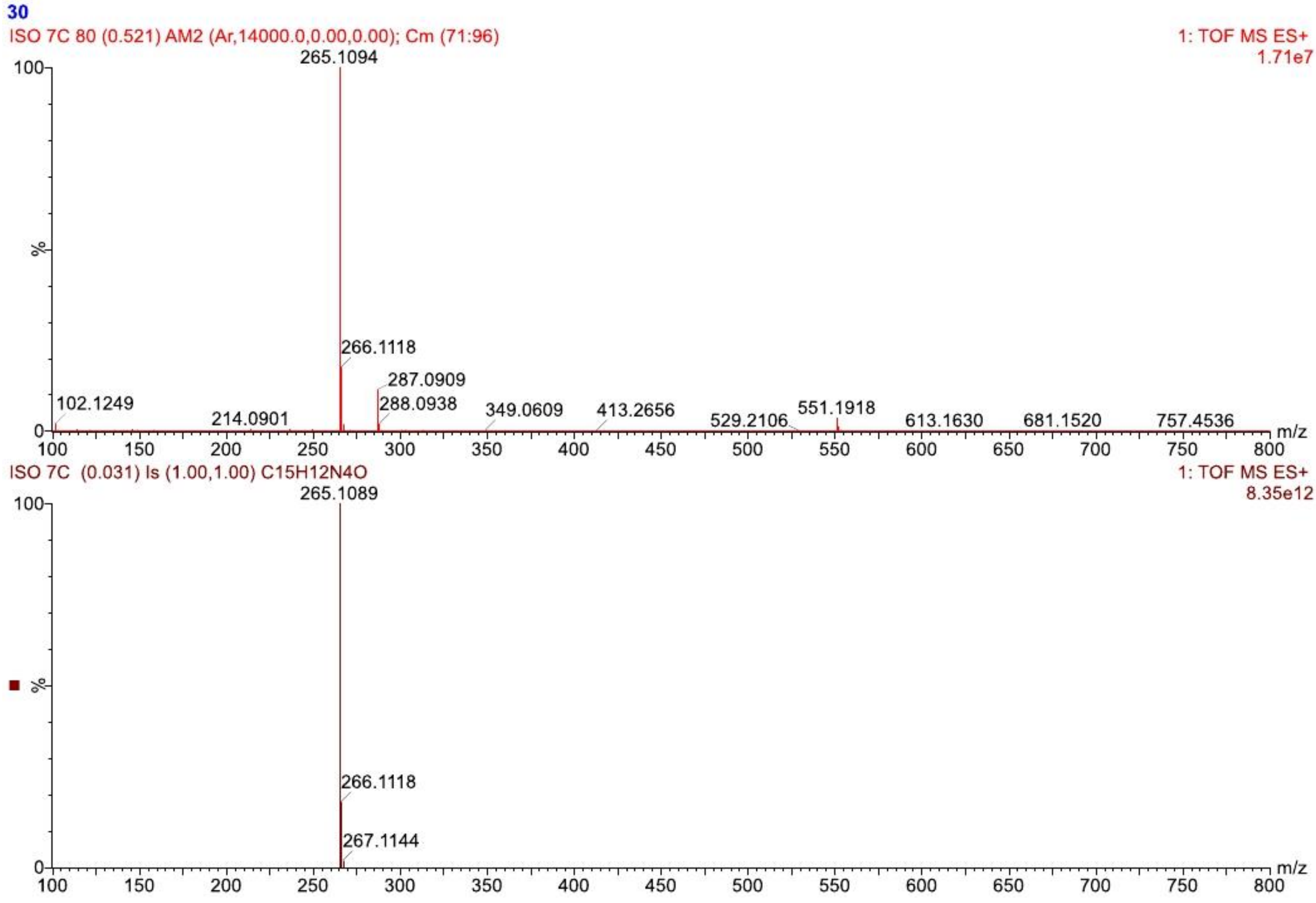

Elemental Composition Report

Compound **9**

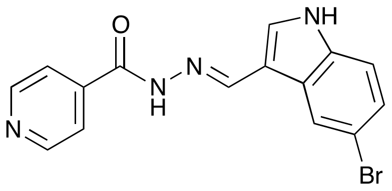

Single Mass Analysis

Tolerance = 3.0 PPM / DBE: min = -1.5, max = 50.0
Element prediction: Off
Number of isotope peaks used for i-FIT = 3

Monoisotopic Mass, Even Electron Ions
759 formula(e) evaluated with 3 results within limits (up to 50 best isotopic matches for each mass)
Elements Used:
C: 0-50 H: 0-100 N: 0-10 O: 0-10 Br: 0-1

30
ISO 8C 78 (0.510) AM2 (Ar,14000.0,0.00,0.00); Cm (74:96-4:55x2.000) 1: TOF MS ES+ 1.90e+005

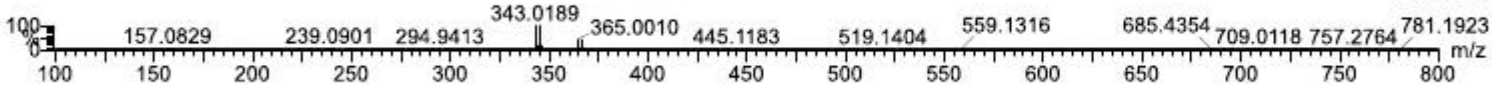

Minimum:				-1.5				
Maximum:		1.0	3.0	50.0				
Mass	Calc. Mass	mDa	PPM	DBE	i-FIT	Norm	Conf(%)	Formula
343.0189	343.0181	0.8	2.3	6.5	185.7	0.075	92.78	C14 H16 O5 Br
	343.0194	-0.5	-1.5	11.5	188.3	2.628	7.22	C15 H12 N4 O Br
	343.0184	0.5	1.5	26.5	212.0	26.401	0.00	C27 H3 O

30
ISO 8C 78 (0.510) AM2 (Ar,14000.0,0.00,0.00); Cm (74:96-4:55x2.000) 1: TOF MS ES+ 1.90e5

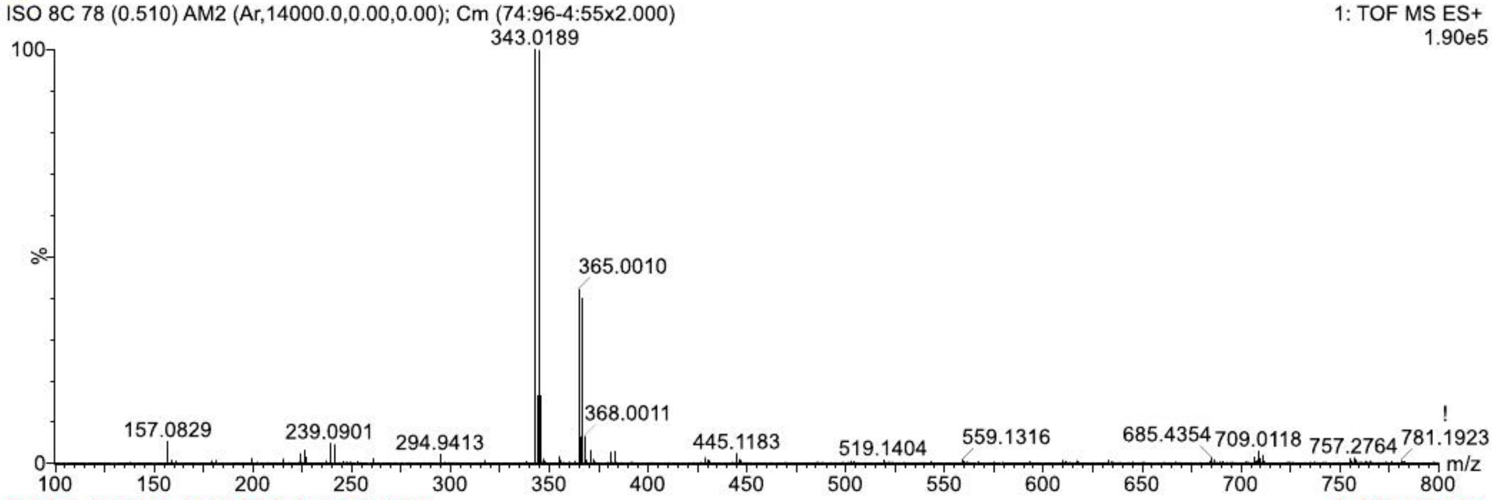

ISO 8C (0.031) Is (1.00,1.00) C15H11N4OBr 1: TOF MS ES+ 4.24e12

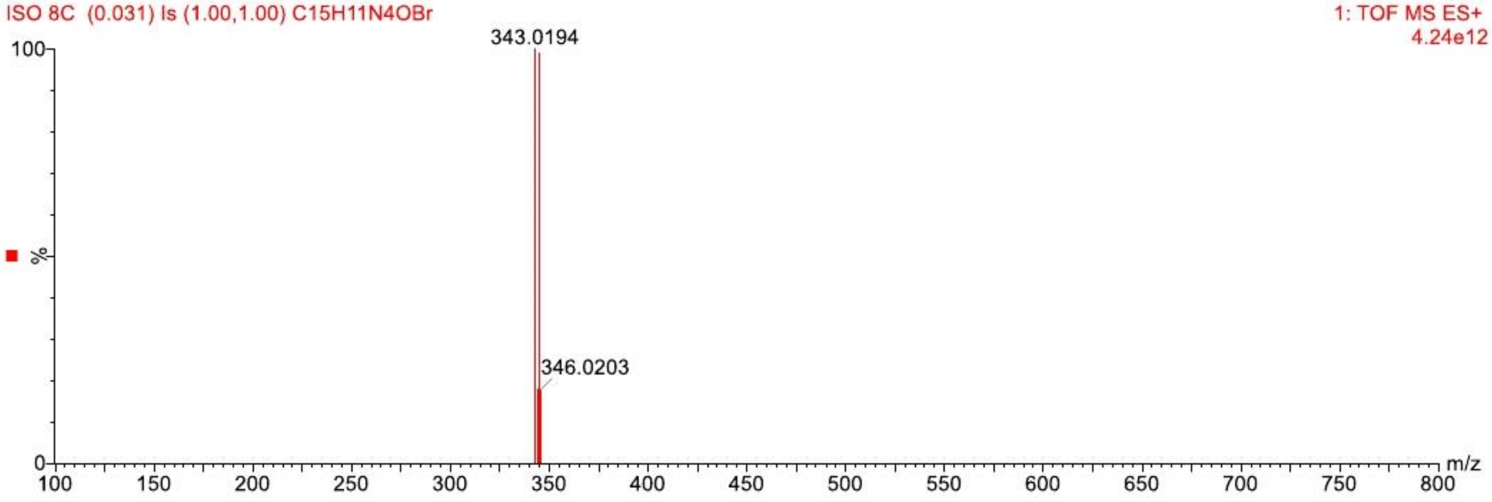

Elemental Composition Report

Compound **10**

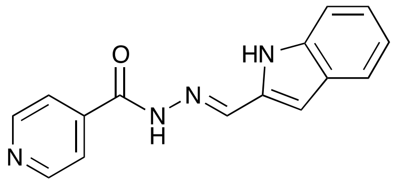

Single Mass Analysis

Tolerance = 3.0 PPM / DBE: min = -1.5, max = 50.0
Element prediction: Off
Number of isotope peaks used for i-FIT = 3

Monoisotopic Mass, Even Electron Ions
327 formula(e) evaluated with 1 results within limits (up to 50 best isotopic matches for each mass)
Elements Used:
C: 0-50 H: 0-100 N: 0-10 O: 0-10

30
ISO 9C 79 (0.515) AM2 (Ar,14000.0,0.00,0.00); Cm (71:94)

1: TOF MS ES+
6.52e+006

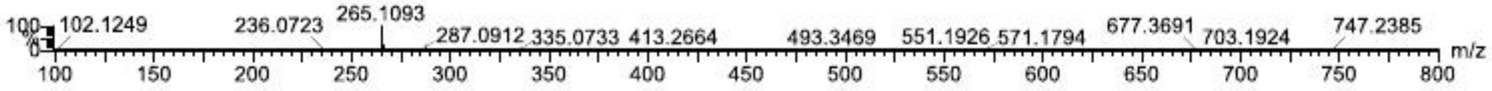

Minimum:				-1.5				
Maximum:		1.0	3.0	50.0				
Mass	Calc. Mass	mDa	PPM	DBE	i-FIT	Norm	Conf(%)	Formula
265.1093	265.1089	0.4	1.5	11.5	621.3	n/a	n/a	C15 H13 N4 O

30
ISO 9C 79 (0.515) AM2 (Ar,14000.0,0.00,0.00); Cm (71:94)

1: TOF MS ES+
6.52e6

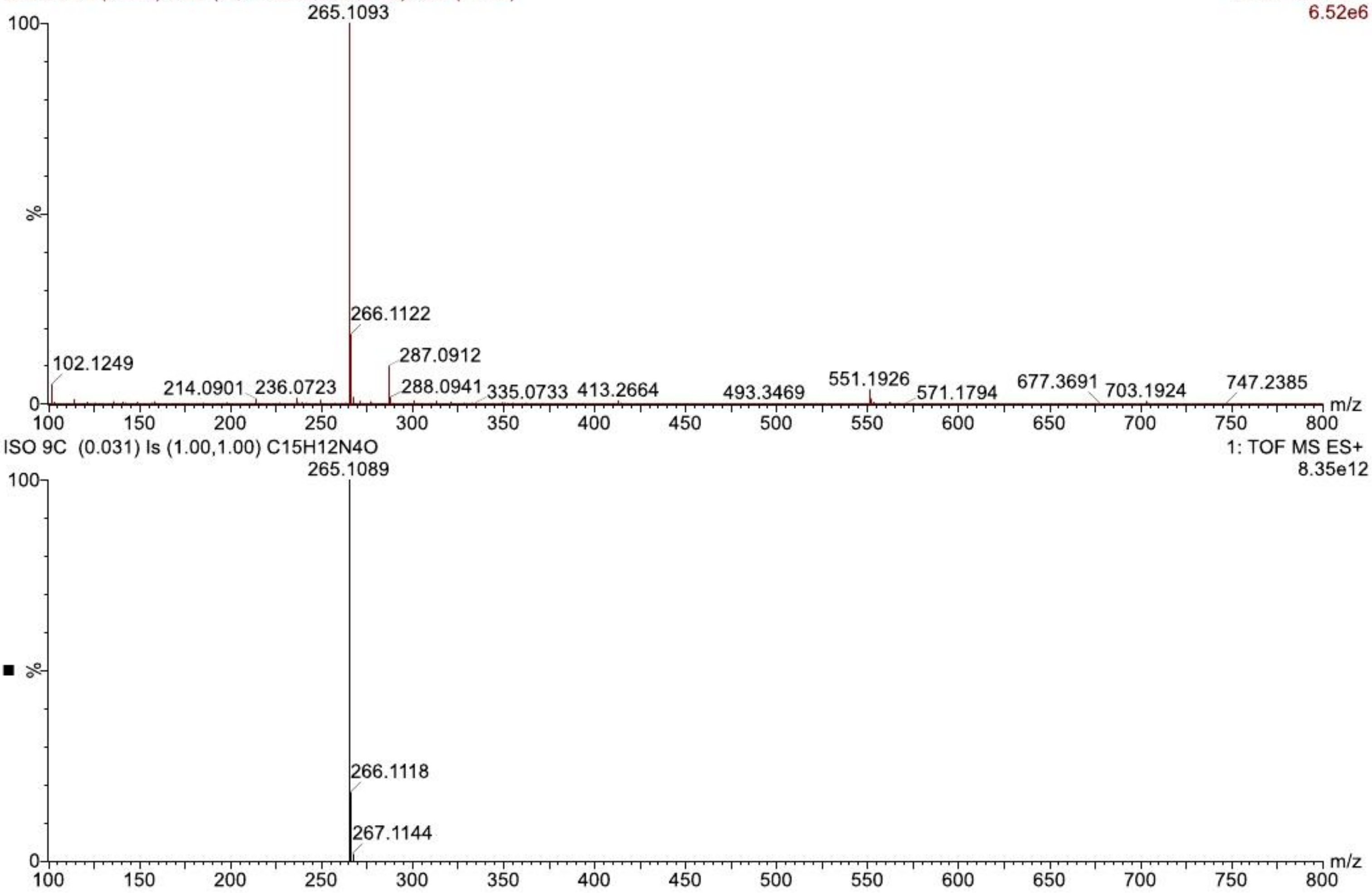

Elemental Composition Report

Compound **11**

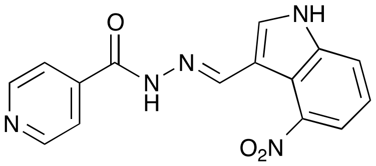

Single Mass Analysis

Tolerance = 3.0 PPM / DBE: min = -1.5, max = 50.0
Element prediction: Off
Number of isotope peaks used for i-FIT = 3

Monoisotopic Mass, Even Electron Ions
393 formula(e) evaluated with 1 results within limits (up to 50 best isotopic matches for each mass)
Elements Used:
C: 0-50 H: 0-100 N: 0-10 O: 0-10

30
ISO 10C 79 (0.515) AM2 (Ar,14000.0,0.00,0.00); Cm (74:92-31:51x2.000) 1: TOF MS ES+ 1.23e+006

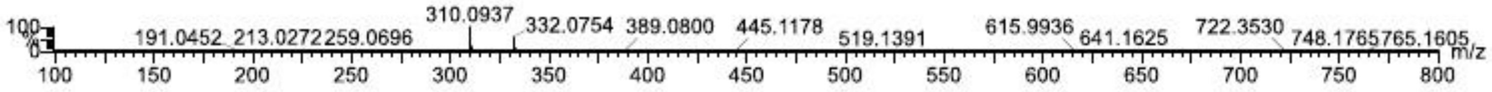

Minimum:				-1.5				
Maximum:		1.0	3.0	50.0				
Mass	Calc. Mass	mDa	PPM	DBE	i-FIT	Norm	Conf(%)	Formula
310.0937	310.0940	-0.3	-1.0	12.5	367.8	n/a	n/a	C15 H12 N5 O3

30
ISO 10C 79 (0.515) AM2 (Ar,14000.0,0.00,0.00); Cm (74:92-31:51x2.000) 1: TOF MS ES+ 1.23e6

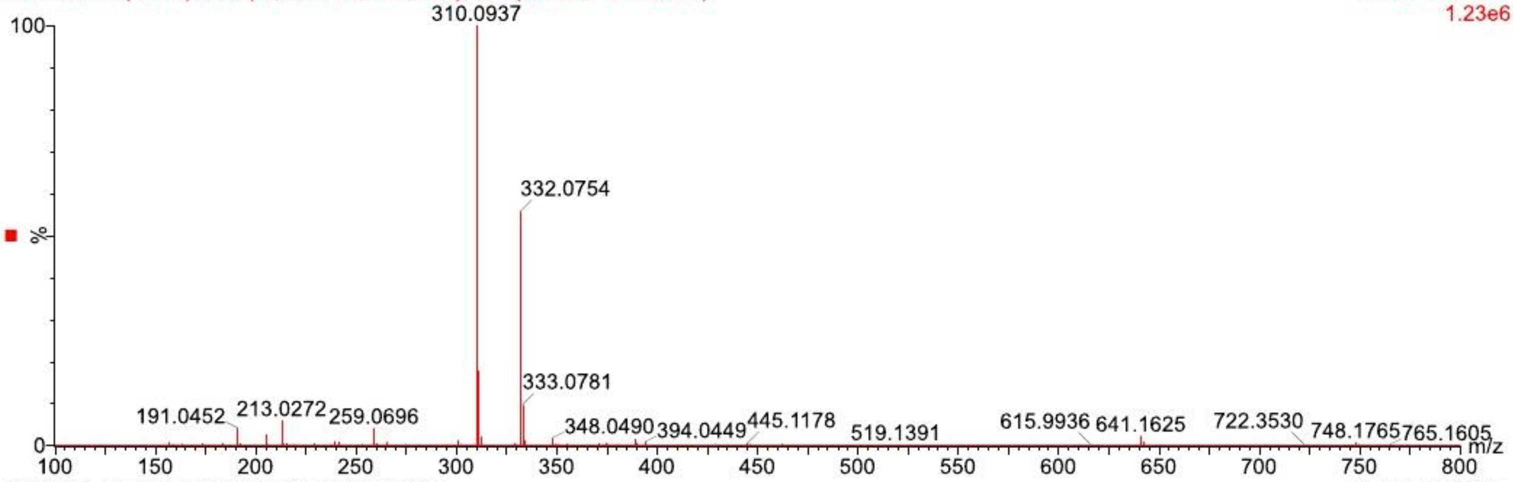

ISO 10C (0.031) Is (1.00,1.00) C15H11N5O3 1: TOF MS ES+ 8.28e12

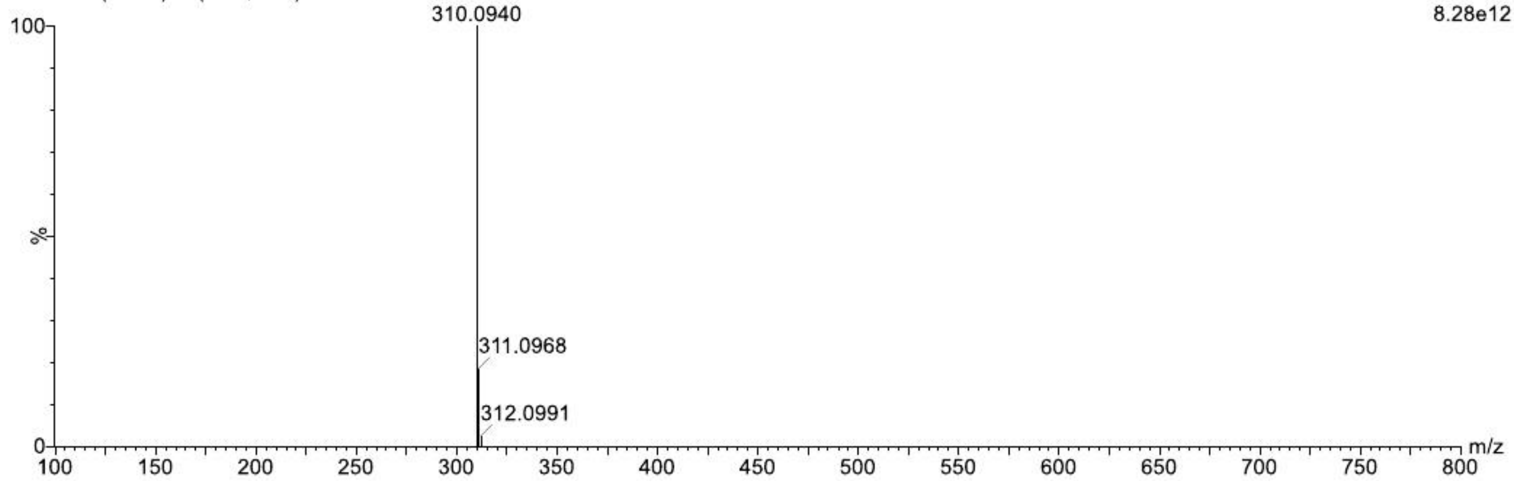

^{1}H NMR

2

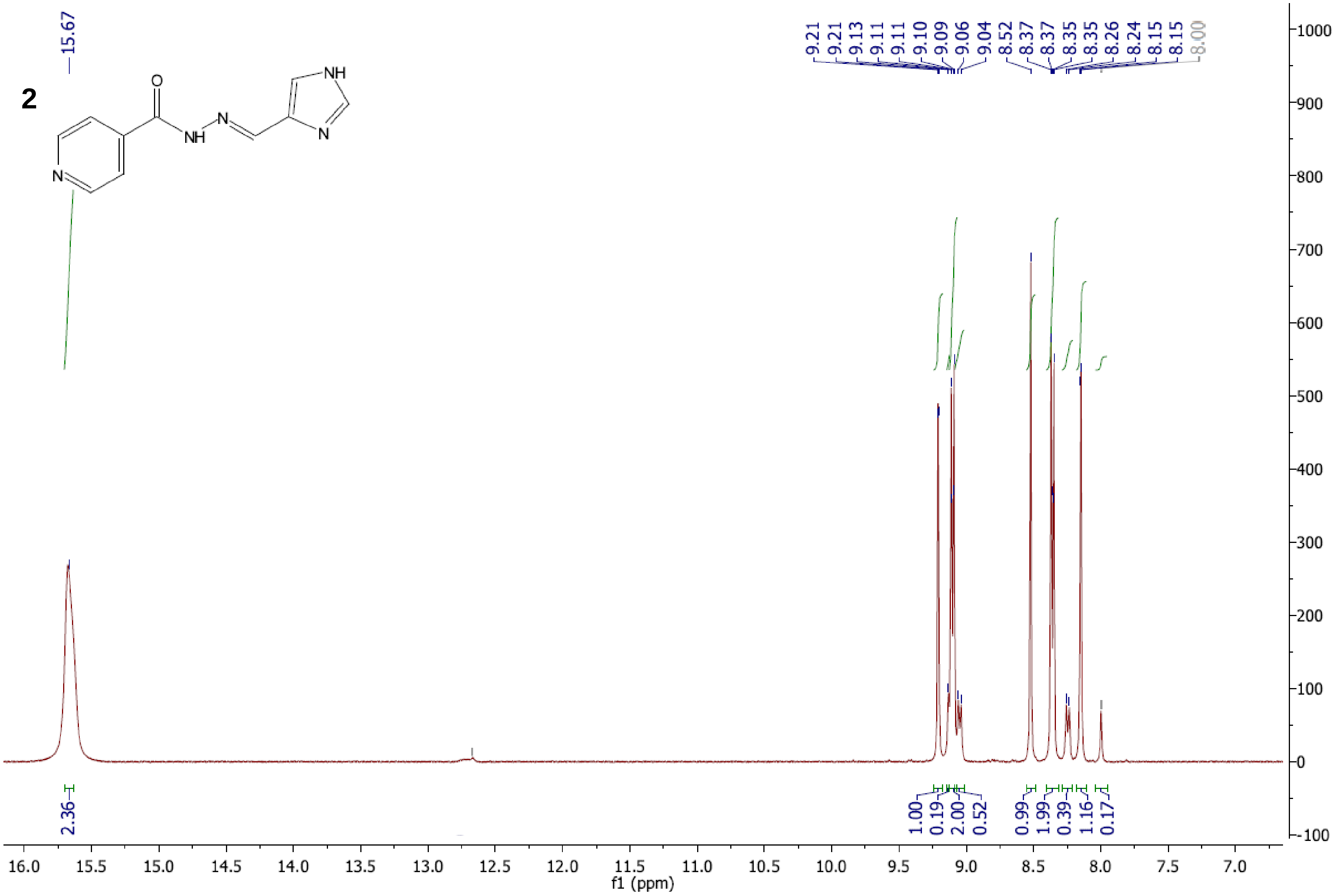
15.67
9.21
9.21
9.13
9.11
9.11
9.10
9.09
9.06
9.04
8.52
8.37
8.37
8.35
8.35
8.26
8.24
8.15
8.15
8.00
2.36
1.00
0.19
2.00
0.52
0.99
1.99
0.39
1.16
0.17
f1 (ppm)

^{13}C NMR

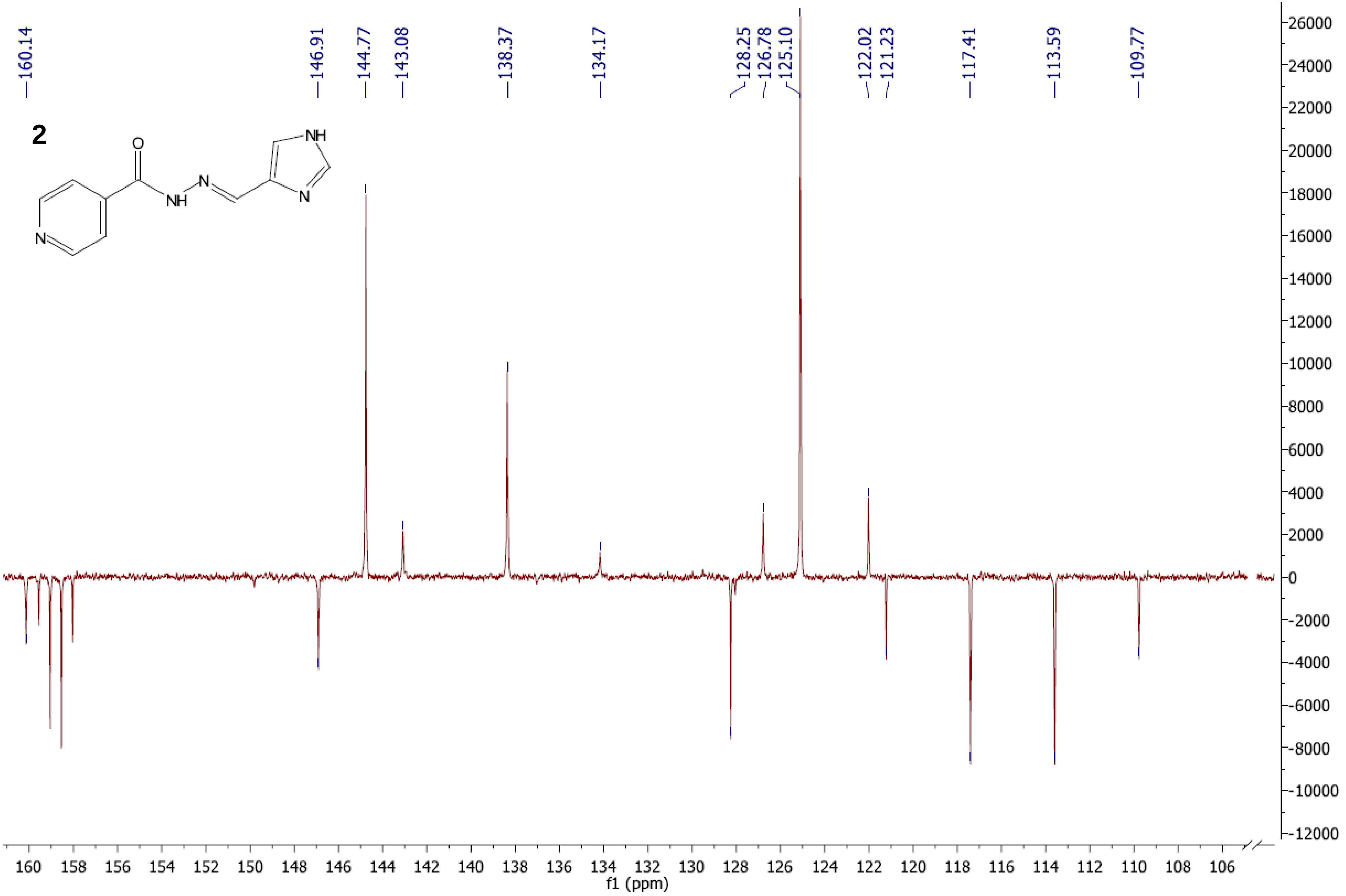

160.14
146.91
144.77
143.08
138.37
134.17
128.25
126.78
125.10
122.02
121.23
117.41
113.59
109.77
2
O
NH
N
NH
N
N
f1 (ppm)

^{1}H NMR

3

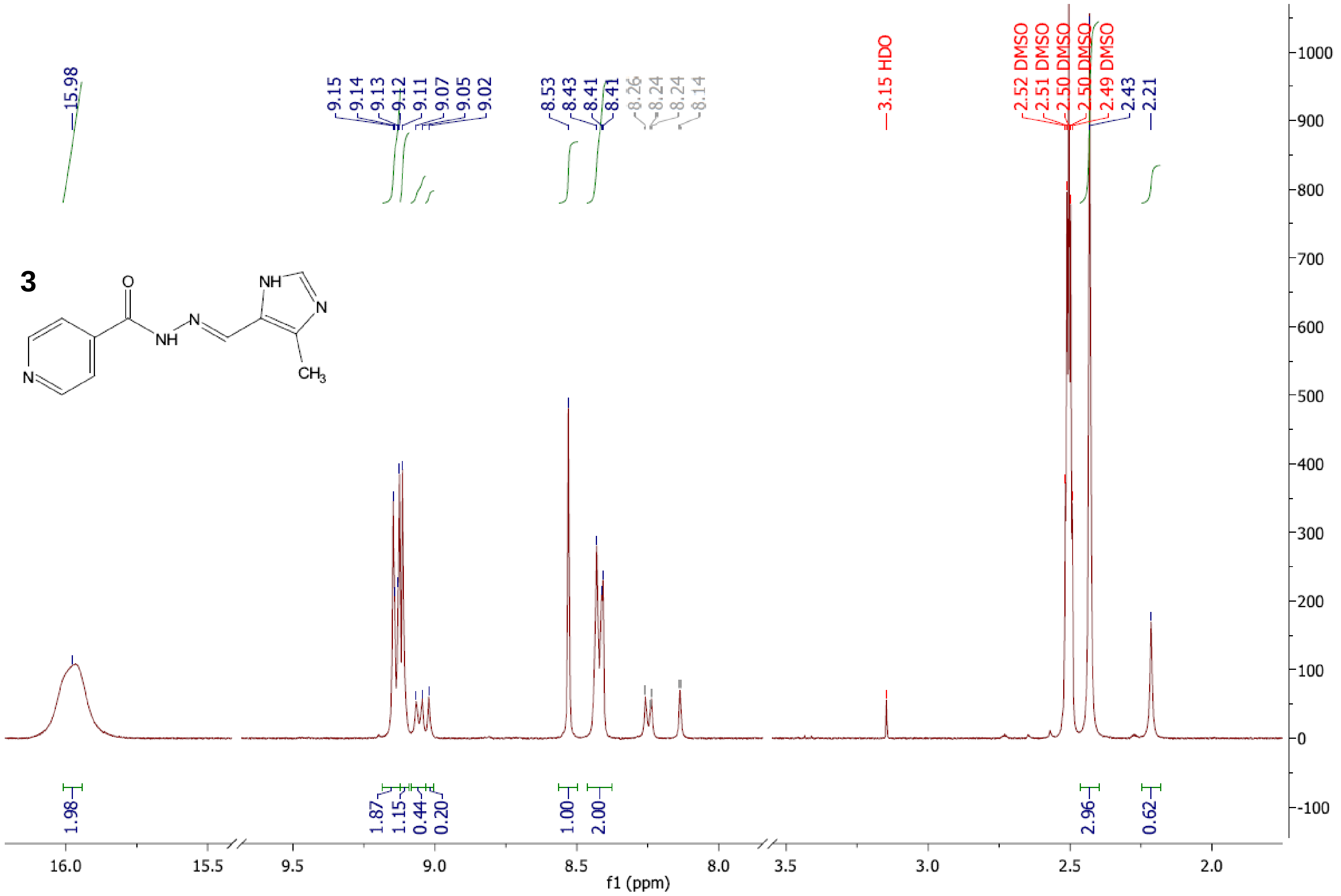
15.98
9.15
9.14
9.13
9.12
9.11
9.07
9.05
9.02
8.53
8.43
8.41
8.41
8.26
8.24
8.24
8.14
3.15 HDO
2.52 DMSO
2.51 DMSO
2.50 DMSO
2.50 DMSO
2.49 DMSO
2.43
2.21
1.98
1.87
1.15
0.44
0.20
1.00
2.00
2.96
0.62
f1 (ppm)
O
NH
N
NH
N
N
CH3

^{13}C NMR

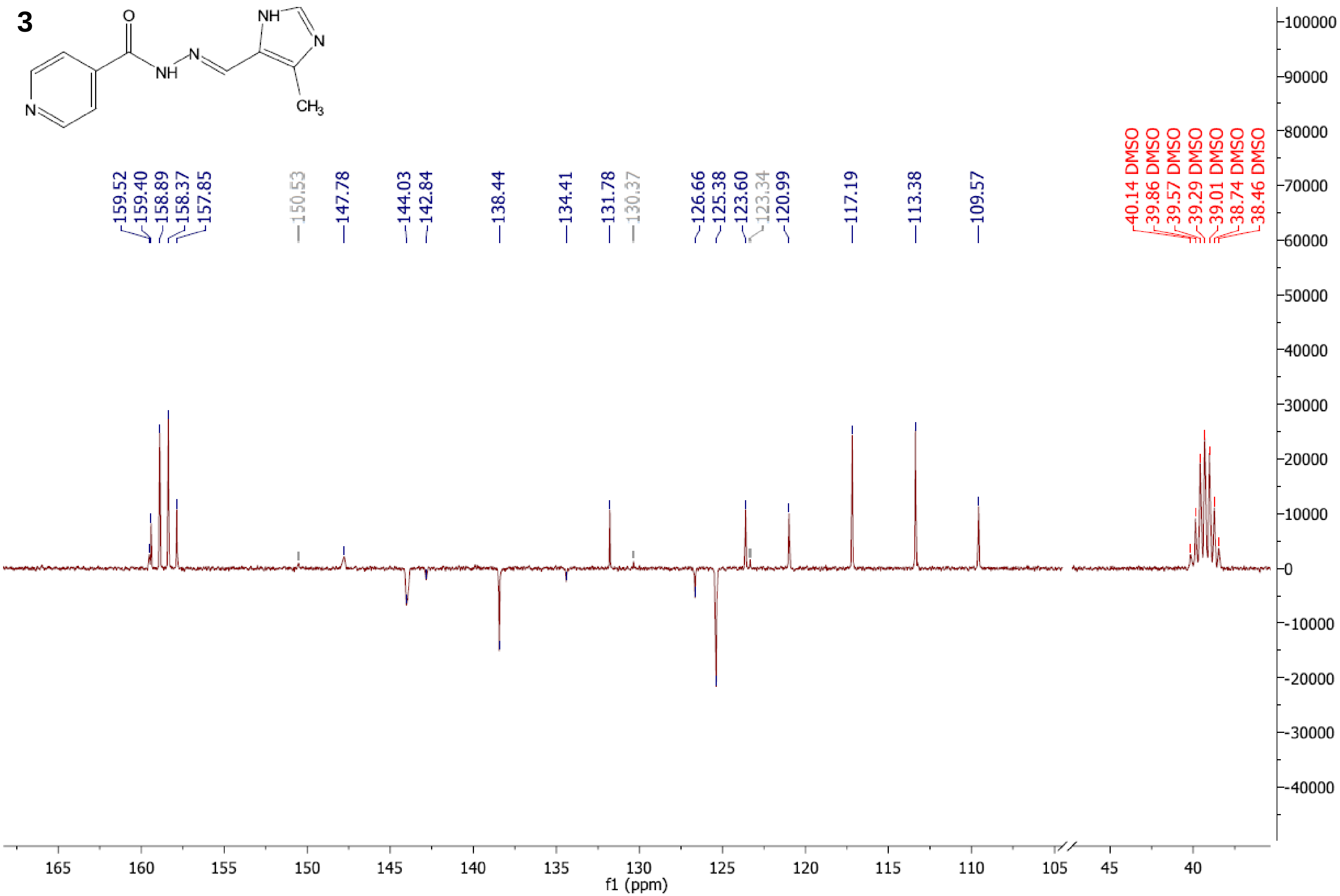
3
O
NH
N
NH
N
N
CH3
159.52
159.40
158.89
158.37
157.85
150.53
147.78
144.03
142.84
138.44
134.41
131.78
130.37
126.66
125.38
123.60
123.34
120.99
117.19
113.38
109.57
40.14 DMSO
39.86 DMSO
39.57 DMSO
39.29 DMSO
39.01 DMSO
38.74 DMSO
38.46 DMSO
f1 (ppm)

^{1}H NMR

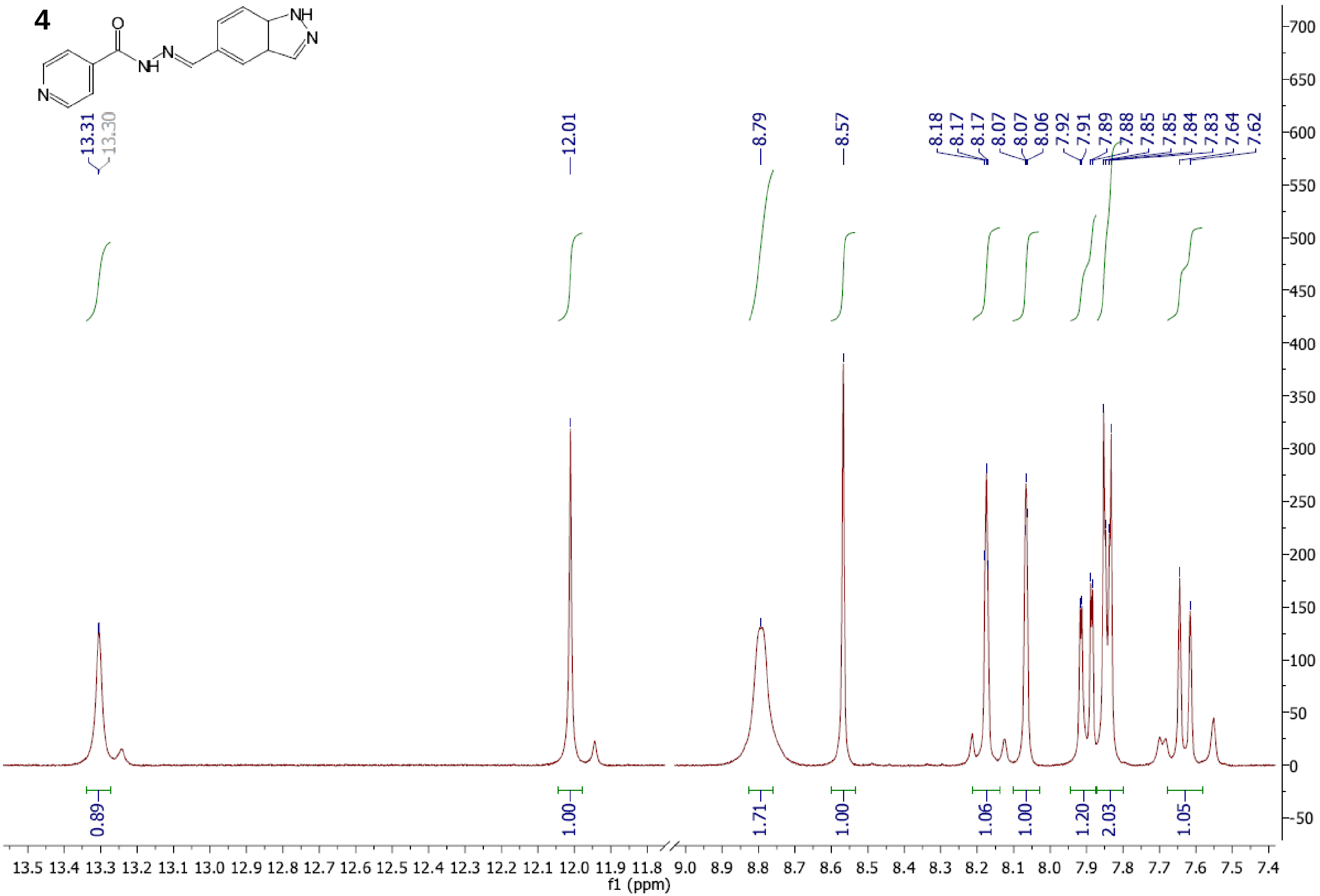

4
O
NH
N
NH
N
N
13.31
13.30
12.01
8.79
8.57
8.18
8.17
8.17
8.07
8.07
8.06
7.92
7.91
7.89
7.88
7.85
7.85
7.84
7.83
7.64
7.62
0.89
1.00
1.71
1.00
1.06
1.00
1.20
2.03
1.05
13.5 13.4 13.3 13.2 13.1 13.0 12.9 12.8 12.7 12.6 12.5 12.4 12.3 12.2 12.1 12.0 11.9 11.8 9.0 8.9 8.8 8.7 8.6 8.5 8.4 8.3 8.2 8.1 8.0 7.9 7.8 7.7 7.6 7.5 7.4
f1 (ppm)
700
650
600
550
500
450
400
350
300
250
200
150
100
50
0
-50

^{13}C NMR

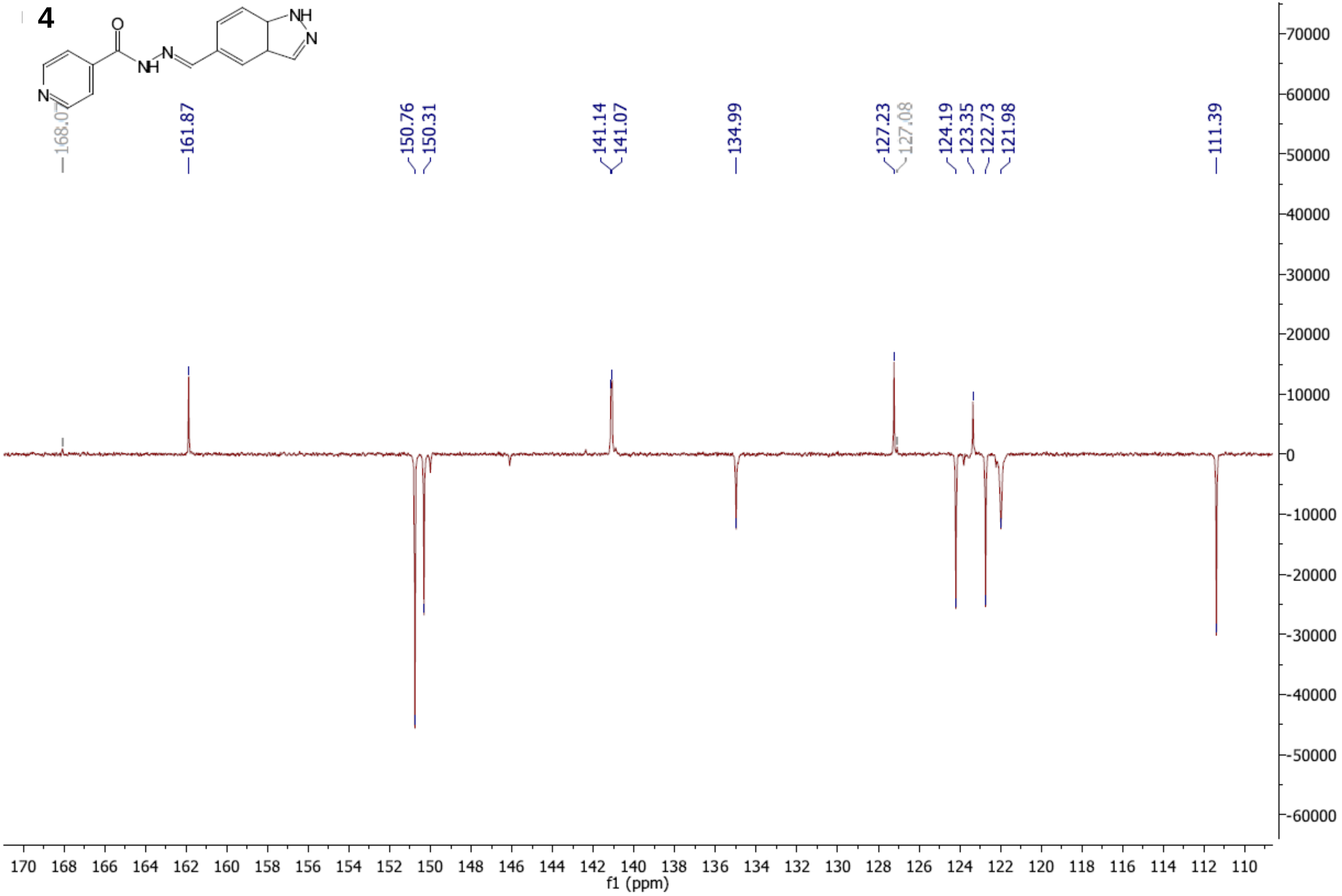
4
O
NH
N
NH
N
168.07
161.87
150.76
150.31
141.14
141.07
134.99
127.23
127.08
124.19
123.35
122.73
121.98
111.39
70000
60000
50000
40000
30000
20000
10000
0
-10000
-20000
-30000
-40000
-50000
-60000
170 168 166 164 162 160 158 156 154 152 150 148 146 144 142 140 138 136 134 132 130 128 126 124 122 120 118 116 114 112 110
f1 (ppm)

^{1}H NMR

5

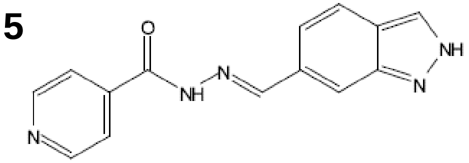

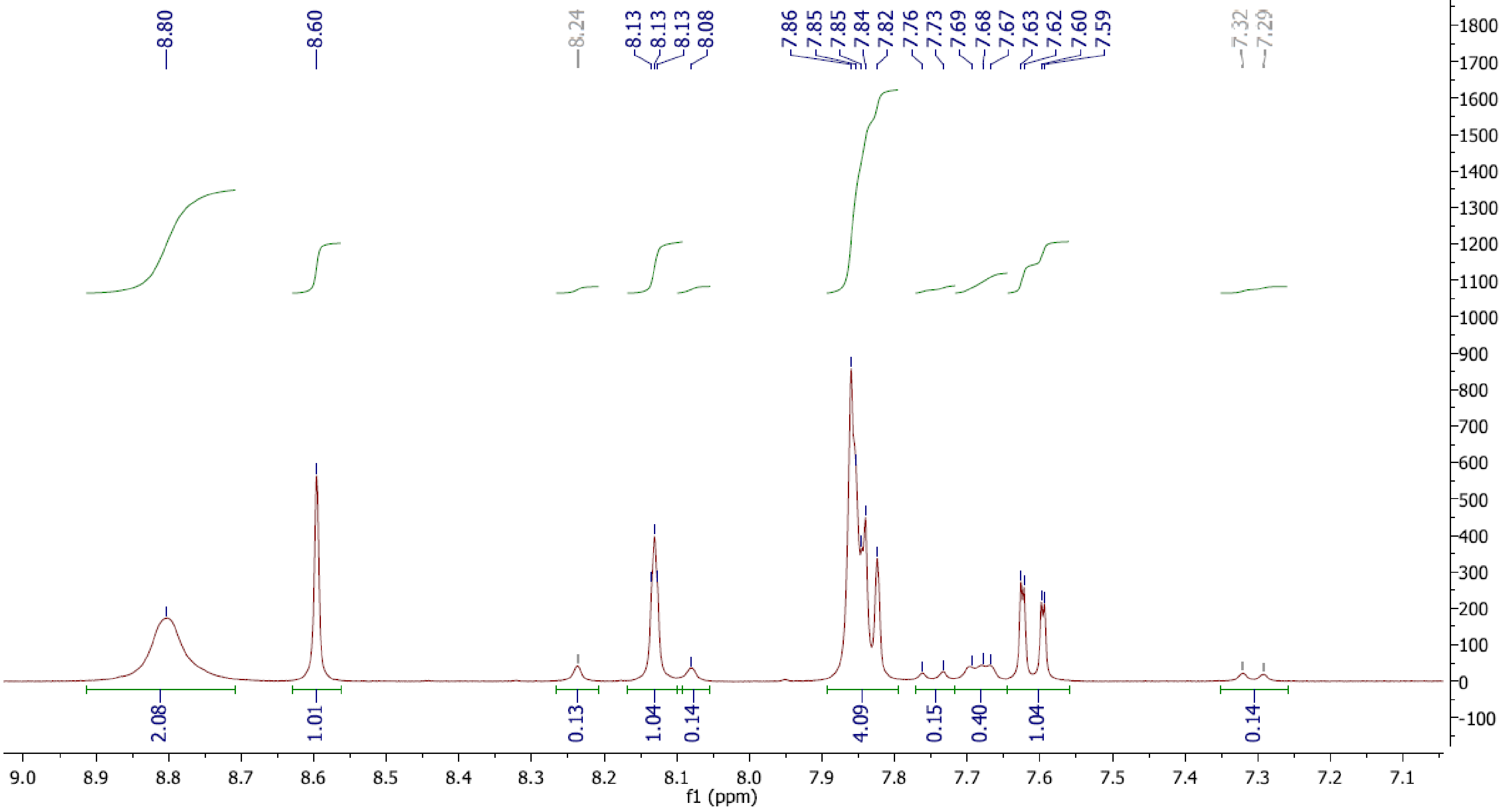

8.80
8.60
8.24
8.13
8.13
8.13
8.08
7.86
7.85
7.85
7.84
7.82
7.76
7.73
7.69
7.68
7.67
7.63
7.62
7.60
7.59
7.32
7.29
2.08
1.01
0.13
1.04
0.14
4.09
0.15
0.40
1.04
0.14
f1 (ppm)

^{13}C NMR

5

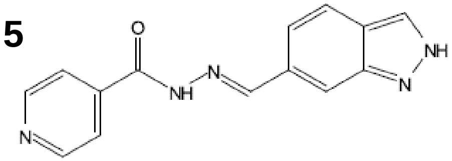

O
NH
N
N
NH
N

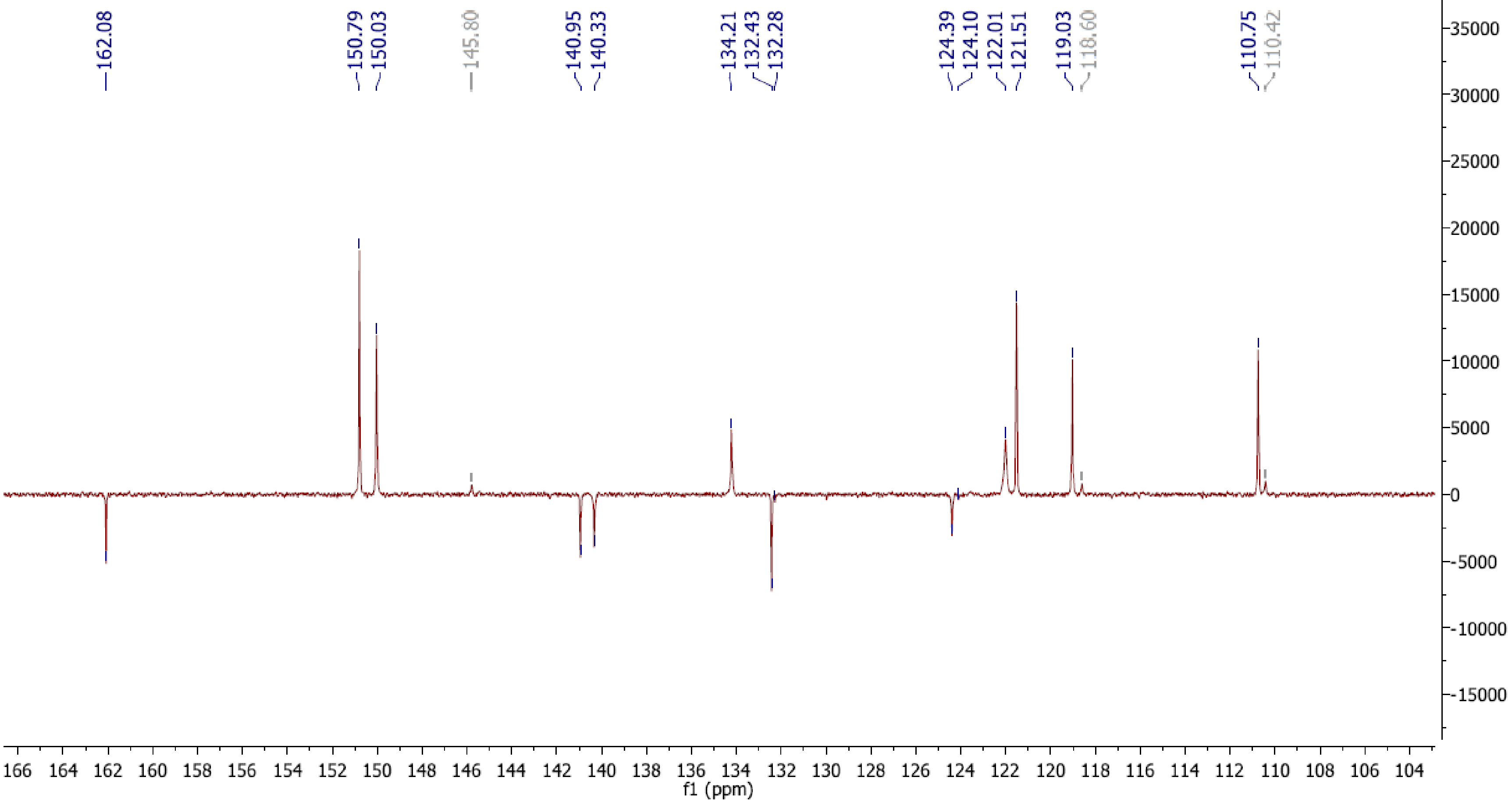

162.08
150.79
150.03
145.80
140.95
140.33
134.21
132.43
132.28
124.39
124.10
122.01
121.51
119.03
118.60
110.75
110.42
50000
45000
40000
35000
30000
25000
20000
15000
10000
5000
0
-5000
-10000
-15000
166 164 162 160 158 156 154 152 150 148 146 144 142 140 138 136 134 132 130 128 126 124 122 120 118 116 114 112 110 108 106 104
f1 (ppm)

^{1}H NMR

6

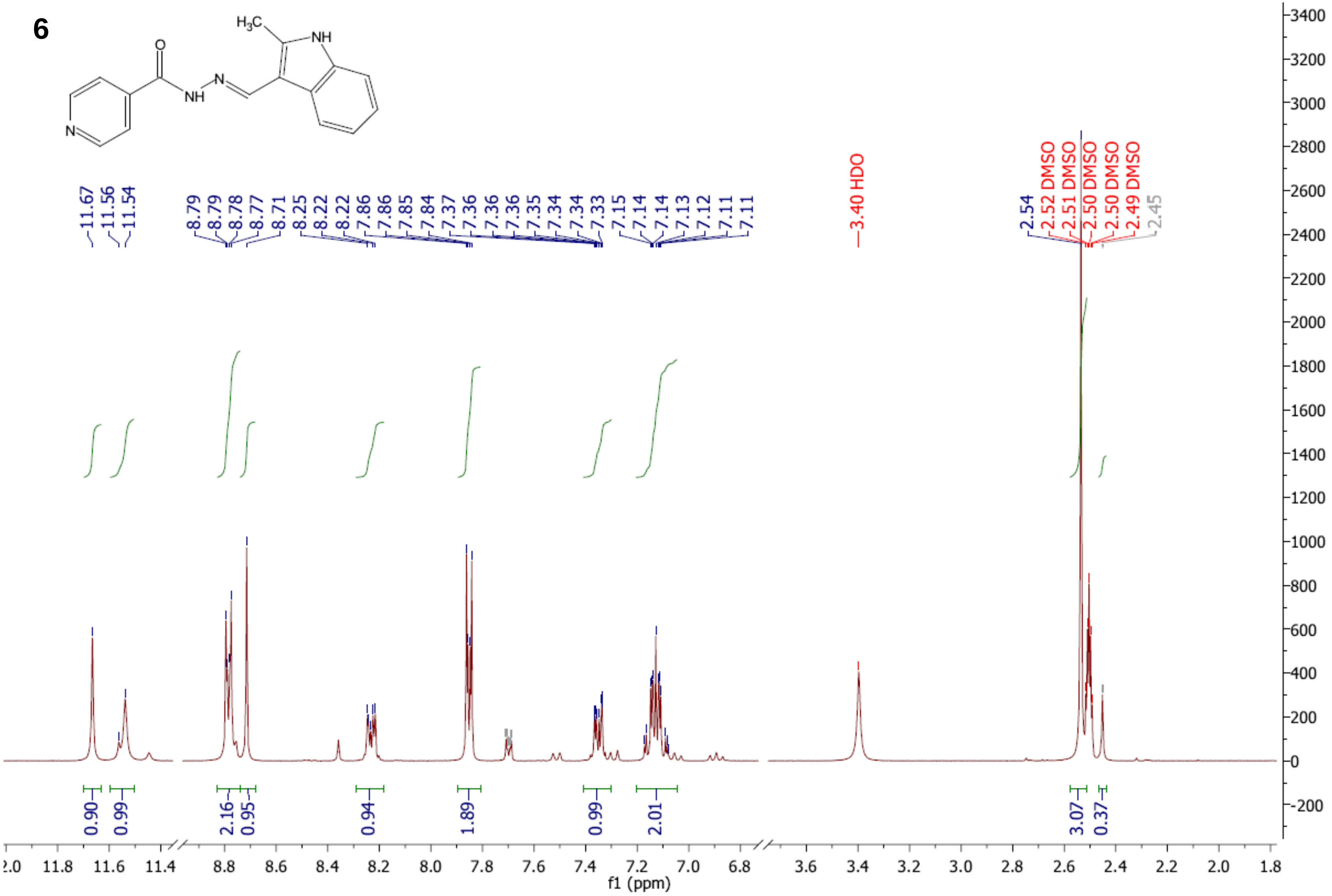

H3C
NH
O
N
NH
N
11.67
11.56
11.54
8.79
8.79
8.78
8.77
8.71
8.25
8.22
8.22
7.86
7.86
7.85
7.84
7.37
7.36
7.36
7.36
7.35
7.34
7.34
7.33
7.15
7.14
7.14
7.13
7.12
7.11
7.11
3.40 HDO
2.54
2.52 DMSO
2.51 DMSO
2.50 DMSO
2.50 DMSO
2.49 DMSO
2.45
0.90
0.99
2.16
0.95
0.94
1.89
0.99
2.01
3.07
0.37
f1 (ppm)

^{13}C NMR

6

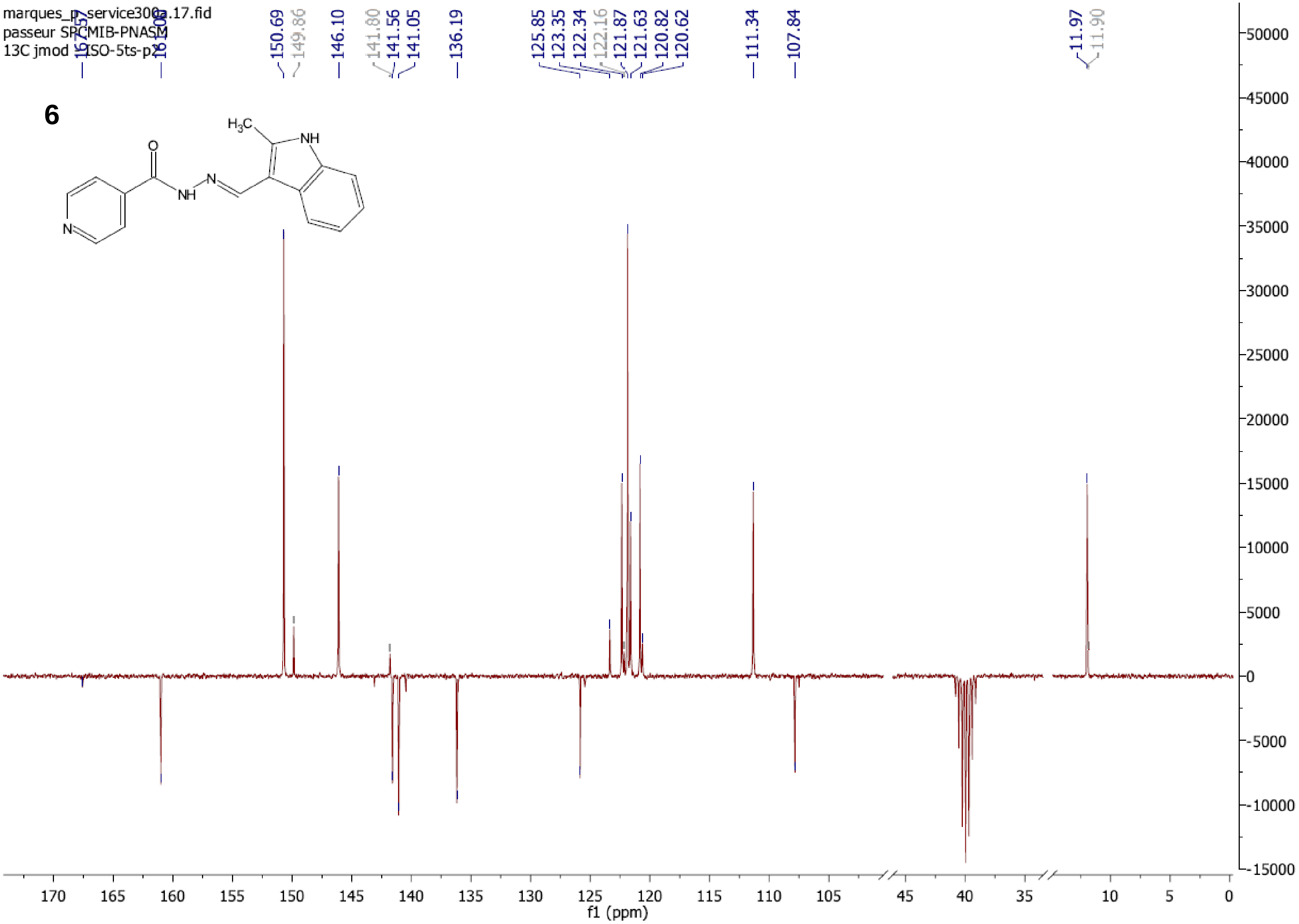

marques_p_service300a.17.fid
passeur SRCMIB-PNASM
13C jmod ISO-5ts-p2
167.57
161.00
150.69
149.86
146.10
141.80
141.56
141.05
136.19
125.85
123.35
122.34
122.16
121.87
121.63
120.82
120.62
111.34
107.84
11.97
11.90
H3C
NH
O
NH
N
N
f1 (ppm)

^{1}H NMR

7

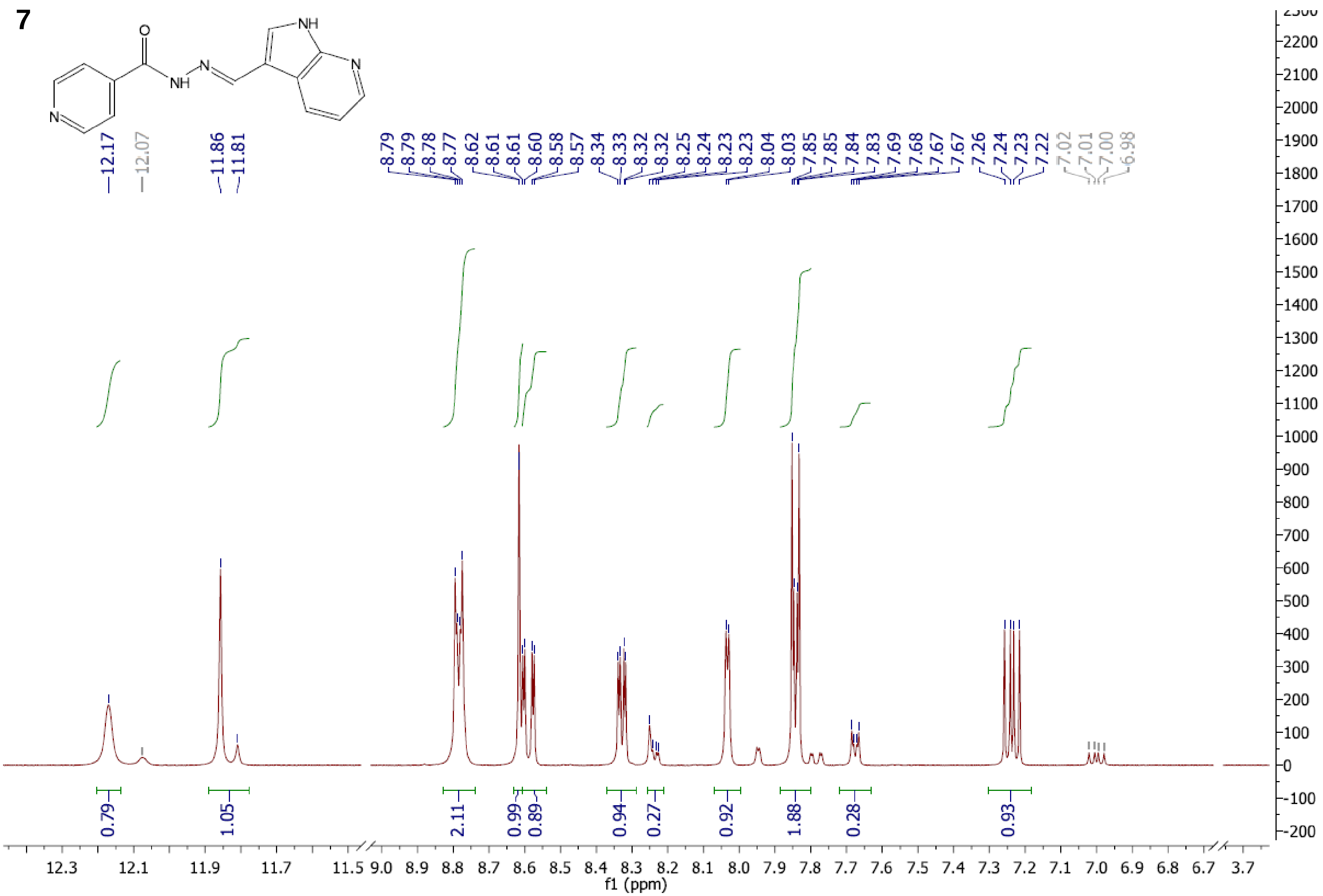

O
NH
N
NH
N
N
12.17
12.07
11.86
11.81
8.79
8.79
8.78
8.77
8.62
8.61
8.61
8.60
8.58
8.57
8.34
8.33
8.32
8.32
8.25
8.24
8.23
8.23
8.04
8.03
7.85
7.85
7.84
7.83
7.69
7.68
7.67
7.67
7.26
7.24
7.23
7.22
7.02
7.01
7.00
6.98
0.79
1.05
2.11
0.99
0.89
0.94
0.27
0.92
1.88
0.28
0.93
f1 (ppm)

^{13}C NMR

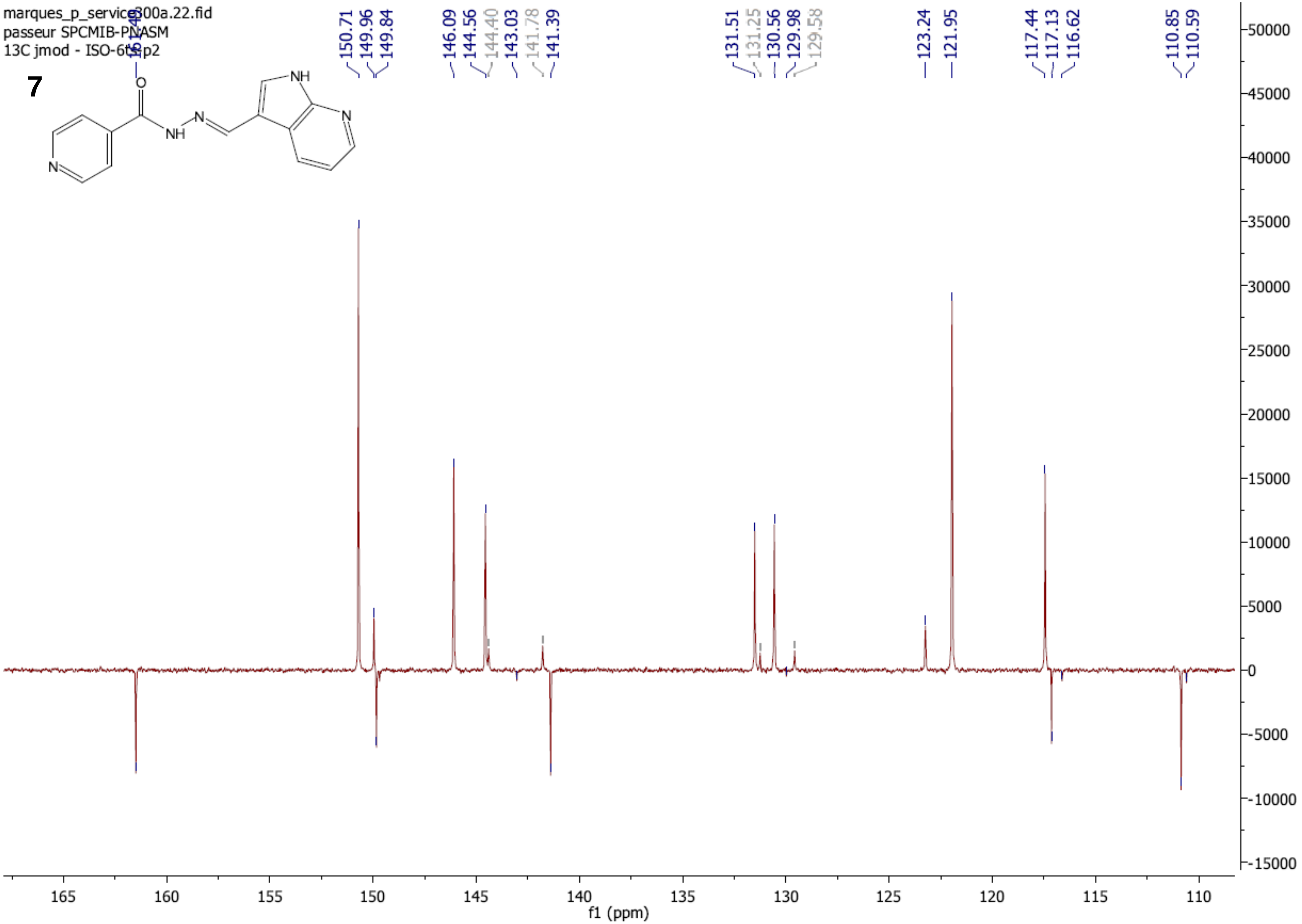
marques_p_service300a.22.fid
passeur SPCMIB-PNASM
13C jmod - ISO-6ts-p2
7
161.40
150.71
149.96
149.84
146.09
144.56
144.40
143.03
141.78
141.39
131.51
131.25
130.56
129.98
129.58
123.24
121.95
117.44
117.13
116.62
110.85
110.59
O
NH
N
NH
N
N
50000
45000
40000
35000
30000
25000
20000
15000
10000
5000
0
-5000
-10000
-15000
165
160
155
150
145
140
135
130
125
120
115
110
f1 (ppm)

^{1}H NMR

8

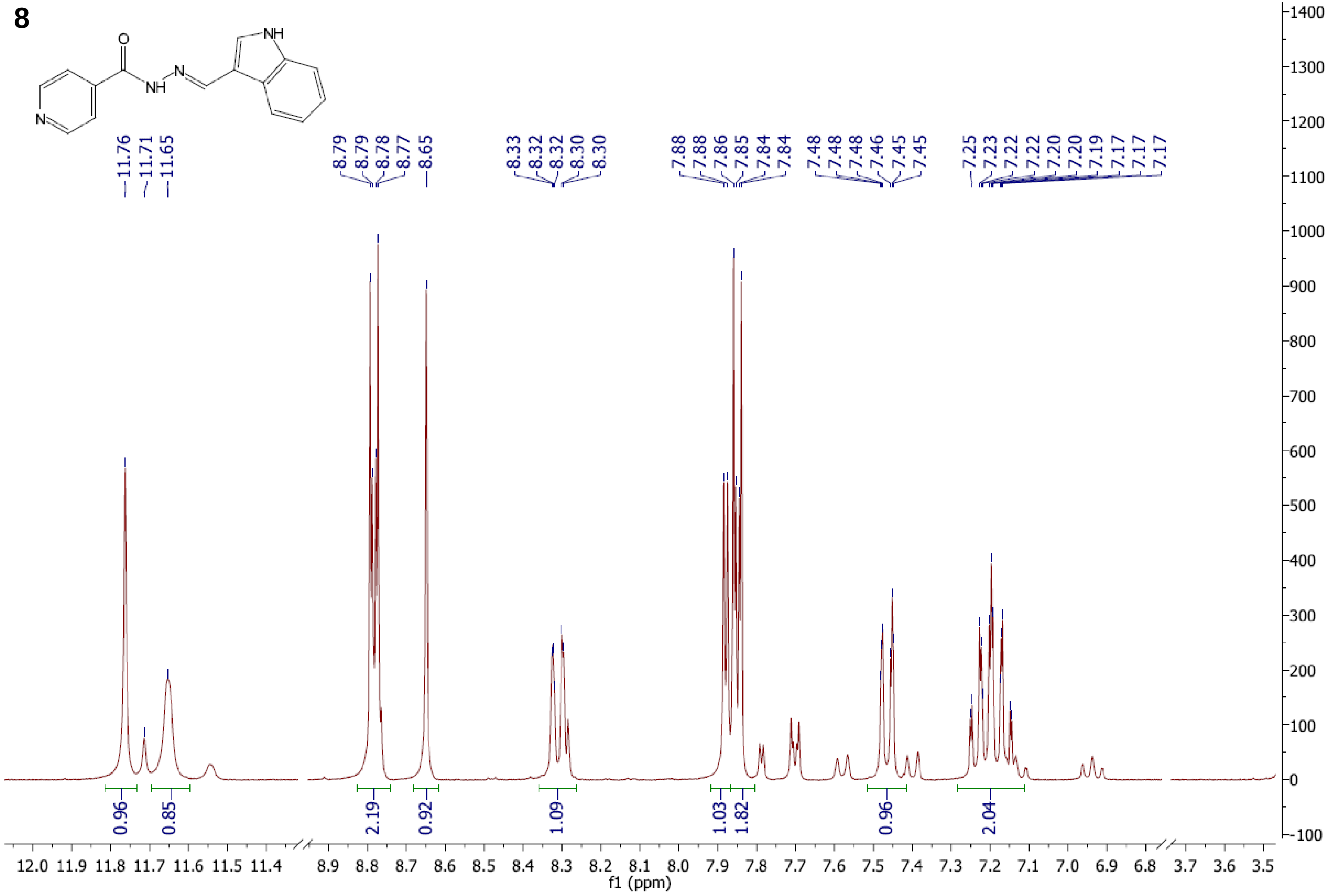

^{13}C NMR

8

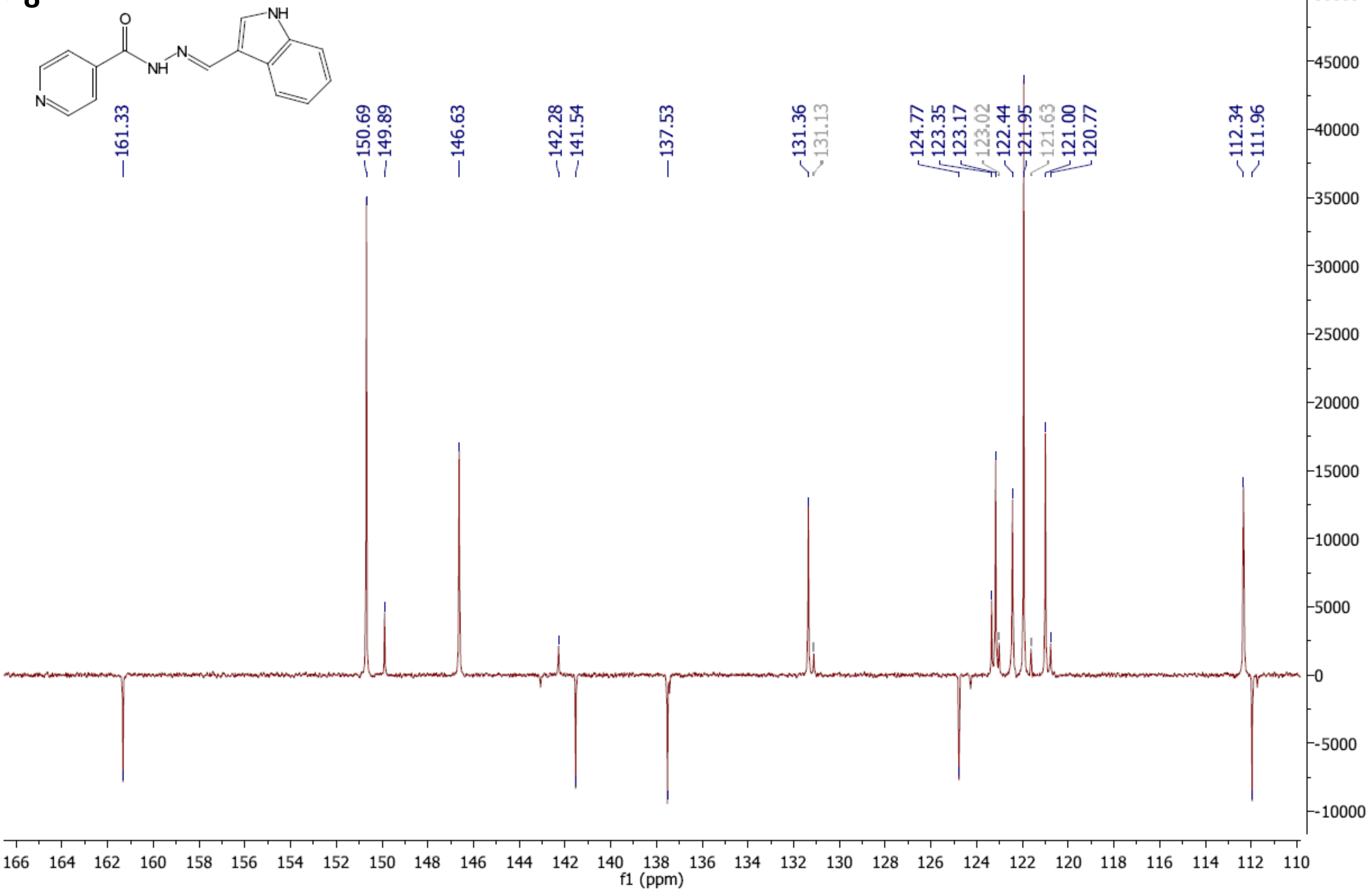
O
NH
N
NH
N
161.33
150.69
149.89
146.63
142.28
141.54
137.53
131.36
131.13
124.77
123.35
123.17
123.02
122.44
121.95
121.63
121.00
120.77
112.34
111.96
50000
45000
40000
35000
30000
25000
20000
15000
10000
5000
0
-5000
-10000
166 164 162 160 158 156 154 152 150 148 146 144 142 140 138 136 134 132 130 128 126 124 122 120 118 116 114 112 110
f1 (ppm)

^{1}H NMR

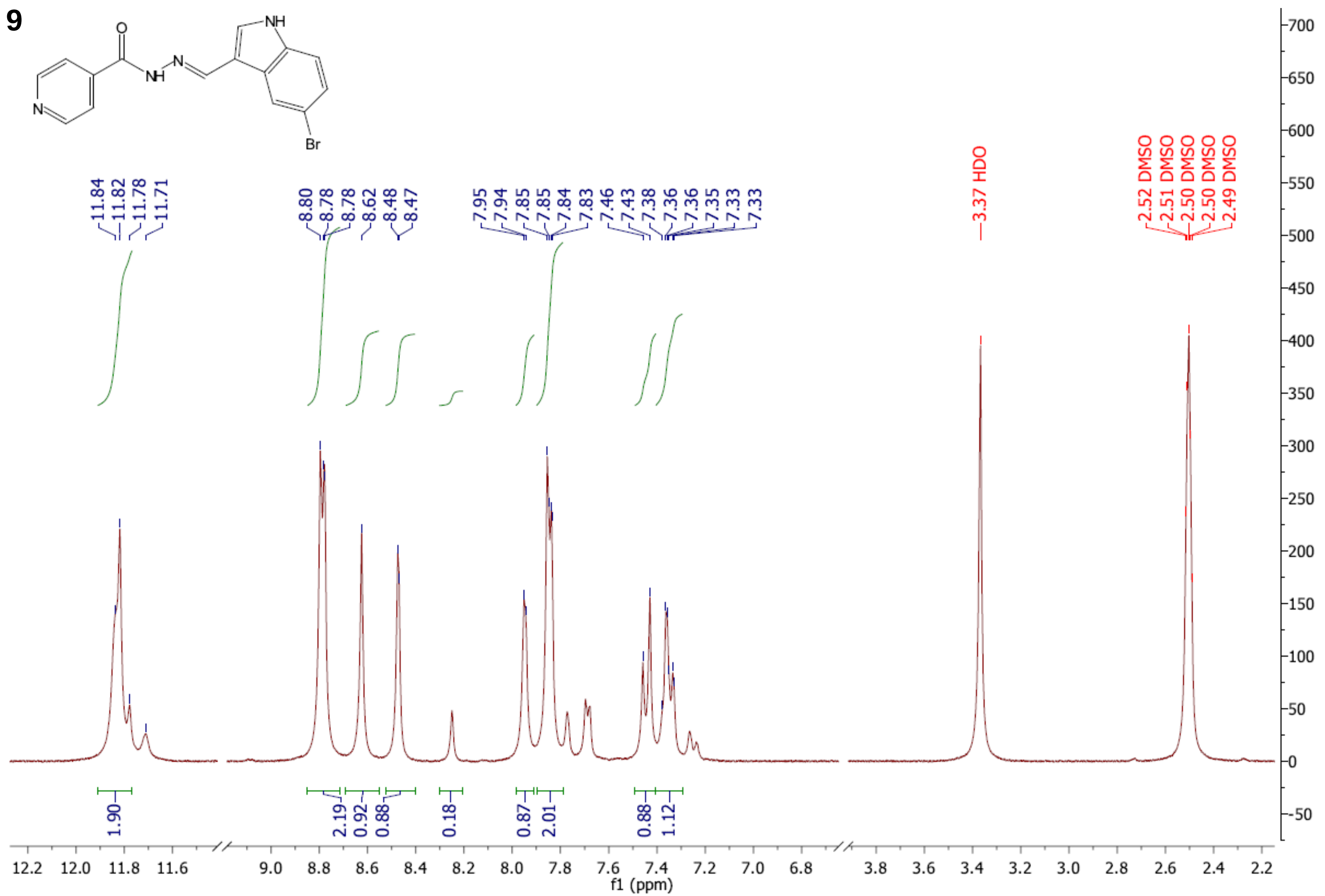

9
NH
O
N
NH
N
Br
11.84
11.82
11.78
11.71
8.80
8.78
8.78
8.62
8.48
8.47
7.95
7.94
7.85
7.85
7.84
7.83
7.46
7.43
7.38
7.36
7.36
7.35
7.33
7.33
3.37 HDO
2.52 DMSO
2.51 DMSO
2.50 DMSO
2.50 DMSO
2.49 DMSO
1.90
2.19
0.92
0.88
0.18
0.87
2.01
0.88
1.12
f1 (ppm)

^{13}C NMR

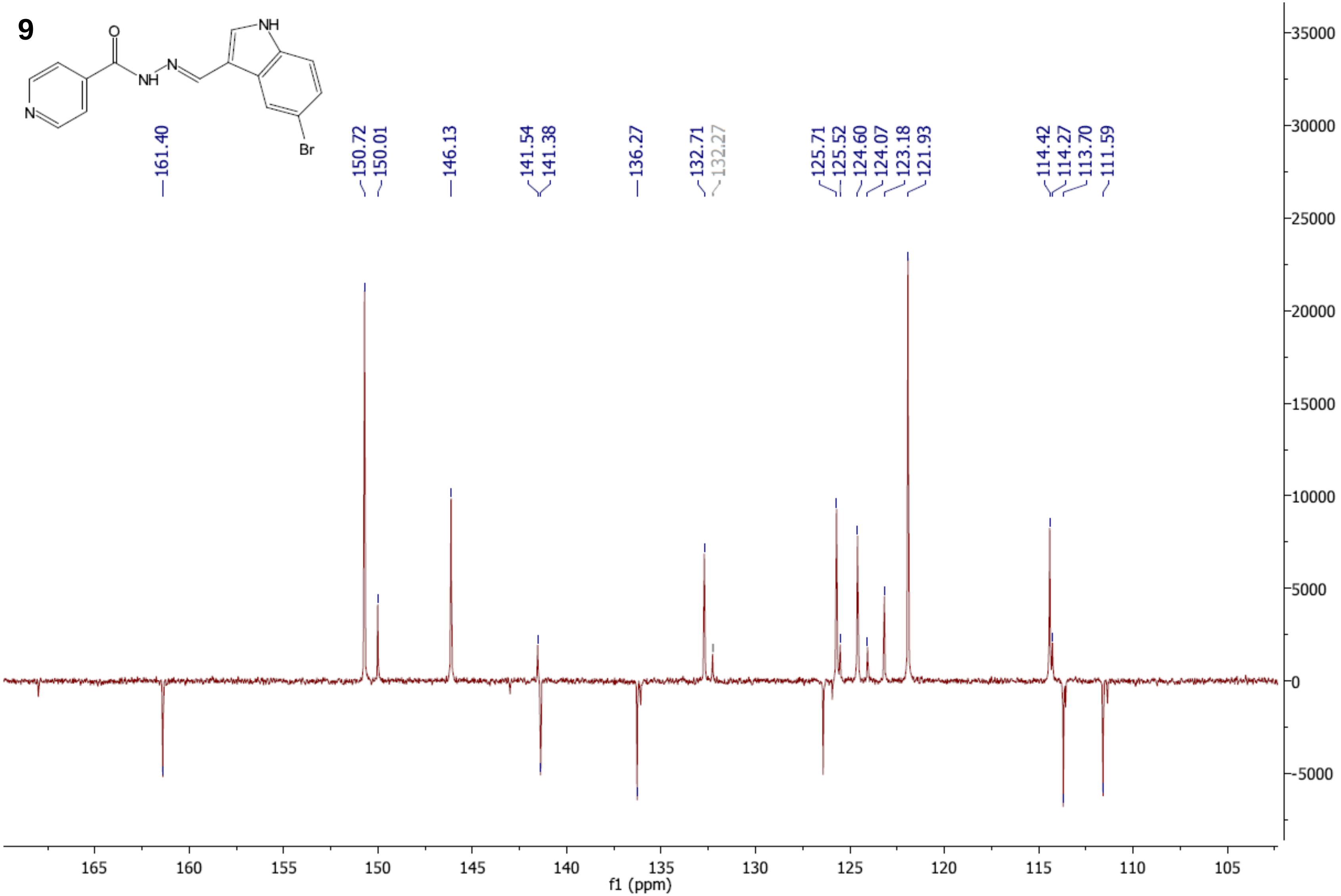

9
O
NH
N
NH
N
Br
161.40
150.72
150.01
146.13
141.54
141.38
136.27
132.71
132.27
125.71
125.52
124.60
124.07
123.18
121.93
114.42
114.27
113.70
111.59
35000
30000
25000
20000
15000
10000
5000
0
-5000
165
160
155
150
145
140
135
130
125
120
115
110
105
f1 (ppm)

^{1}H NMR

10

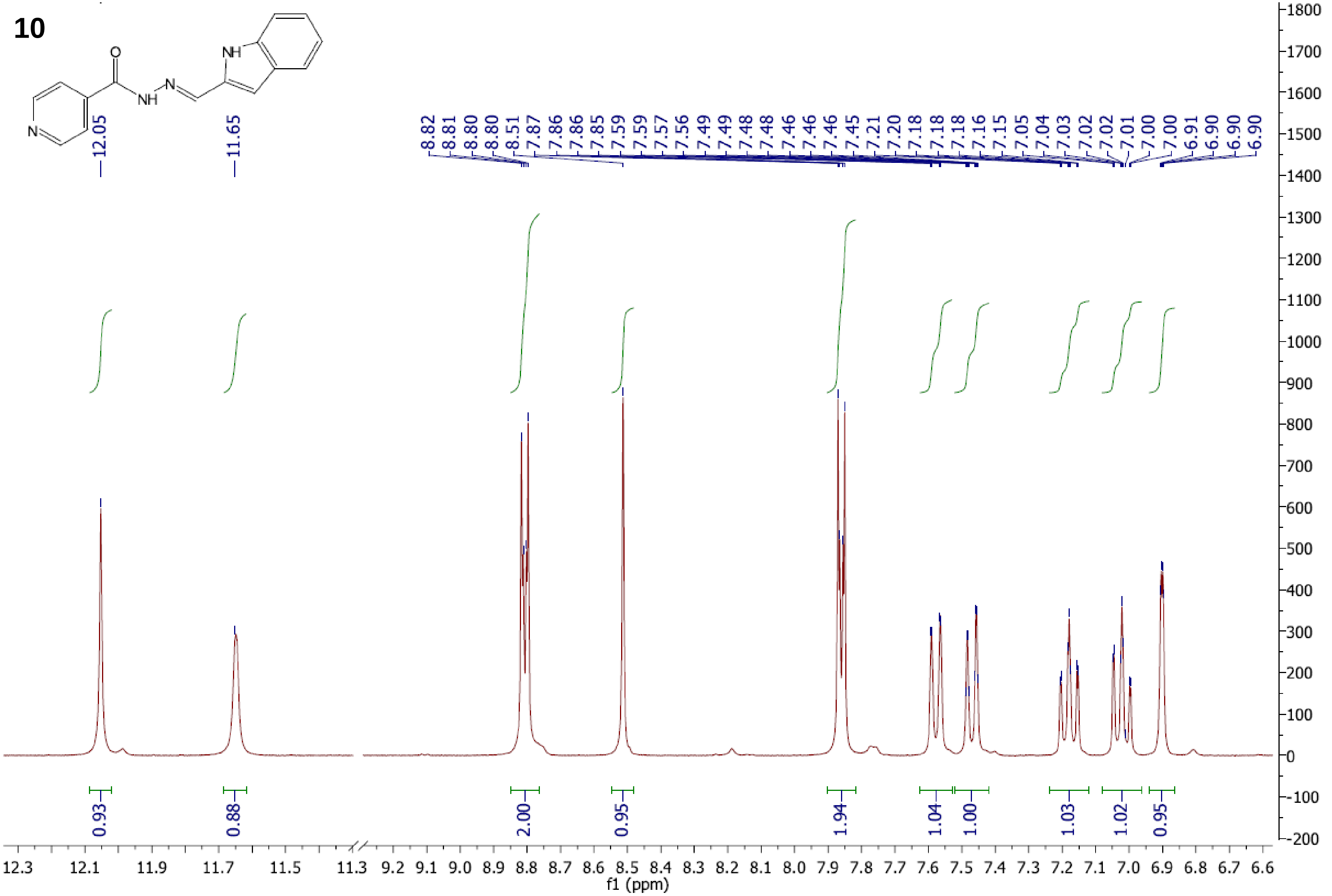

O
NH
N
NH
N
12.05
11.65
8.82
8.81
8.80
8.80
8.51
7.87
7.86
7.86
7.85
7.59
7.59
7.57
7.56
7.49
7.49
7.48
7.48
7.46
7.46
7.46
7.45
7.21
7.20
7.18
7.18
7.18
7.16
7.15
7.05
7.04
7.03
7.02
7.02
7.01
7.00
7.00
6.91
6.90
6.90
6.90
0.93
0.88
2.00
0.95
1.94
1.04
1.00
1.03
1.02
0.95
f1 (ppm)

^{13}C NMR

10

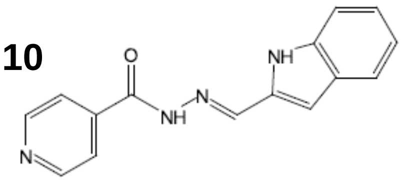

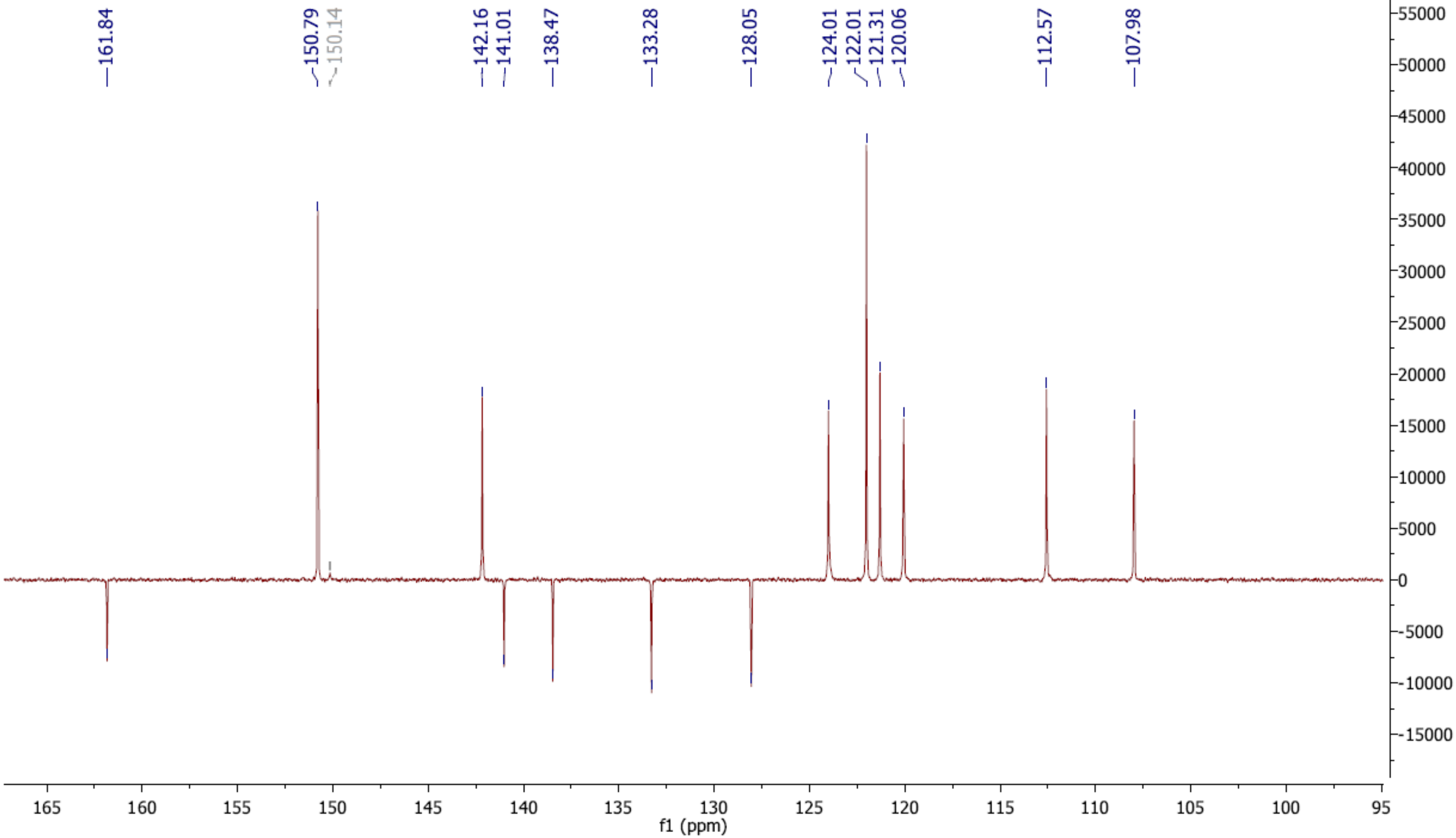

161.84
150.79
150.14
142.16
141.01
138.47
133.28
128.05
124.01
122.01
121.31
120.06
112.57
107.98
f1 (ppm)

^{1}H NMR

11

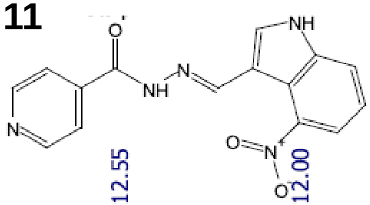

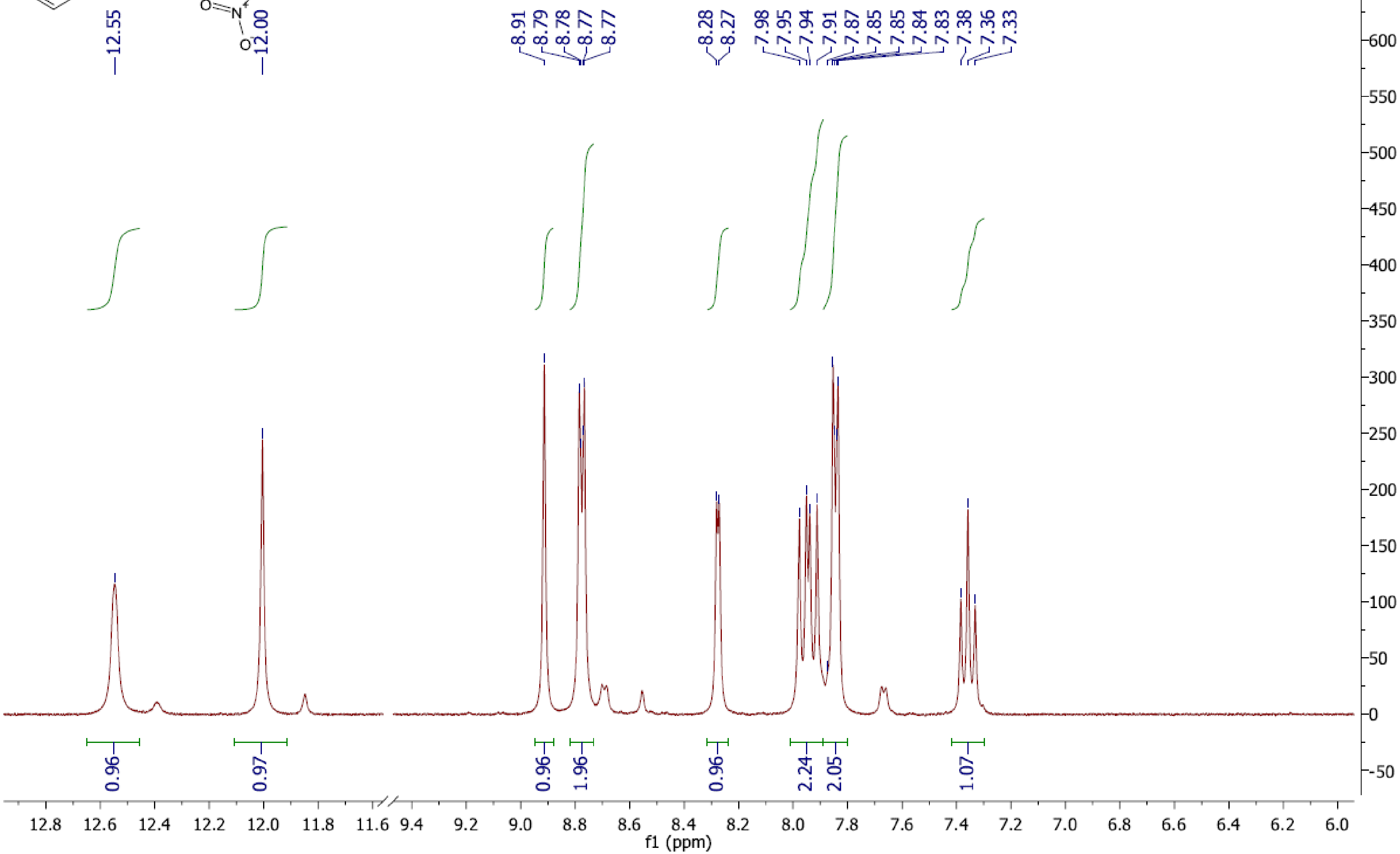

12.55
12.00
8.91
8.79
8.78
8.77
8.77
8.28
8.27
7.98
7.95
7.94
7.91
7.87
7.85
7.85
7.84
7.83
7.38
7.36
7.33
0.96
0.97
0.96
1.96
0.96
2.24
2.05
1.07
f1 (ppm)

^{13}C NMR

11

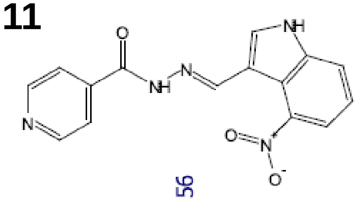

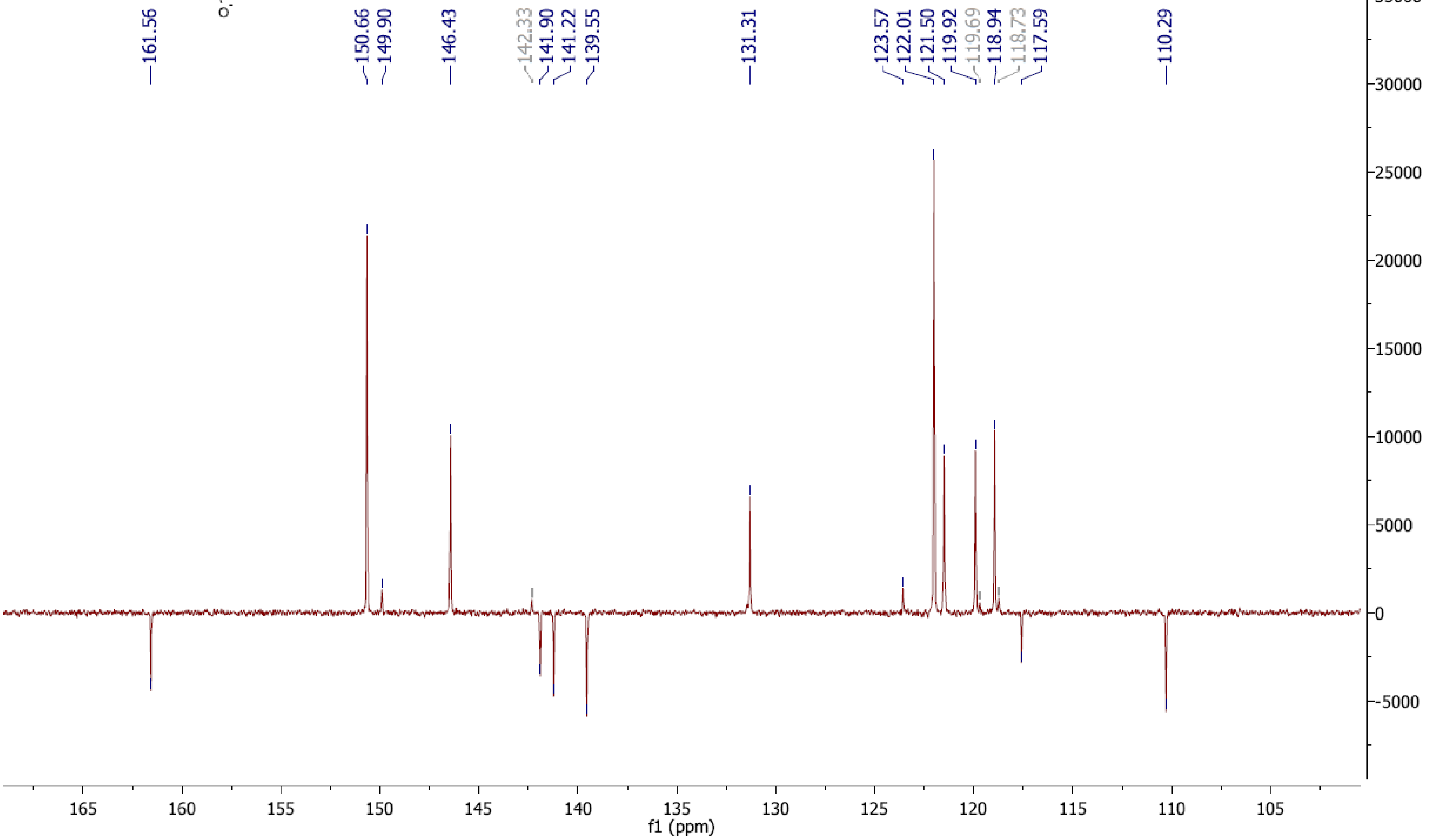

161.56
150.66
149.90
146.43
142.33
141.90
141.22
139.55
131.31
123.57
122.01
121.50
119.92
119.69
118.94
118.73
117.59
110.29
40000
35000
30000
25000
20000
15000
10000
5000
0
-5000
165
160
155
150
145
140
135
130
125
120
115
110
105
f1 (ppm)
